# Aziridination Reactivity of a Manganese(II) Complex with a Bulky Chelating Bis(Alkoxide) Ligand

**DOI:** 10.3390/molecules27185751

**Published:** 2022-09-06

**Authors:** Sudheer S. Kurup, Natalie M. Woodland, Richard L. Lord, Stanislav Groysman

**Affiliations:** 1 Department of Chemistry, Wayne State University, 5101 Cass Ave., Detroit, MI 48202, USA; 2 Department of Chemistry, Grand Valley State University, 1 Campus Drive, Allendale, MI 49401, USA

**Keywords:** alkoxides, aziridination, manganese, iminoiodinane

## Abstract

Treatment of Mn(N(SiMe_3_)_2_)_2_(THF)_2_ with bulky chelating bis(alkoxide) ligand [1,1′:4′,1′′-terphenyl]-2,2′′-diylbis(diphenylmethanol) (H_2_[O-terphenyl-O]^Ph^) formed a seesaw manganese(II) complex Mn[O-terphenyl-O]^Ph^(THF)_2_, characterized by structural, spectroscopic, magnetic, and analytical methods. The reactivity of Mn[O-terphenyl-O]^Ph^(THF)_2_ with various nitrene precursors was investigated. No reaction was observed between Mn[O-terphenyl-O]^Ph^(THF)_2_ and aryl azides. In contrast, the treatment of Mn[O-terphenyl-O]^Ph^(THF)_2_ with iminoiodinane PhINTs (Ts = *p-*toluenesulfonyl) was consistent with the formation of a metal-nitrene complex. In the presence of styrene, the reaction led to the formation of aziridine. Combining varying ratios of styrene and PhINTs in different solvents with 10 mol% of Mn[O-terphenyl-O]^Ph^(THF)_2_ at room temperature produced 2-phenylaziridine in up to a 79% yield. Exploration of the reactivity of Mn[O-terphenyl-O]^Ph^(THF)_2_ with various olefins revealed (1) moderate aziridination yields for *p*-substituted styrenes, irrespective of the electronic nature of the substituent; (2) moderate yield for 1,1′-disubstituted α-methylstyrene; (3) no aziridination for aliphatic α-olefins; (4) complex product mixtures for the β-substituted styrenes. DFT calculations suggest that iminoiodinane is oxidatively added upon binding to Mn, and the resulting formal imido intermediate has a high-spin Mn(III) center antiferromagnetically coupled to an imidyl radical. This imidyl radical reacts with styrene to form a sextet intermediate that readily reductively eliminates the formation of a sextet Mn(II) aziridine complex.

## 1. Introduction

The nitrogen equivalent of an epoxide, aziridine, is a three-membered heterocycle containing one nitrogen and two carbon atoms [1]. The combination of Baeyer strain and an electronegative element in a three-membered ring makes it susceptible to ring-opening reactions [2]. Because of the high reactivity of aziridines, they serve as intermediates in the total synthesis of natural products [3] and pharmaceuticals [4,5], exhibit diverse biological/biomedical functions [6,7], and have been used extensively in the synthesis of various heterocycles [8,9]. Different routes toward this useful functional group have been reported in the literature [1,2,10,11]. In the absence of transition metals, aziridine synthesis requires extreme conditions (high temperature and/or pressure) or the availability of highly reactive ylide-based precursors [12,13,14]. Generally, transition metal-catalyzed nitrene group transfer to olefins enables a more efficient route to aziridines, which also takes place under milder reaction conditions. Various nitrene transfer reagents, such as iminoiodinanes [15,16,17,18], organic azides [19], chloramine–T, and bromamine–T [20,21,22], can act as nitrene precursors to carry out this transformation. Catalysts based on noble metals, such as Ag [23,24,25,26,27,28], Rh [29,30,31,32,33,34,35,36,37], Ru [38,39,40,41,42,43,44], and Pd [45,46,47], were found to be reactive, with a wide variety of alkenes. Although these catalysts are often very efficient, the high cost and low availability of precious metals prompted interest in developing more sustainable catalysts based on earth-abundant and generally less toxic 3d metals [48,49]. Aziridination via nitrene transfer using Mn and Fe porphyrins was reported early on by Breslow et al. [50]. The reactivity of porphyrinoid (porphyrin, corrole, and phthalocyanine), salen, and oxazoline ligands in aziridination has led to a growing interest among the scientific community in developing nitrogen-donor ligand-based aziridination catalysts with varying properties [48]. A significant number of Fe- and Co-based aziridination catalysts have been reported in the last decade, most of which are supported by nitrogen-donor ligands [51,52,53,54,55,56,57,58,59,60,61,62,63,64,65,66]. In recent years, a number of Fe-based aziridination catalysts supported by oxygen-donor [67,68] and carbon-donor [69,70,71,72,73,74,75] ligands have been reported as well. The stability and reactivity of formally high-oxidation state Fe and Co have been demonstrated to depend on ancillary ligand environments. While for weak-field nitrogen and oxygen ligand environments the reactions are generally postulated to proceed via highly reactive (and often not isolable) metal(III)-imidyl radical species, strong-field (carbene) ligation forms more stable Fe(IV) imides [76,77]. In contrast, the field of Mn-based aziridination catalysis has been less explored. Most reported Mn catalysts feature nitrogen-donor ligands [78,79,80,81,82,83,84,85,86,87,88], with the notable exception of the mixed nitrogen/oxygen donor set of salen ligands. The present work describes the aziridination reactivity of a Mn complex bearing an oxygen-ligand environment exclusively.

**Scheme 1:**
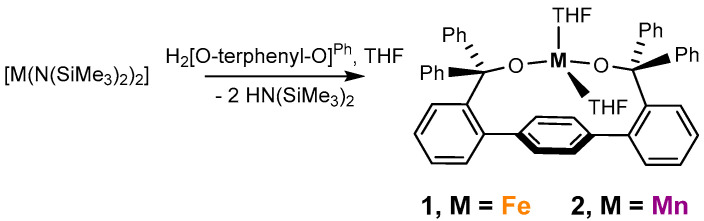
Synthesis of Mn[O-terphenyl-O]^Ph^(THF)_2_ (**2**), along with the previously reported synthesis of iron analogue **1**.

Group-transfer catalysts containing 3d transition metals coordinated with bulky bis(alkoxide) ligands are currently under investigation in our group [89,90]. Bis(alkoxide) ligand platforms provide weak-field ligand environments, leading to reactive nitrene- and carbene-transfer catalysts [91,92,93,94,95,96]. Using these systems, we demonstrated iron- and chromium-mediated nitrene transfer to form azoarenes and carbodiimides, respectively [91,92,93,94]. However, no aziridine formation was observed in the reactions of the chromium or iron systems with the combination of organoazides and styrene. To improve catalyst stability and performance, we recently synthesized a new chelating bis(alkoxide) ligand H_2_[O-terphenyl-O]^Ph^ ([1,1′:4′,1′′-terphenyl]-2,2′′-diylbis(diphenylmethanol)) [97,98]. The resulting iron complex Fe[O-terphenyl-O]^Ph^(THF)_2_ (**1**, Scheme 1) demonstrated significantly more efficient catalytic performance in azoarene production [97]. The improved performance of the chelating bis(alkoxide) ligand [O-terphenyl-O]^Ph^ in catalysis prompted us to investigate the application of its complexes in olefin aziridination. Herein, we report the first example of an Mn-based aziridination catalyst featuring a low-coordinate, oxygen-only, bulky bis(alkoxide) ligand environment.

## 2. Results and Discussion

### 2.1. Reactivity of Fe[O-terphenyl-O]^Ph^(THF)_2_ (***1***) and Synthesis and Characterization of Mn[O-terphenyl-O]^Ph^(THF)_2_ (***2***)

Complex **1** (Fe[O-terphenyl-O]^Ph^(THF)_2_) was prepared as previously described (Scheme 1) [97]. The attempted aziridination using the combination of an organoazide precursor (*p*-tolyl azide), styrene, and **1** (10 mol%) (room temperature) did not produce the desired 2-phenyl-1-(*p*-tolyl)aziridine product (Figure S14). However, replacing organoazide with the more reactive nitrene precursor iminoiodinane (PhINTs, Ts = *p-*toluenesulfonyl) produced 2-phenyl-1-tosylaziridine in 32% yield (Figure S15). In selected cases, Mn-based catalysts exhibited reactivity superior to their Fe-based counterparts [88]. We decided to prepare the Mn(II) analogue of **1** and explore its aziridination reactivity.

Mn[O-terphenyl-O]^Ph^(THF)_2_ (**2**) was synthesized by treating Mn(N(SiMe_3_)_2_)_2_(THF)_2_ with H_2_[O-terphenyl-O]^Ph^ in THF at room temperature (Scheme 1). The purple solution of the Mn(N(SiMe_3_)_2_)_2_(THF)_2_ precursor turned light yellow upon the addition of the ligand over a course of 8 hours. Recrystallization of the crude reaction product from CH_2_Cl_2_/THF mixture at −35 °C produced very pale yellow (nearly colorless) crystals of **2** in 74% yield. The product was characterized by solution magnetic measurements, X-ray crystallography, IR, and UV-vis spectroscopy, and elemental analysis. The solution state magnetic moment of **2** (measured in C_6_D_6_) was found to be 5.8 ± 0.2 µ_B_ (an average of two measurements), consistent with high-spin Mn(II) (Figures S3 and S4). ^1^H NMR spectrum of the paramagnetic Mn(II) complex **2** demonstrated only two broad signals around 1.6 ppm and 3.8 ppm attributable to THF protons (Figures S1 and S2). The UV-vis spectrum in the 350–900 nm range was nearly featureless, as expected for high-spin Mn(II) (Figure S5). 

**Figure 1:**
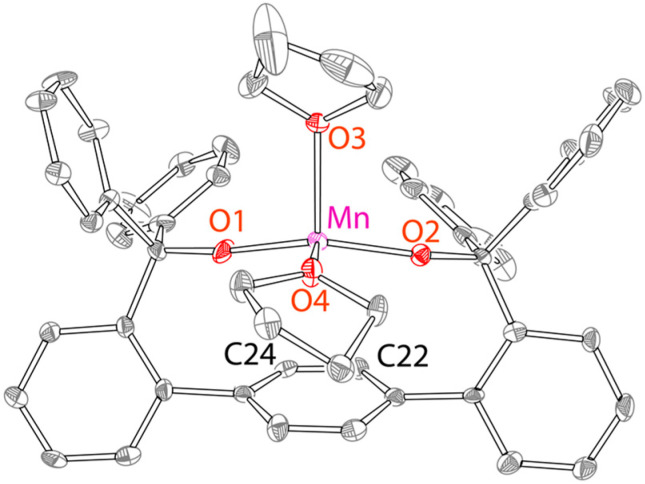
X-ray structure (50% probability) of Mn[O-terphenyl-O]^Ph^(THF)_2_ (**2**). Alternative conformations of one of the top THF ligands and one of the lateral phenyls, H atoms, and disordered solvent were omitted for clarity. Selected bond distances (Å) and angles (°): Mn-O1 1.888(5), Mn-O2 1.922(4), Mn-O3 2.205(4), Mn-O4 2.178(4), O1-Mn-O2 150.3(2), and O3-Mn-O4 91.23(2).

The X-ray structure of **2** is presented in Figure 1 (crystal and refinement data are given in Table S4). As anticipated, [O-terphenyl-O]^Ph^ binds to the metal in a chelating fashion. The geometry at the metal is a distorted seesaw, with a narrow angle between the THF ligands (91.3(2)°) and a wide angle between the alkoxide donors (150.3(2)°) (see Figure 1 for selected bond distances and angles). The seesaw geometry in **2** is similar to the previously reported iron counterpart **1** [97]. The inter-alkoxide angle (RO-Mn-OR, ~150°) in **2** is significantly wider than the corresponding angle in the related Mn(II) complex with two monodentate alkoxides (Mn(OC*^t^*Bu_2_Ph)_2_(THF)_2_, ~130°) [99]. A similar difference in this angle was observed for the corresponding iron isologues Fe[O-terphenyl-O]^Ph^(THF)_2_ (**1**) and Fe(OC*^t^*Bu_2_Ph)_2_(THF)_2_), [97,99,100] and likely resulted from the rigidity of the chelating para-terphenyl bis(alkoxide) framework. The distance between the central phenyl and Mn is 3.09 Å, suggesting no interaction between the phenyl ring and the metal center, as previously observed for the iron analogue [97]. In contrast, a considerably shorter distance between the metal and the central phenyl (2.49 Å) in the chromium analogue Cr[O-terphenyl-O]^Ph^(THF)_2_, was consistent with a weak covalent interaction between chromium and the central phenyl [98].

### 2.2. Reactivity of 2 in Aziridination

Following the synthesis and characterization of **2**, its reactivity in nitrene transfer was investigated. The reaction of **2** with representative organic aryl azides, including mesityl azide, *p-*tolyl azide, and tosyl azide in benzene, did not display any significant color change, even at elevated temperatures. No change in the ^1^H NMR spectra of the reaction mixtures was observed, further suggesting a lack of reactivity. The reaction of **2** with organic azides in the presence of styrene or isocyanides similarly exhibited no change in the NMR spectrum of the reaction mixture. However, replacing the nitrene precursor with iminoiodinane (PhINTs, Ts = *p-*toluenesulfonyl) produced a rapid color change (colorless to brown). The combination of PhINTs, styrene, and **2** (10 mol%, C_6_D_6_) for 24 h at room temperature led to the formation of the desired aziridine product, with 78% yield. The ^1^H NMR spectrum of the reaction mixture (Figure S15) was relatively clean, exhibiting peaks attributable to the aziridine, styrene, and iodobenzene by-product. The control reaction of styrene (and other alkenes, see below) with PhINTs in the absence of a catalyst under similar reaction conditions formed trace amounts of TsNH_2_; no formation of aziridines was observed.

To optimize catalyst performance, we carried out the aziridination of styrene at varying ratios of catalyst, PhINTs, and styrene (Table 1). In addition, we investigated the effects of different solvents on the reaction outcomes. All reactions were conducted at room temperature for 24 hours. Only small variations in product yields (74–78%) were observed for different ratios of styrene:PhINTs:catalyst in C_6_D_6_ (Table 1). These results are noteworthy, as a large excess of styrene is generally necessary in Mn-catalyzed aziridination reactions. The use of CD_2_Cl_2_ or CD_3_CN as the reaction solvent produced similar yields at a 4:1 ratio of styrene:PhINTs (entries 1–3). However, in sharp contrast to C_6_D_6_, a significant decrease in the product yields was observed in these solvents when the styrene:PhINTs ratio was decreased to 1:1 (entries 6–8). It is feasible that a decrease in the product yields at a lower styrene:PhINTs ratio in polar solvents results from the increased solubility and therefore faster decomposition of reactive PhINTs in these solvents. As the reaction yield remained nearly optimal in benzene under less wasteful reaction conditions (reagent ratio of 1:1), it was used as a solvent in subsequent reactivity studies. Reducing catalyst loading to 5% (entry 5) led to a decline in the aziridine yield to 53%; a significant amount of undissolved PhINTs remained.

**Figure 2:**
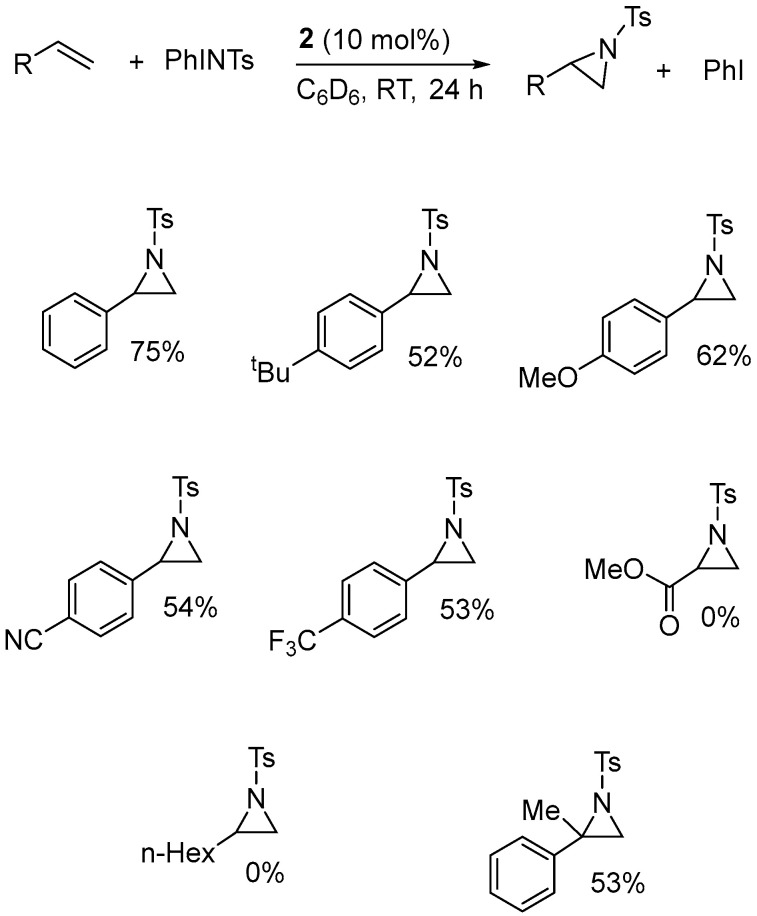
Aziridination of various olefins using **2**. The yields of the aziridine products were determined by ^1^H NMR using internal standard (1,3,5-trimethylbenzene).

Following the optimization of reaction conditions for styrene aziridination, the aziridination of additional olefins was investigated. All reactions were conducted at room temperature for 24 h using 10 mol% catalyst and a ratio of 1:1 PhINTs:styrene. The formation of the products was confirmed by ^1^H NMR spectroscopy and GC-MS (see Supplementary Material). Several different variables in the olefin structure were investigated (Figure 2), including the electronic effect (*para* substitution) in styrene, the aromatic vs. aliphatic nature of mono-substituted olefins, and α- and β-disubstituted styrenes. The catalyst showed moderate reactivity with *p*-*^t^*Bu, *p*-CN, and *p*-CF_3_-substituted styrenes (52–54%); a slightly higher yield (62%) was obtained for electron-rich 4-methoxy styrene. In addition to the aziridine products, NMR spectra demonstrate the presence of an unreacted starting material (styrene) and the expected by-product iodobenzene. No additional products (e.g., TsNH_2_ or C-H aminated products) were observed in significant quantities by ^1^H NMR. However, the formation of these products in small quantities, or the formation of paramagnetic metal-based by-products, cannot be ruled out. The comparable reactivity of electron-rich and electron-poor styrenes in Mn-catalyzed aziridination is consistent with previous reports [88]. No aziridination reactivity with non-aromatic olefins (methyl acrylate and 1-decene, Figures S21 and S22) was observed. 

We also investigated the reactivity of α- and β-disubstituted styrenes. The reaction of α-methyl-styrene produced 1,1’-methylphenylaziridine relatively cleanly, with a moderate yield (53%). However, nitrene transfer onto β-disubstituted styrenes (*cis*/*trans* β-methyl-styrene and *cis*/*trans* stilbene) formed significantly more complex mixtures of products exhibiting broad resonances. Our attempts to isolate the organic products of these reactions were unsuccessful.

### 2.3. Spectroscopic and Computational Probing of the Reaction Mechanism

How does the formation of aziridine occur with **2** as a catalyst? Different mechanistic hypotheses for manganese-mediated aziridination with PhINTs were proposed, including via a Mn-N(Ts)IPh adduct or Mn=NTs imido [85,88]. We probed the nature of reactive intermediates by NMR spectroscopy and DFT calculations. PhINTs is insoluble in the reaction medium. Upon the combination of **2** with PhINTs in C_6_D_6_, its dissolution occurs. The ^1^H NMR spectrum collected immediately after the dissolution of PhINTs (approximately 10–15 min) showed the formation of free PhI (iodobenzene, Figures S7–S9). Thus, the formation of PhI occurs concurrently with the formation of brown intermediates and dissolution of PhINTs, suggesting that Mn[O-terphenyl-O]^Ph^-N(Ts)IPh is not a long-lived reaction intermediate. The addition of styrene after the formation of the brown intermediate shows aziridine formation, suggesting that the nitrene transfer to styrene does not take place via a Mn[O-terphenyl-O]^Ph^-N(Ts)IPh intermediate. No TsNH_2_ was observed by ^1^H NMR after 15 min; formation of TsNH_2_ was observed after approximately 20 h (Figures S10–S12). The UV-vis spectra of the mixtures of **2** with PhINTs, taken at different time intervals, were not informative (Figure S13). Because our multiple attempts to isolate reaction intermediates were not successful, we used DFT calculations to probe the nature of the reaction intermediates and the reaction mechanism.

Additional support for the short-lived iminoiodinane adduct comes from computations. DFT calculations of putative iminoiodinane adducts showed significant activation, as evidenced by the long N-I distances of 2.94 and 3.10 Å for the two isomers vs. 2.06 Å in free iminoiodinane (Figure S28). Mayer bond orders of only 0.08 and 0.05 confirm that the N-I bond is broken in both isomers. Moreover, the electronic structure of this species is nearly identical to that of the formal imido species (*vide infra*) formed after the loss of PhI. The reaction of **2** with two equivalents of PhINTs is unlikely due to steric constraints [86,98]. We hypothesize that the aziridination of styrene takes place via a Mn-imido intermediate. 

**Figure 3:**
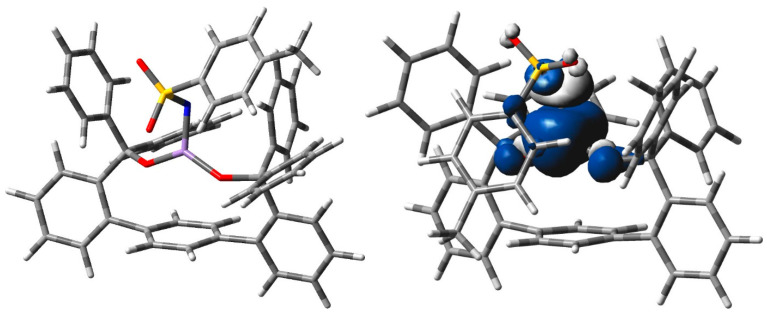
Optimized structure of the lowest energy quartet (**left**) and its spin density isosurface plotted using a cutoff value of 0.002 au (**right**). Blue and white surfaces correspond to excess alpha and beta spins, respectively.

Multiple geometries and possible oxidation/spin states were evaluated for the manganese imido species. The lowest energy quartet optimized structure is shown in Figure 3. This species has a short Mn-NTs bond length of 1.72 Å that is slightly longer than the known Mn-imido species of ~1.6 Å [101,102,103] and shorter Mn-O bond lengths of 1.75 and 1.78 Å than the crystal structure of the Mn(II) species (Figure 1). The spin isosurface shows that spin is mostly localized at Mn and N, with opposite spins on each atom in Figure 3; Mulliken spin densities are 3.5 for Mn, 0.0 for [O-terphenyl-O]^Ph^, and −0.5 for NTs. This suggests that this quartet has an electronic structure between a formal high-spin Mn(IV)-imido and high-spin Mn(III)-imidyl, with antiferromagnetic coupling between the metal and ligand radical. This geometric and electronic structure is similar to the one reported for Mn with a triphenylamido amine ligand; thus, we consider it Mn(III)-imidyl going forward and computationally probe the radical mechanism previously proposed [88]. Analysis of the electronic structure of the iminoiodinane adduct showed Mulliken spins of 3.4 for Mn, 0.0 for [O-terphenyl-O]^Ph^, and −0.4 for TsNIPh, and visualization of the spin density (see Supplementary Material) shows that it is localized on the N. Thus, that adduct is probably better interpreted as manganese imidyl with a van der Waals-bound PhI.

**Figure 4:**
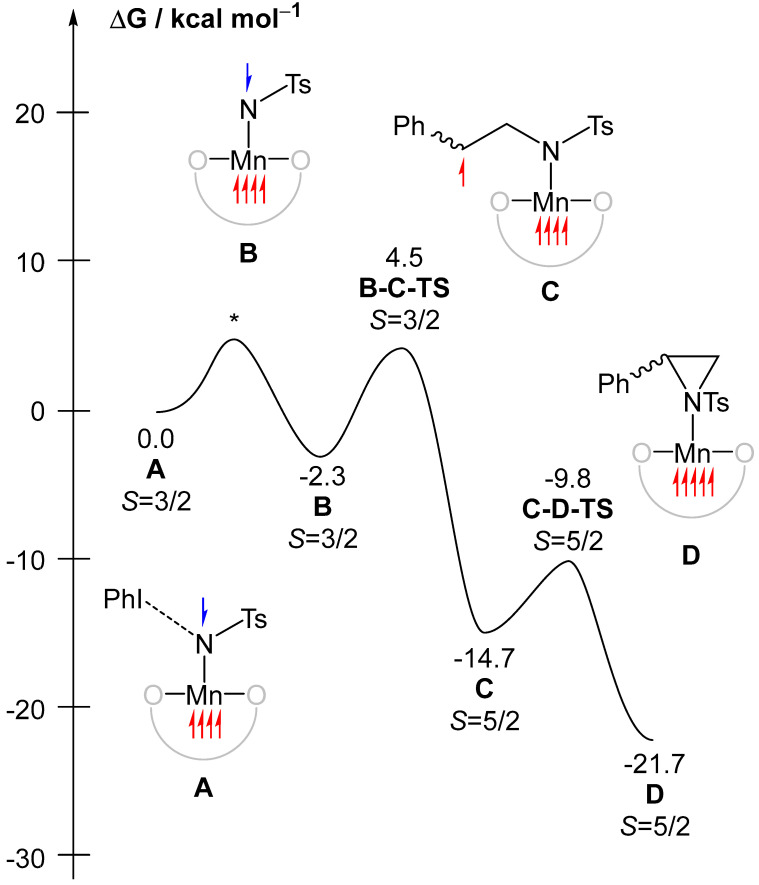
Reaction energy profile calculated at the B3LYP-D3(BJ)/def2-TZVP//BP86-D3(BJ)/def2-SVP level of theory. * indicates a missing transition state. Up and down spins are indicated by red and blue electrons, respectively.

Figure 4 summarizes the lowest energy structures along the reaction mechanism. Alternative regio and stereoisomers, and spin states are discussed in the Supporting Information. Starting with the bis-THF complex, the loss of both THF molecules is uphill by 13.2 kcal/mol. Binding of iminoiodinane to Mn[O-terphenyl-O]^Ph^ is exergonic by 28.3 kcal/mol and affords putative iminoiodinane adduct **A**, which we use as the reference point for this mechanism. As discussed above, **A** has a nearly identical electronic structure to Mn(III)-imidyl intermediate **B**, which is exergonic by 2.3 kcal/mol. **B** reacts with styrene on the quartet surface through a transition state that is 6.8 kcal/mol uphill in Gibbs free energy, and spin crossover occurs before the radical intermediate **C**, which is downhill in Gibbs energy by 12.4 kcal/mol relative to **B**. **C** has Mulliken spins of 4.0 for Mn, 0.0 for [O-terphenyl-O]^Ph^, and 0.9 for NTsCH_2_CHPh, the latter of which is mostly localized on CHPh (see Supplementary Material). The barrier between **C** and **D** is uphill by 4.9 kcal/mol, and **D** is exergonic by 7.0 kcal/mol. **D** has Mulliken spins of 4.8 for Mn, 0.1 for [O-terphenyl-O]^Ph^, and 0.1 for aziridine. Thus, the high-spin Mn(III) intermediate **C** is well poised to reductively eliminate aziridine and re-form sextet Mn(II), **D**, in a spin-allowed step. This mechanism agrees well with the previously proposed mechanism [88].

**Scheme 2:**
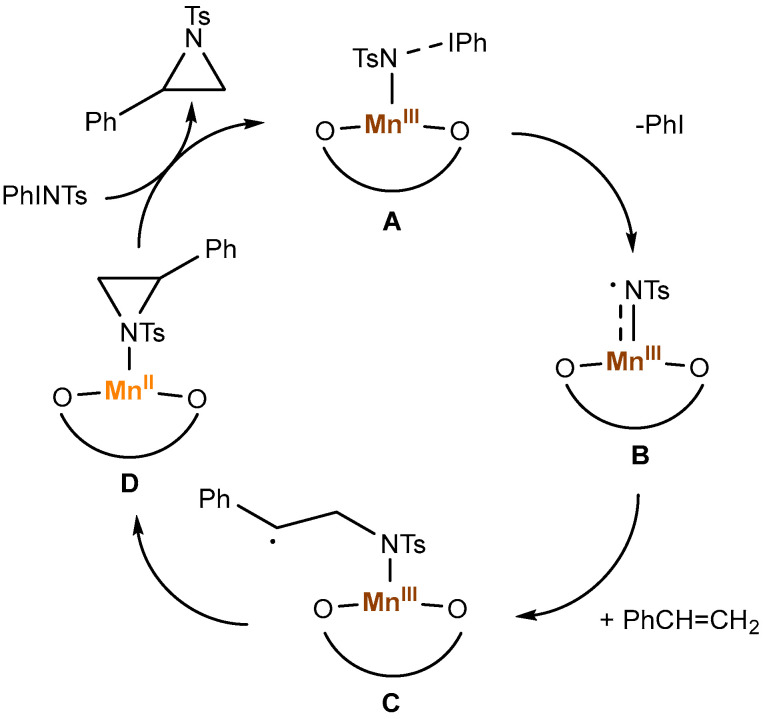
Possible mechanism for styrene aziridination catalyzed by **2**.

The possible catalytic cycle (combining the spectroscopic and computational findings described above) is given in Scheme 2 below. The formation of PhINT adduct **A** is followed by the dissociation of PhI and facile formation of Mn(III)-imidyl radical **B** (free PhI is observed by NMR). While our computational studies focused on the reaction path in which PhINTs replace both THF ligands, it is possible that one THF remains coordinated to the metal throughout the reaction mechanism. Next, reactive (high-energy) species **B** reacts with styrene to form lower-energy radical intermediate **C**. As **C** is significantly more stable and the reaction barrier is low, little discrimination is observed between different para-substituted styrenes (featuring different electronic effects in the sterically remote para position). However, the reaction is anticipated to be sensitive to a more pronounced steric effect, including styrene di-substitution. Likely due to the reactive radical nature of the reaction, complex mixtures of the reaction products were observed for the β-disubstituted styrenes. Subsequent C---N coupling forms azidine adduct **D**, which restores **A**, liberating the aziridine product.

## 3. Summary and Conclusions

We have reported the synthesis and aziridination reactivity of a new manganese complex Mn[O-terphenyl-O]^Ph^(THF)_2_, ligated by a bulky chelating bis(alkoxide) ligand. While the complex did not exhibit a reaction with an organoazide, its treatment with a mixture of PhINTs and styrene led to the formation of 2-phenyl-1-tosylaziridine in a good yield (up to 79%) with 10 mol% of the catalyst at room temperature. Various *p*-substituted styrenes, as well as the α-methyl-styrene produce the corresponding aziridines in comparable moderate yields; no reactivity with aliphatic olefins was observed. DFT calculations suggest a radical reaction intermediate, which is formed upon reaction of the high-spin imidyl radical Mn^III^[O-terphenyl-O]^Ph^(=NTs•) with styrene. Whereas the reactivity of the Mn bis(alkoxide) aziridination catalyst is not superior to the other known Mn aziridination catalysts (described in the Introduction), it (1) allows styrene aziridination relatively efficiently under sustainable (PhINTs:styrene 1:1 ratio) conditions, and (2) represents the first example of nitrene transfer into olefin by a 3d complex in a bulky bis(alkoxide) ligand environment. Our future studies will focus on the additional group-transfer reactions of middle and late 3d complexes in weak-field alkoxide ligand environments, and on the attempts to isolated and structurally and spectroscopically characterize various reaction intermediates. 

## 4. Materials and Methods

### 4.1. General Methods and Characterization

Air-sensitive reactions were carried out in a nitrogen-filled glovebox. MnCl_2_ and KN(SiMe_3_)_2_ were purchased from Strem chemicals and Sigma-Aldrich, respectively, and used as received. 2-bromophenylboronic acid, 1,4-diiodobenzene, and K_2_CO_3_ were purchased from Sigma-Aldrich and used as received. H_2_[O-terphenyl-O]^Ph^ and PhINTs were synthesized based on the literature procedures [97,104]. Benzene-d_6_ was purchased from Cambridge Isotope Laboratories and stored over 3 Å molecular sieves. HPLC-grade non-deuterated solvents were purchased from Sigma-Aldrich and purified using an MBraun solvent purification system. Compounds were generally characterized by ^1^H and ^13^C NMR, high-resolution mass spectrometry, and/or elemental analysis. Selected compounds were characterized by X-ray crystallography. NMR spectra were recorded at the Lumigen Instrument Center on an Agilent 400 MHz Spectrometer and an Agilent DD2-600 MHz Spectrometer in C_6_D_6_ or CDCl_3_ at room temperature. Chemical shifts and coupling constants (*J*) were reported in parts per million and Hertz, respectively. The solution state effective magnetic moment of **2** was determined using the Evans method. Elemental analysis was carried out by Midwest Microlab LLC under air-free conditions. A Thermo Fisher Scientific LTQ Orbittrap XL mass spectrometer at the Lumigen Instrument Centre was used for high-resolution mass spectra. IR spectra of powdered samples were recorded on a Shimadzu IR Affinity-1 FT-IR Spectrometer outfitted with an MIRacle10 attenuated total reflectance accessory with a monolithic diamond crystal stage and pressure clamp. UV-visible spectra were obtained using a Shimadzu UV-1800 spectrometer. GC-MS analysis was carried out using an Agilent 6890N spectrometer, Thermo TG5MS 30 m × 0.32 mm × 0.25 μm column, 7683 series injector, and Agilent 5973 detector.

### 4.2. Synthesis of Mn[O-terphenyl-O]^Ph^(THF)_2_ (***2***) 

A 57 mg solution of ligand (0.096 mmol, 1 eq.) in THF was added dropwise to a 5 mL THF solution of Mn[N(SiMe_3_)_2_]_2_(THF)_2_ [105] (0.050 g, 0.096 mmol, 1 eq.). The color of the reaction changed from purple to light brown over the course of 12 h. The volatiles were removed under vacuum, and the crude product was recrystallized using a DCM-THF solvent mixture at −35 °C to give the complex 88% yield (0.061 mg, 0.077 mmol). IR (cm^−1^, selected peaks) 2942 (m), 2899 (m), 2366 (m), 1490 (s), 1439 (s), 1115 (s), 1173 (s), 1073 (s), 1026 (s), 909 (s), 888 (s), 832 (s), 790 (s), 755 (s), 700 (s), and 638 (s). The UV−vis spectrum was nearly featureless in the 350–800 nm region (Figure S5). The magnetic moment for the compound was determined using the Evans method; two measurements (Figures S1 and S2) yielded µ_eff_ values of 5.68 and 5.97 µ_B._ Anal. Calc for C_52_H_46_MnO_4_: C, 79.07; H, 5.87. Found: C, 79.84; H, 6.21.

### 4.3. General Procedure for Catalytic Formation of Aziridines

A vial containing solid PhINTs (1 eq.) was transferred to a stirred C_6_D_6_ solution containing mesitylene (internal standard), alkene (1 eq.) and Mn[O-terphenyl-O]^Ph^(THF)_2_ (0.1 eq.). The color of the solution changed from colorless to brown. The NMR of the solution was collected after 24 h, and the yield was calculated with reference to the internal standard. Major products were identified by NMR and GC-MS. C_6_D_6_ was removed under vacuum, and the crude mixture was dissolved in CDCl_3_. A comparison of the NMR with literature reports confirmed the formation of aziridines [106,107,108].

### 4.4. Computational Methods

Calculations were performed using density functional theory, as implemented in Gaussian 09 [109]. Geometry optimizations and frequency calculations [110], and wavefunction stability analyses [111] were executed at the BP86-D3(BJ)/def2-SVP level of theory employing density fitting and ultrafine grids [112,113,114,115,116]. Subsequent single-point energy refinements were performed at the B3LYP-D3(BJ)/def2-TZVP level of theory with ultrafine grids [114,117,118,119,120]. Structural analysis and orbital/spin density visualization were done in GaussView 06 [121]. Mayer bond orders were calculated using Multiwfn 3.5 [122].

## Figures and Tables

**Table 1:** Optimization of aziridination conditions.

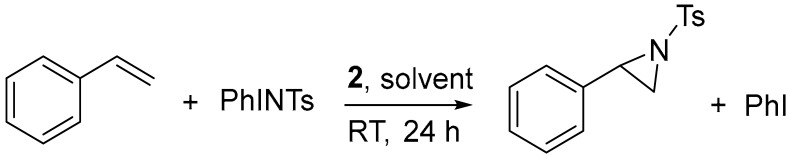					
Entry	2 (mol%)	Solvent	PhINTs (equiv)	Styrene (equiv)	Yield (%)
1	10	C_6_D_6_	1	4	78
2	10	CD_2_Cl_2_	1	4	79
3	10	CD_3_CN	1	4	70
4	10	C_6_D_6_	1	2	74
5	5	C_6_D_6_	1	2	53
6	10	C_6_D_6_	1	1	75
7	10	CD_2_Cl_2_	1	1	17
8	10	CD_3_CN	1	1	43
9	10	C_6_D_6_	2	1	74
10	10	C_6_D_6_	1.5	1	75

## Data Availability

Information about Supplementary material is provided above in “Supplementary Materials” statement.

## Supplementary Materials

The following supporting information can be downloaded at https://www.mdpi.com/article/10.3390/molecules27185751/s1, ^1^H NMR and GC-MS spectra of the aziridine products (Figures S14–S27), characterization data for complex **2** (Figures S1–S13), computational details, including energies, frequencies, and optimized geometries, for all species (Figures S28–S30, Tables S1–S3), crystal and refinement data for **2** (Table S4). CCDC 2183156.
