# Supplementary material for: Aziridination Reactivity of a Manganese(II) Complex with a Bulky Chelating Bis(Alkoxide) Ligand
Source: Molecules. 2022 Sep 6;27(18):5751. doi: 10.3390/molecules27185751 (PMC9505844; doi:10.3390/molecules27185751)
Supplement: Supplementary file 1 [file molecules-27-05751-s001.zip › Revised SI/Mn_aziridination_SI_Molecules_revised.pdf]

Supplementary Material

for

Aziridination Reactivity of a Manganese(II) Complex with a Bulky Chelating Bis(Alkoxide) Ligand

Sudheer Kurup,[a] Natalie M. Woodland,[b] Richard L. Lord,[*b] Stanislav Groysman[*a]

Table of Contents	
1. Characterization of **2** and its stoichiometric reactivity	3
2. NMR spectra of the aziridine products	17
3. GC-MS spectra of the aziridine products	27
4. Computational data	31
5. Table S4: Crystal and refinement data for the structure of **2**	72

1. Characterization of Mn[O-terphenyl-O]^{Ph}(THF)$_2$ (2) and its stoichiometric reactivity

Evans method formula and procedure. Evans method was carried out using a standard NMR tube and a Wilmad coaxial insert (purchased from Aldrich). Carefully weighted (83 and 112 mg) sample of the Mn complex was dissolved in 1 ml of C_6D_6 to prepare known concentrations of solutions which was further used for calculations. The insert with the solution was then placed inside an NMR tube containing C_6D_6. The resulting NMR spectra were used to measure molar susceptibility χ_m. It was calculated using the equation (**1**).

$$\chi_m = [\frac{3\Delta\nu}{4\pi m\nu_0} + \chi_0]M \qquad \text{(Eq. 1)}$$

Where $\Delta\nu$ is the peak separation (Hz), m is the concentration of the solution (g/mL), ν_0 is the spectrometer operating frequency in Hz, $\chi 0$ is the molar susceptibility of the solvent (in cm^3/g), and M is the molar mass of the compound (g/mol). Diamagnetic corrections were calculated using Pascals constants. The solution state effective magnetic moment (μ_{eff}) was calculated using Equation 2:

$$\mu_{eff} = \sqrt{(2.383 \times 10^3)(\chi_m)} \qquad \text{(Eq. 2)}$$

1	5.68
2	5.97

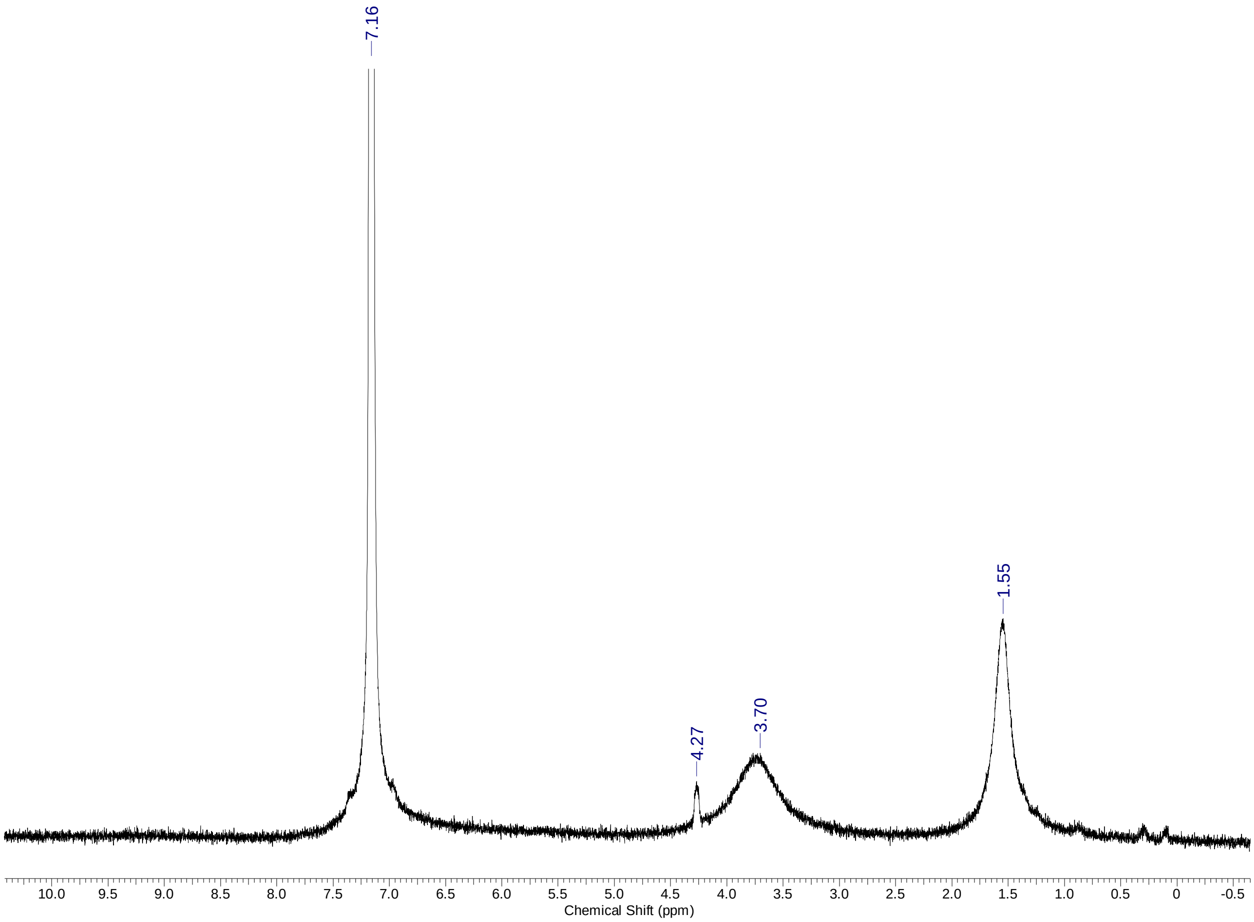

Figure S1. ^{1}H NMR of Mn[O-terphenyl-O]^{Ph}(THF)$_2$ (C_6D_6) in the -0.5 ppm – 10.5 ppm range. Peaks at 1.55 and 3.70 ppm are THF signals and the peak at 4.27 ppm is CH_2Cl_2.

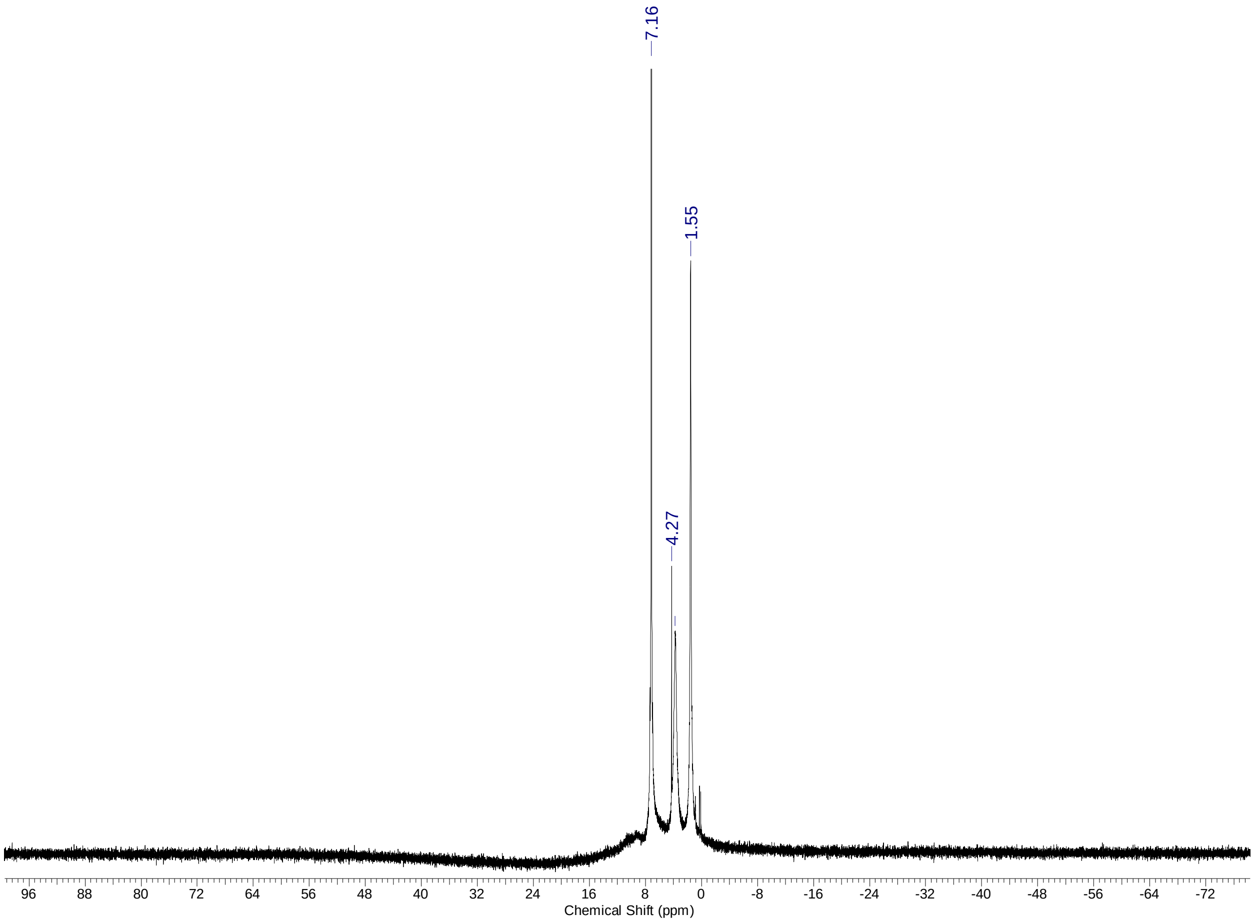

Figure S2. ^{1}H NMR of Mn[O-terphenyl-O]^{Ph}(THF)$_2$ taken in the broad (-72 ppm – 100 ppm) range. Peaks at 1.55 and 3.70 ppm are broad THF signals and 4.27 ppm is CH_2Cl_2.

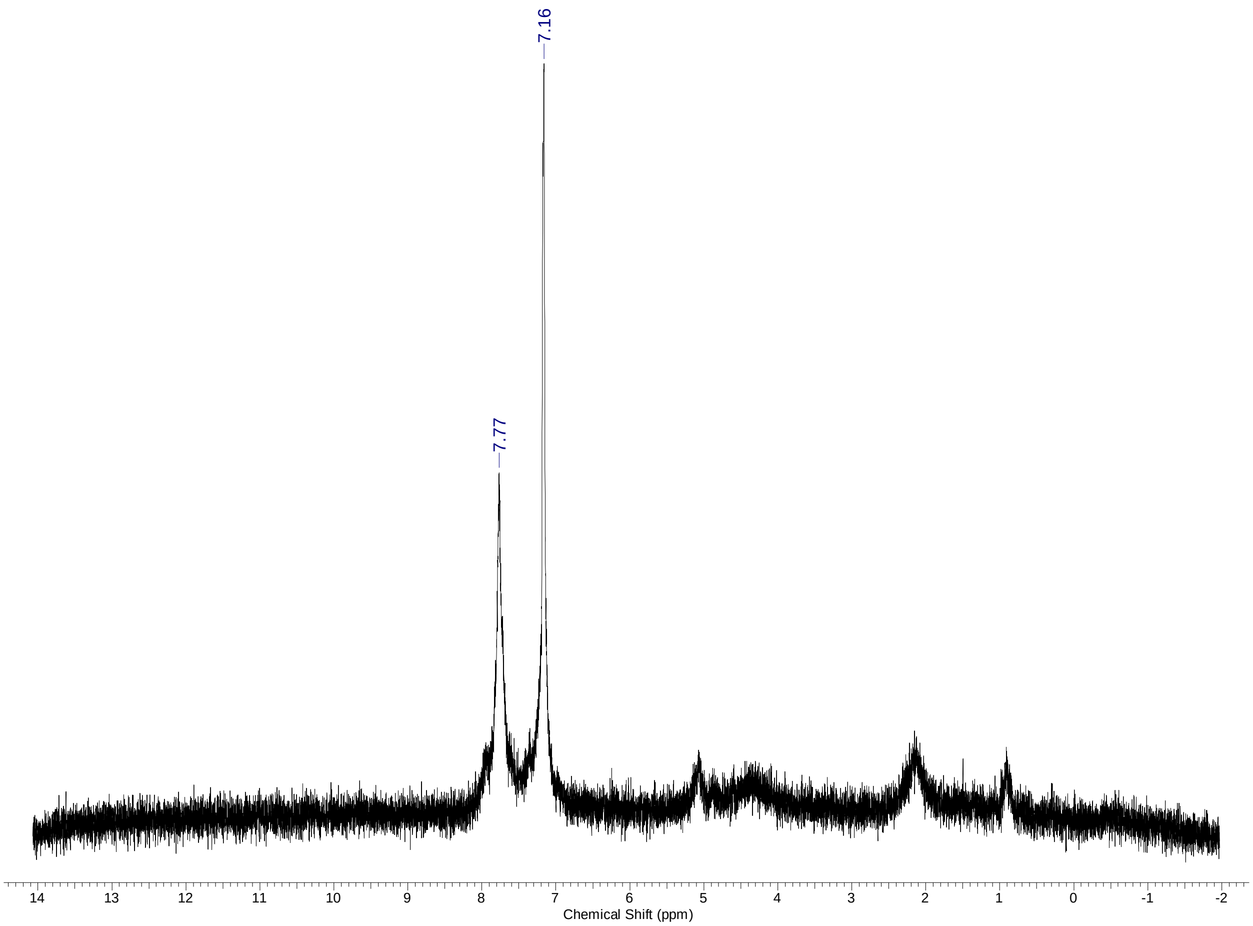

Figure S3. Characterization of **2** by Evans method (experiment #1).

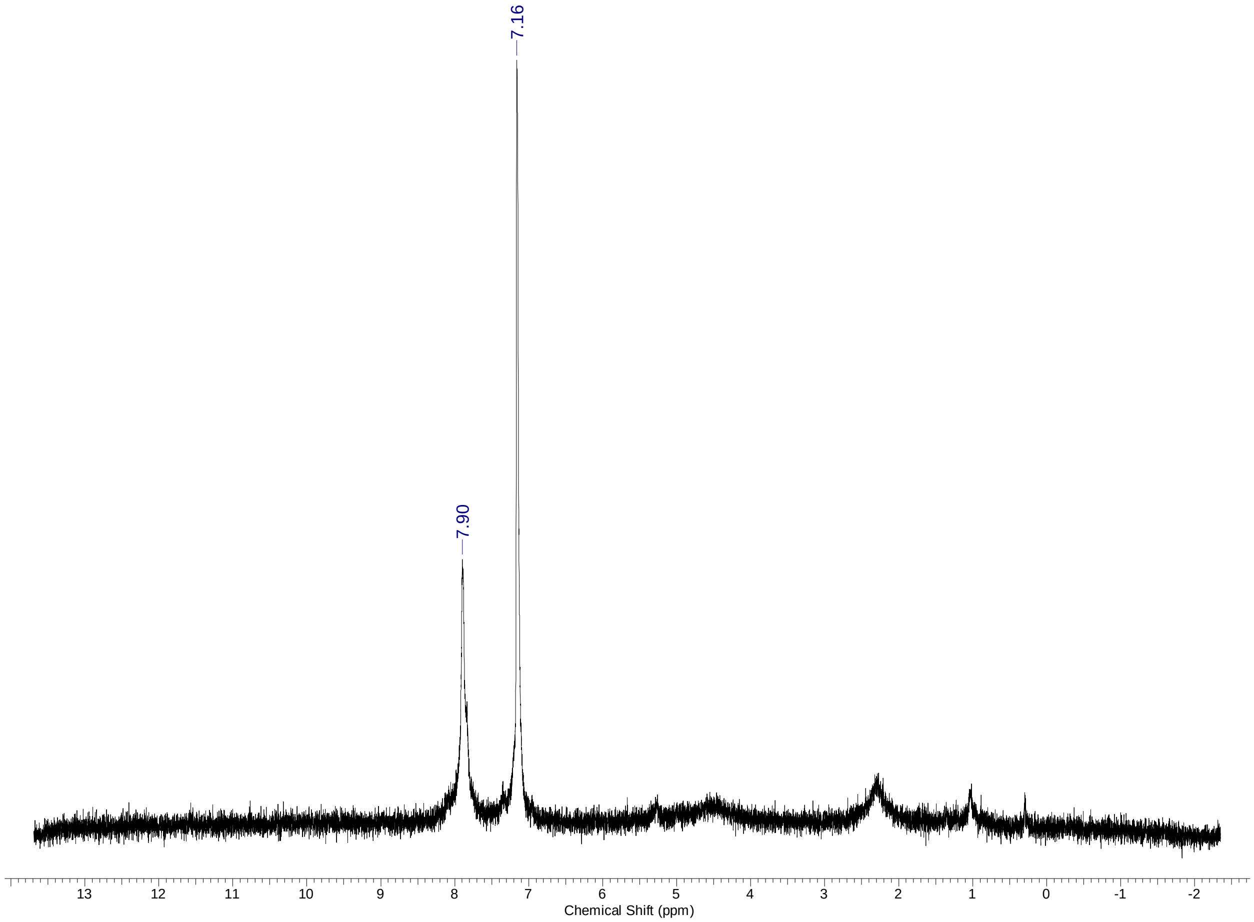

Figure S4. Characterization of **2** by Evans method (experiment #2).

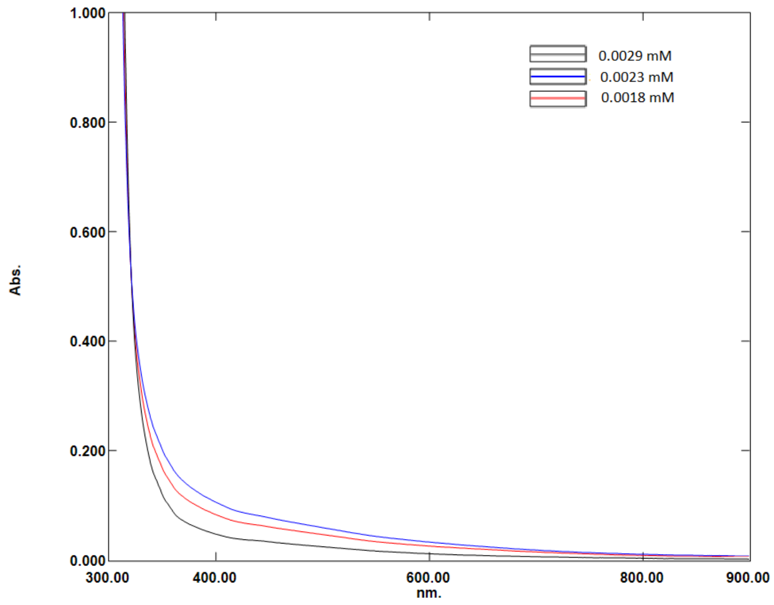

Figure S5. UV-vis spectrum of **2** at three different concentrations.

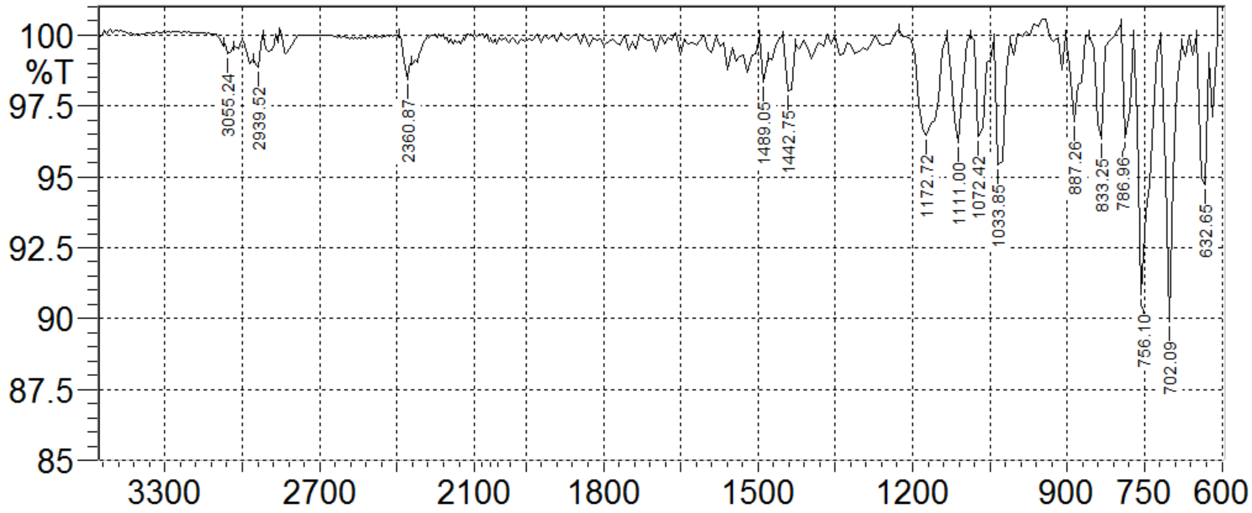

Figure S6. IR spectrum of **2**.

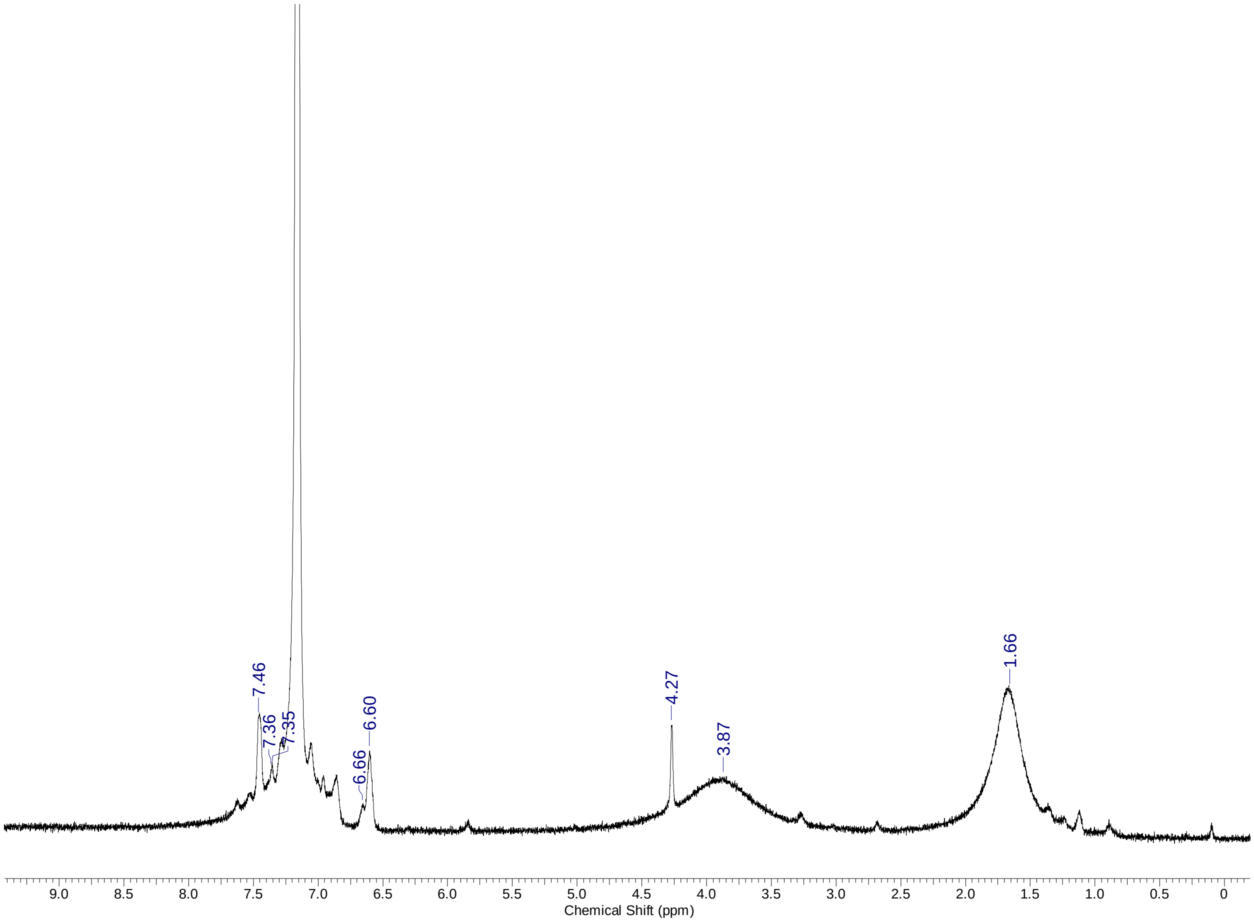

Figure S7. ^{1}H NMR of the reaction between **2** and PhINTs (in the absence of styrene). Peaks at 1.66 and 3.87 ppm are broad THF signals and 4.27 ppm is CH_2Cl_2. Peaks at 6.60 ppm is and 7.46 ppm are PhI resonances.

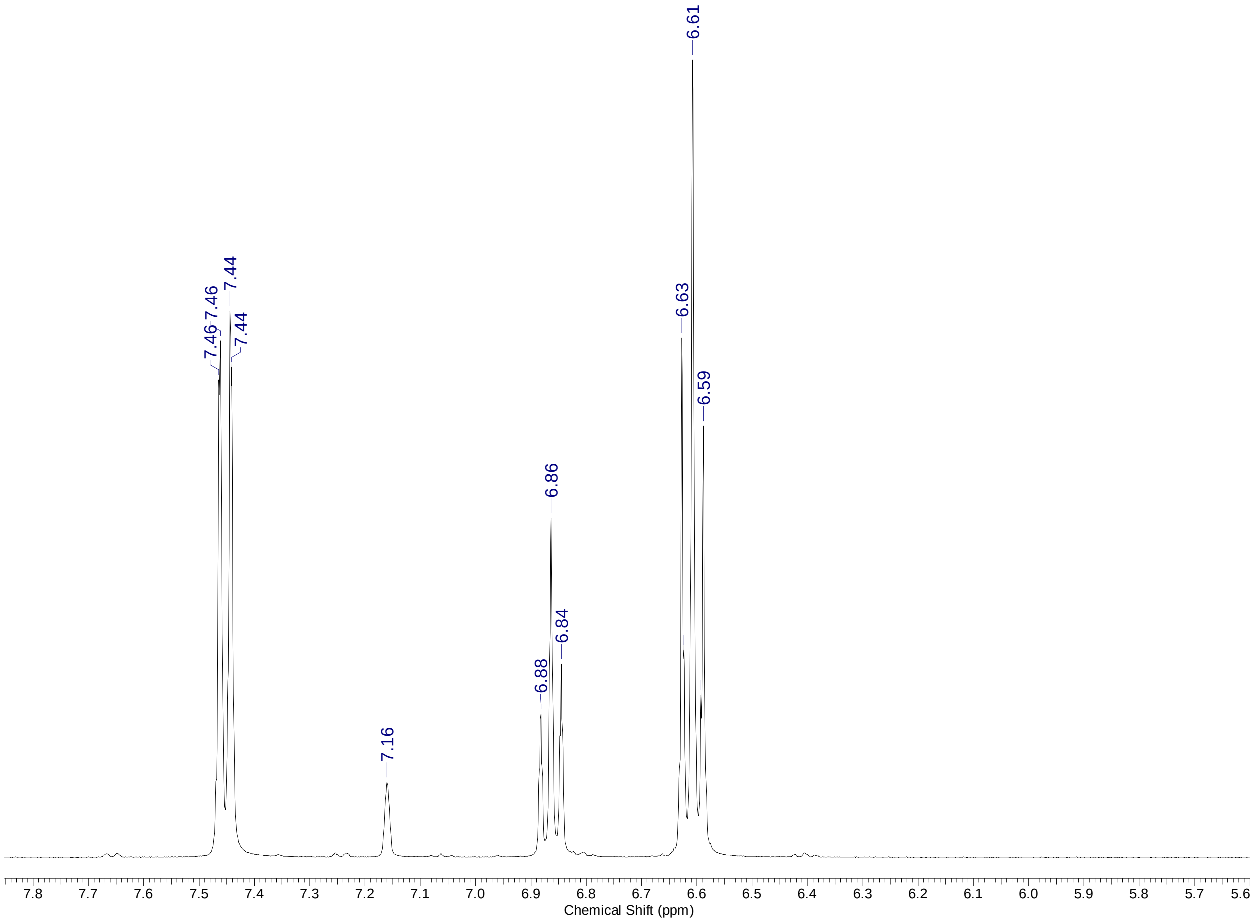

Figure S8. ^{1}H NMR spectrum of PhI in C_6D_6 (aromatic region).

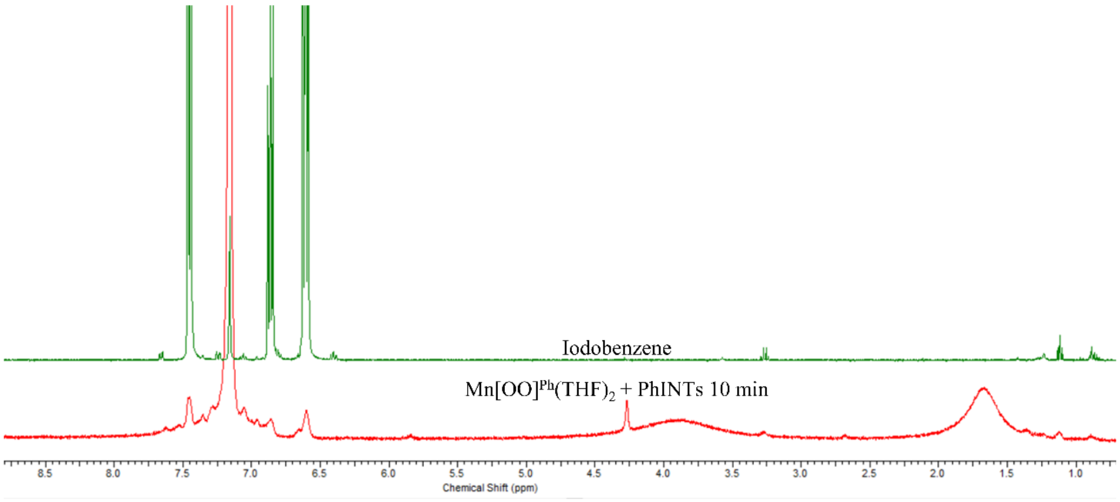

Figure S9. Stacked ^{1}H NMR spectra of PhI (top) and of {Mn[O-terphenyl-O]^{Ph}(THF)$_2$ + PhINTs} reaction (bottom) in C_6D_6.

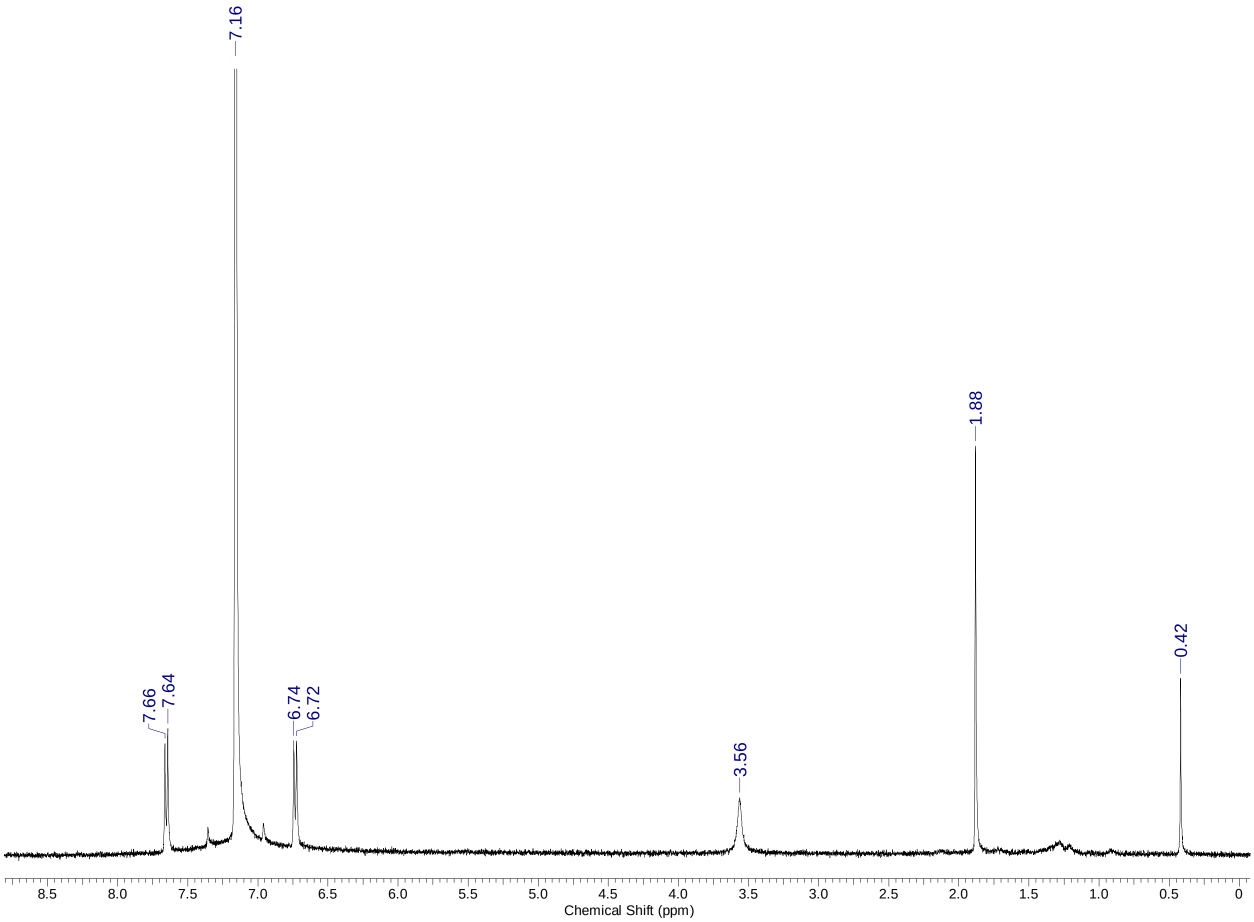

Figure S10. ^{1}H NMR of tosylamide in C_6D_6.

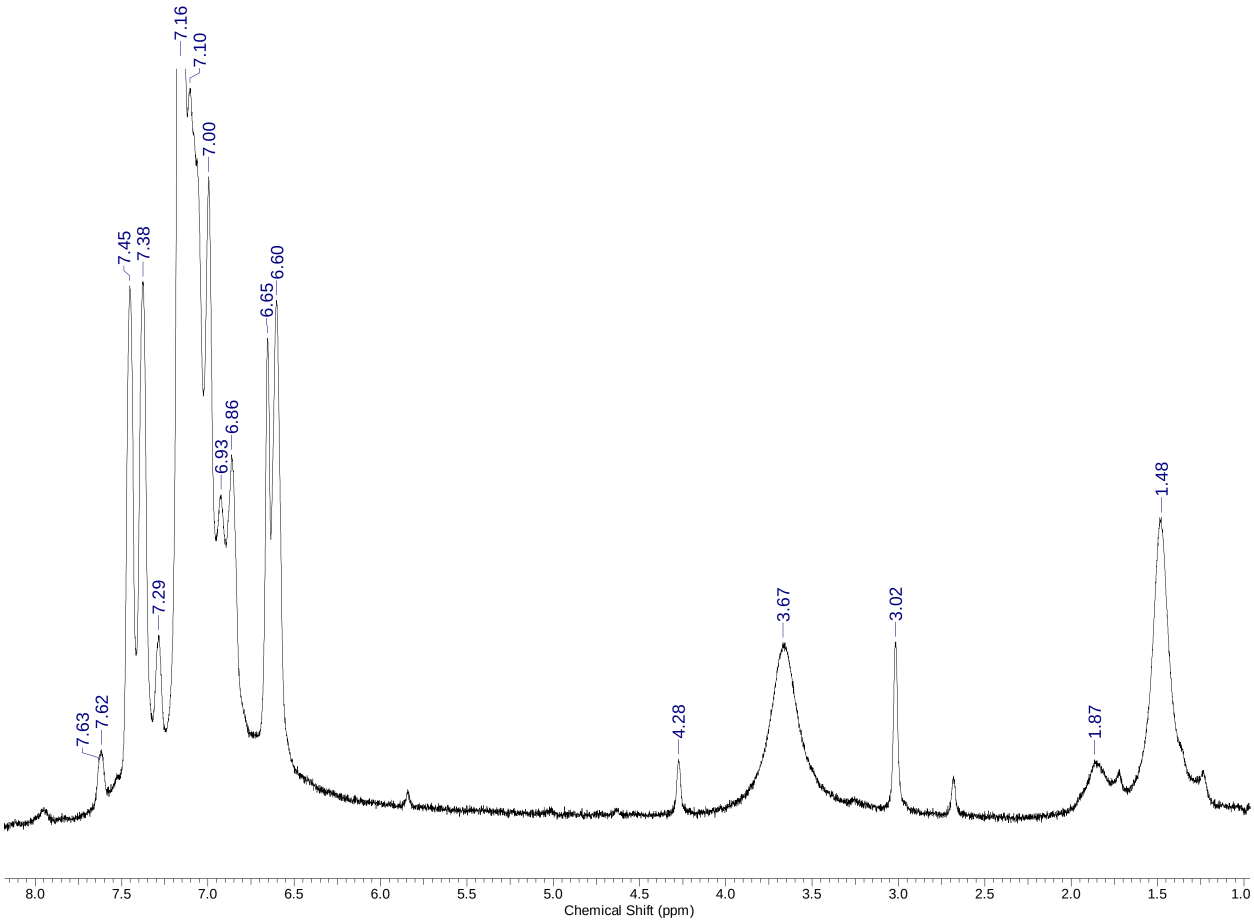

Figure S11. ^{1}H NMR spectrum of the reaction between Mn[O-terphenyl-O]^{Ph}(THF)$_2$ and PhINTs in C_6D_6 after 20 h. Peaks at 1.87 and 7.62-7.63 correspond with $TsNH_2$. Peaks at 6.60 ppm and 7.45 ppm correspond to iodobenzene.

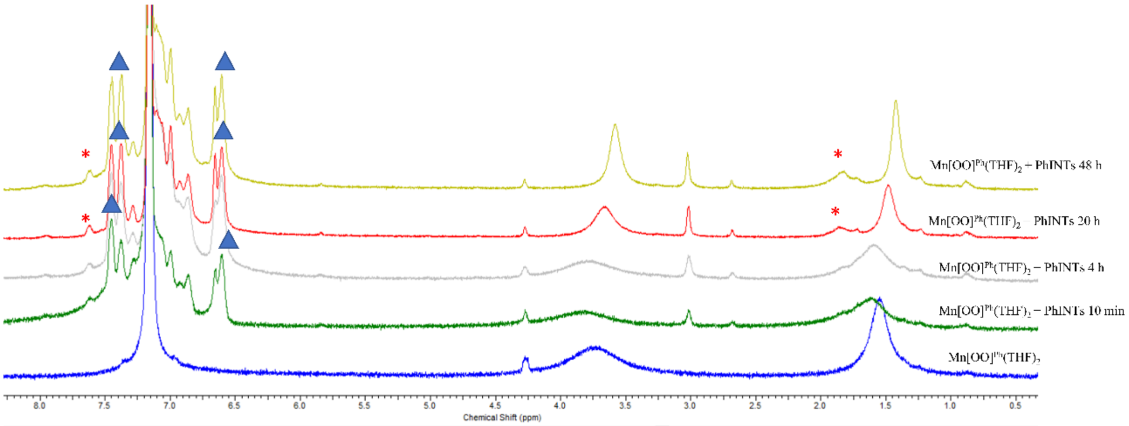

Figure S12. ^{1}H NMR spectra of the reaction of Mn[O-terphenyl-O]^{Ph}(THF)$_2$ and PhINTs in C_6D_6 at different times. Peaks at 1.87 and 7.62-7.63 (marked by *) correspond to $TsNH_2$.

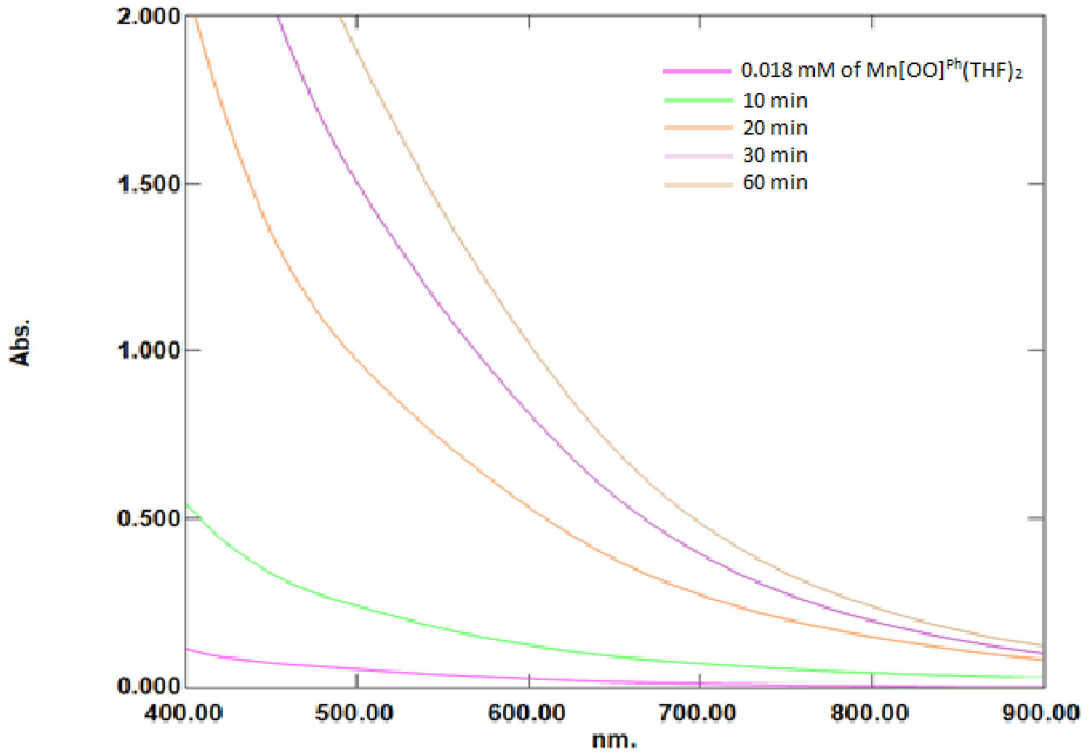

Figure S13. UV spectrum of 0.018 mM solution of Mn[O-terphenyl-O]^{Ph}(THF)$_2$ on addition of stoichiometric amounts of PhINTs at different time.

2. ^{1}H NMR characterization of the aziridine products.

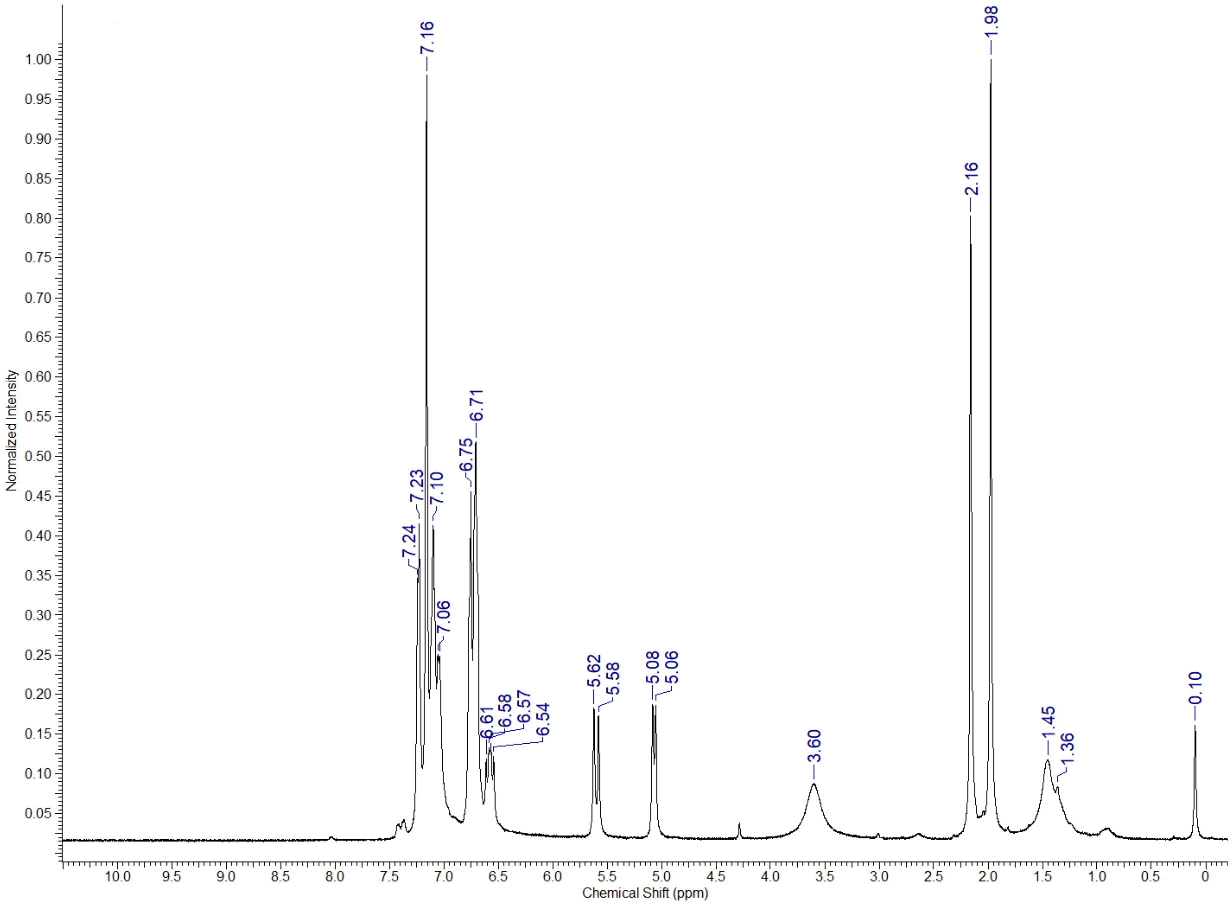

Figure S14. ^{1}H NMR demonstrating no formation of 2-phenyl-1-(p-tolyl)aziridine from the reaction of 4-azidotoluene with styrene and Fe[O-terphenyl-O]^{Ph}(THF)$_2$. The peak at 2.16 ppm corresponds to mesitylene standard.

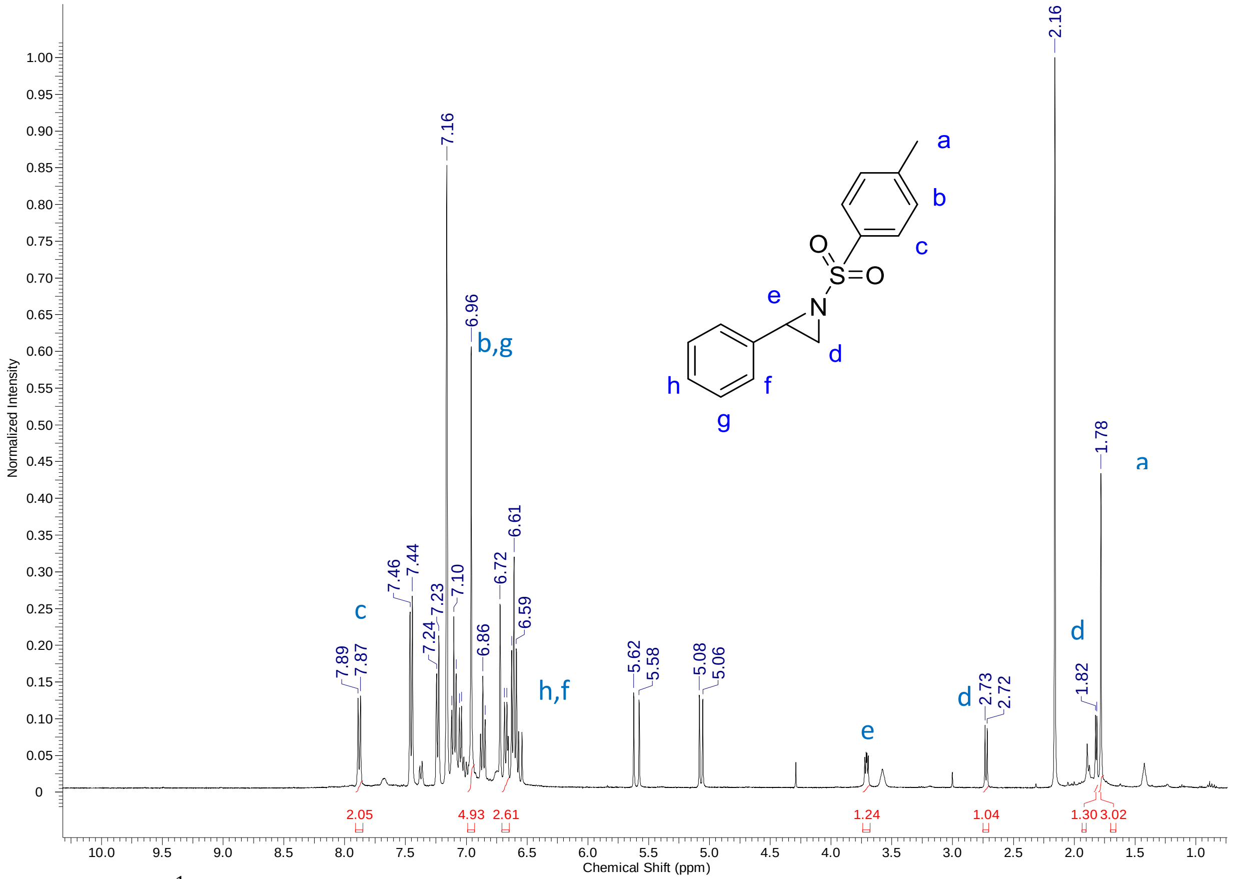

Figure S15. ^{1}H NMR demonstrating catalytic formation of 2-phenyl-1-tosylaziridine by Fe[O-terphenyl-O]^{Ph}(THF)$_2$ (**1**). The peak at 2.16 ppm corresponds to the mesitylene standard. 10.4 mg (0.012 mmol) of Fe[O-terphenyl-O]^{Ph}(THF)$_2$ was reacted with 47.8 mg (0.13 mmol) of PhINTs and 13.4 mg (0.13 mmol) of styrene.

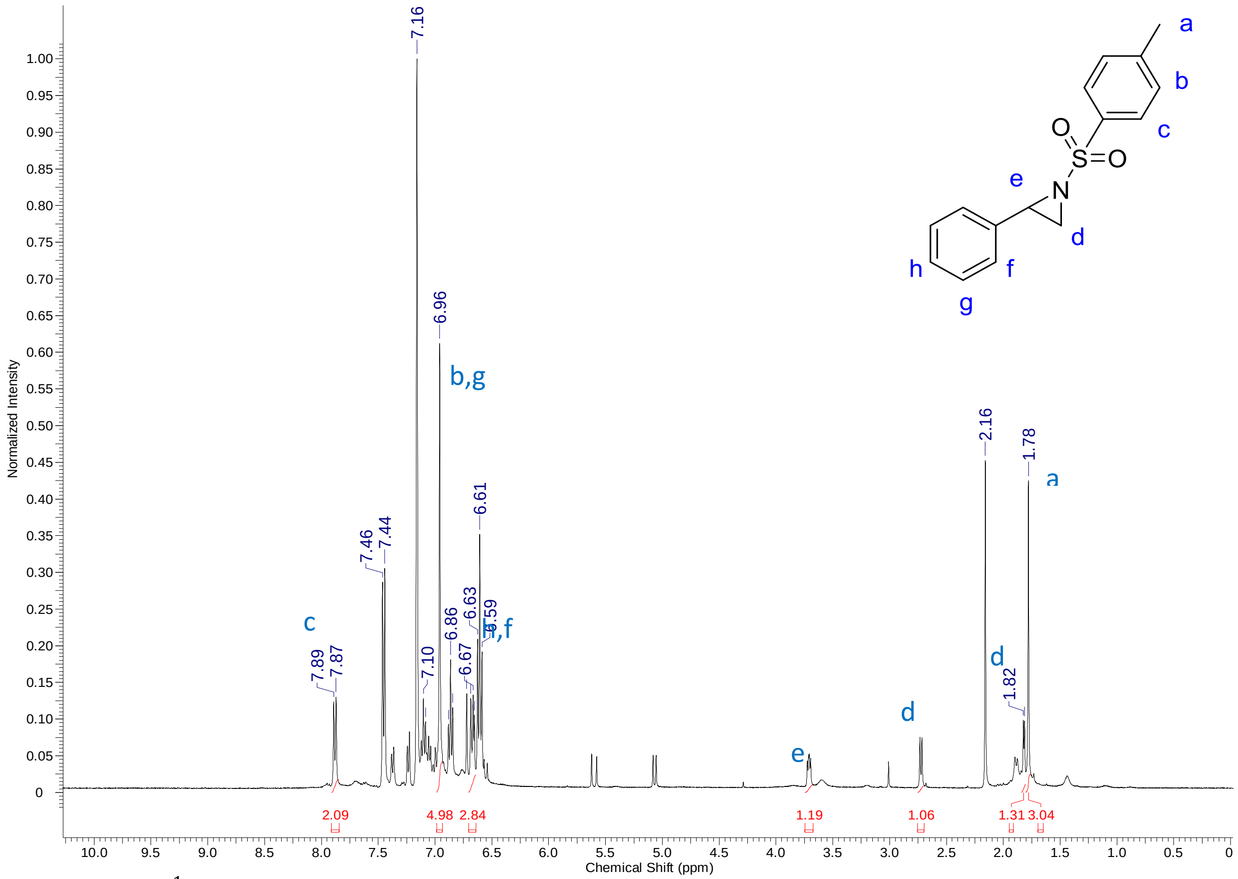

Figure S16. ^{1}H NMR demonstrating catalytic formation of 2-phenyl-1-tosylaziridine by Mn[O-terphenyl-O]^{Ph}(THF)$_2$ (**2**). The peak at 2.16 ppm corresponds to mesitylene standard. 10.3 mg (0.012 mmol) of Mn[O-terphenyl-O]^{Ph}(THF)$_2$ was reacted with 47.6 mg (0.13 mmol) of PhINTs and 13.2 mg (0.13 mmol) of styrene.

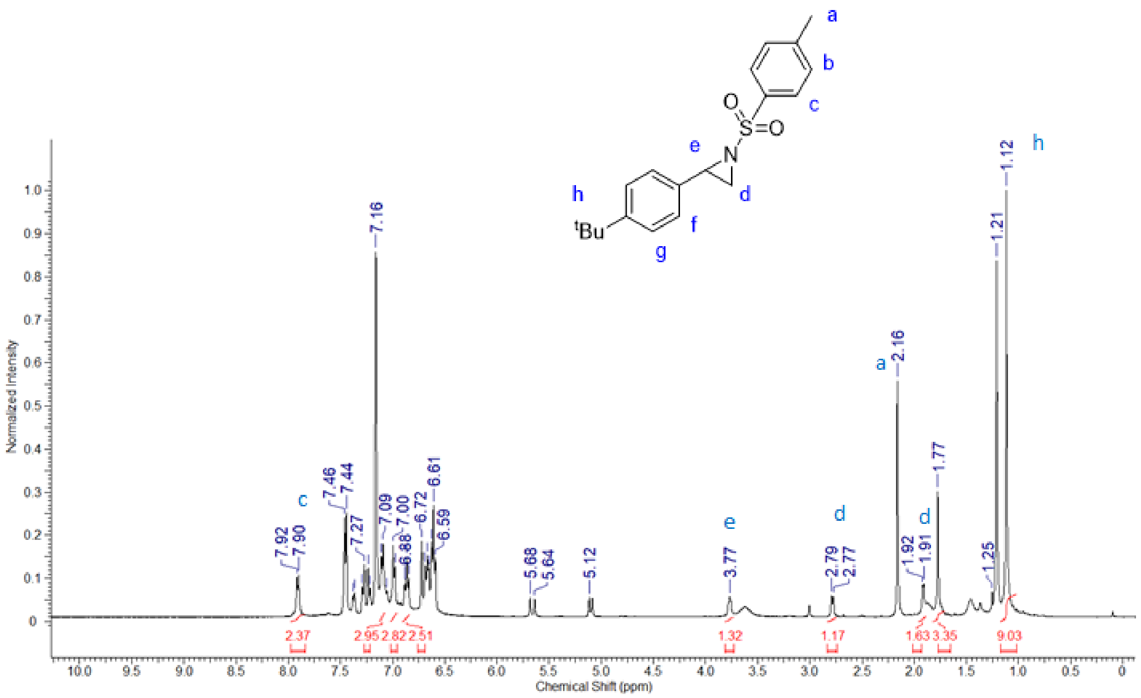

Figure S17. ^{1}H NMR demonstrating catalytic formation of 2-(4-(tert-butyl)phenyl)-1-tosylaziridine. 10.2 mg (0.012 mmol) of Mn[O-terphenyl-O]^{Ph}(THF)$_2$ was reacted with 47.8 mg (0.13 mmol) of PhINTs and 20.4 mg (0.13 mmol) of 4-*tert*-butylstyrene

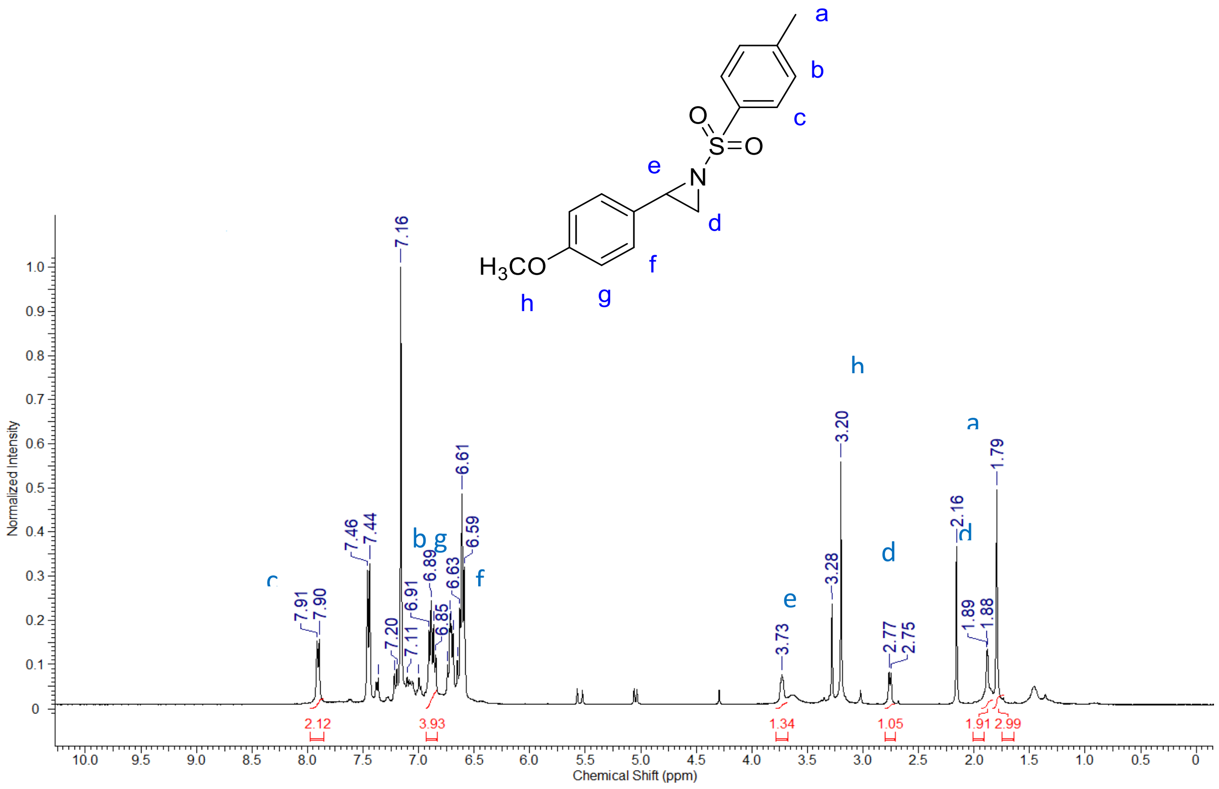

Figure S18. ^{1}H NMR demonstrating catalytic formation of 2-(4-methoxyphenyl)-1-tosylaziridine. 10.3 mg (0.012 mmol) of Mn[O-terphenyl-O]^{Ph}(THF)$_2$ was reacted with 47.3 mg (0.13 mmol) of PhINTs and 16.4 mg (0.13 mmol) of 4-methoxystyrene.

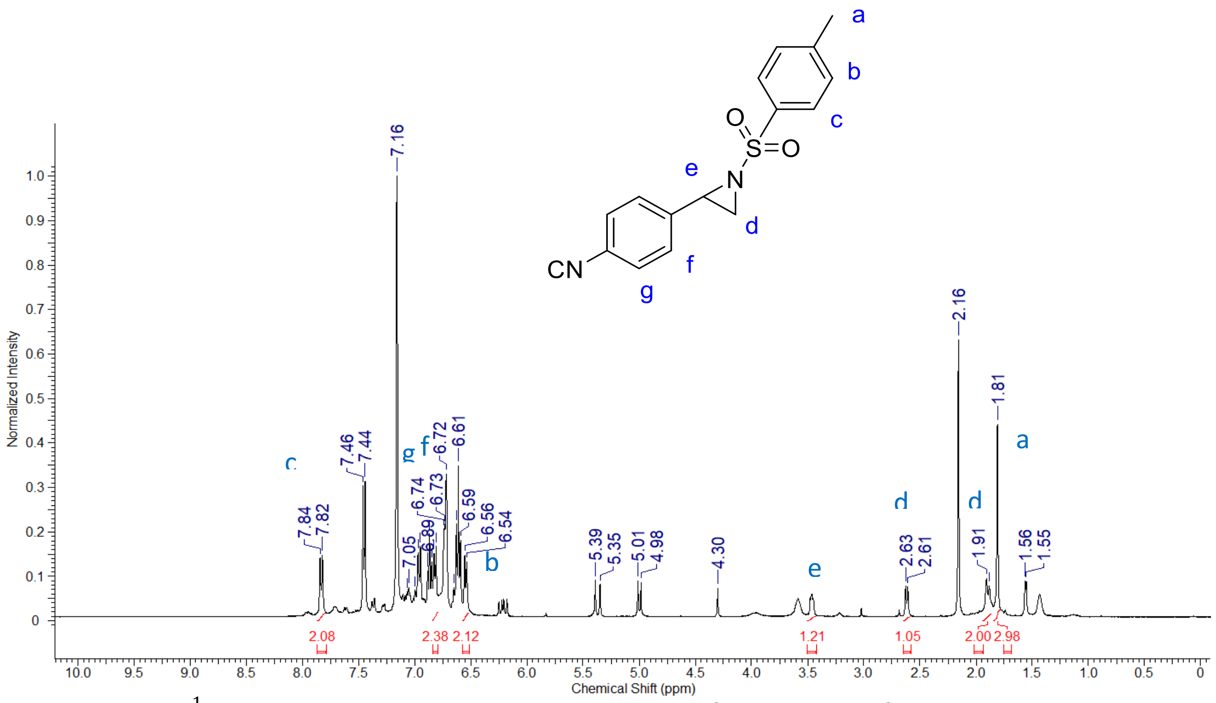

Figure S19. ^{1}H NMR demonstrating catalytic formation of 2-(4-isocyanophenyl)-1-tosylaziridine. 10.7 mg (0.012 mmol) of Mn[O-terphenyl-O]^{Ph}(THF)$_2$ was reacted with 47.3mg (0.13 mmol) of PhINTs and 17.8 mg (0.13 mmol) of 4-cyanostyrene.

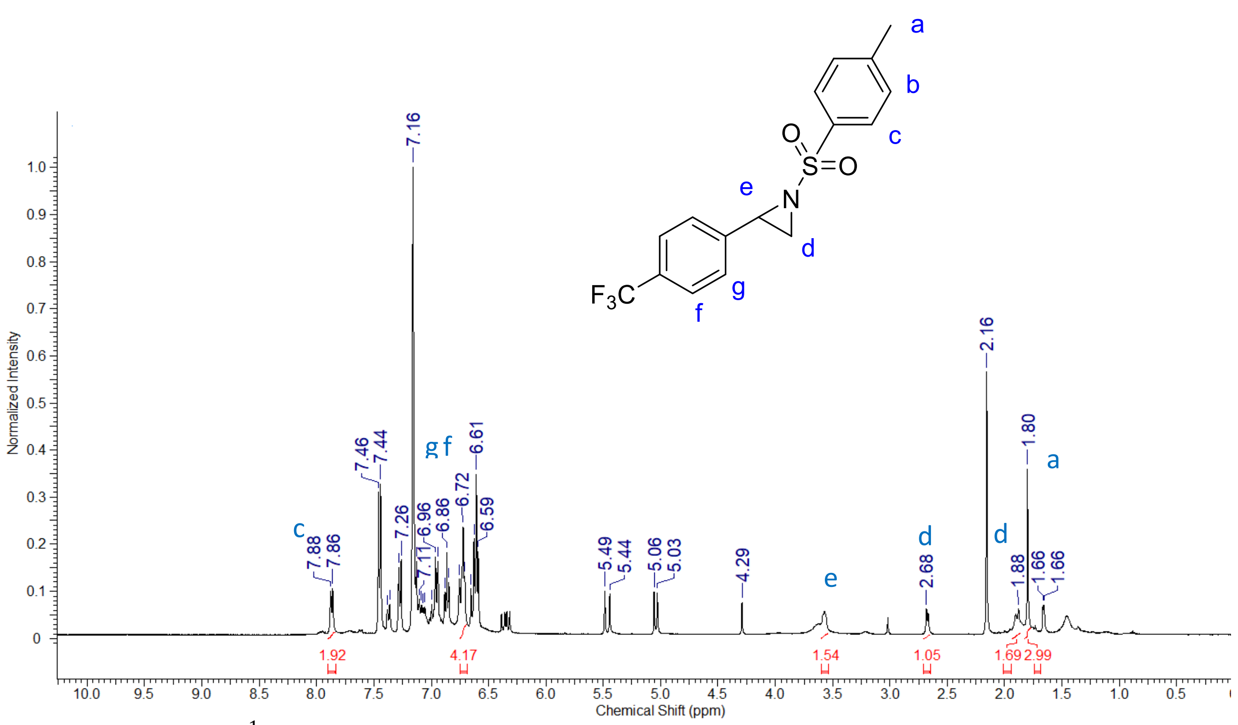

Figure S20. ^{1}H NMR demonstrating catalytic formation of 1-tosyl-2-(4-(trifluoromethyl)phenyl)aziridine. 10.5 mg (0.012 mmol) of Mn[O-terphenyl-O]^{Ph}(THF)$_2$ was reacted with 47.8 mg (0.13 mmol) of PhINTs and 23.3 mg (0.13 mmol) of 4-trifluoromethyl styrene.

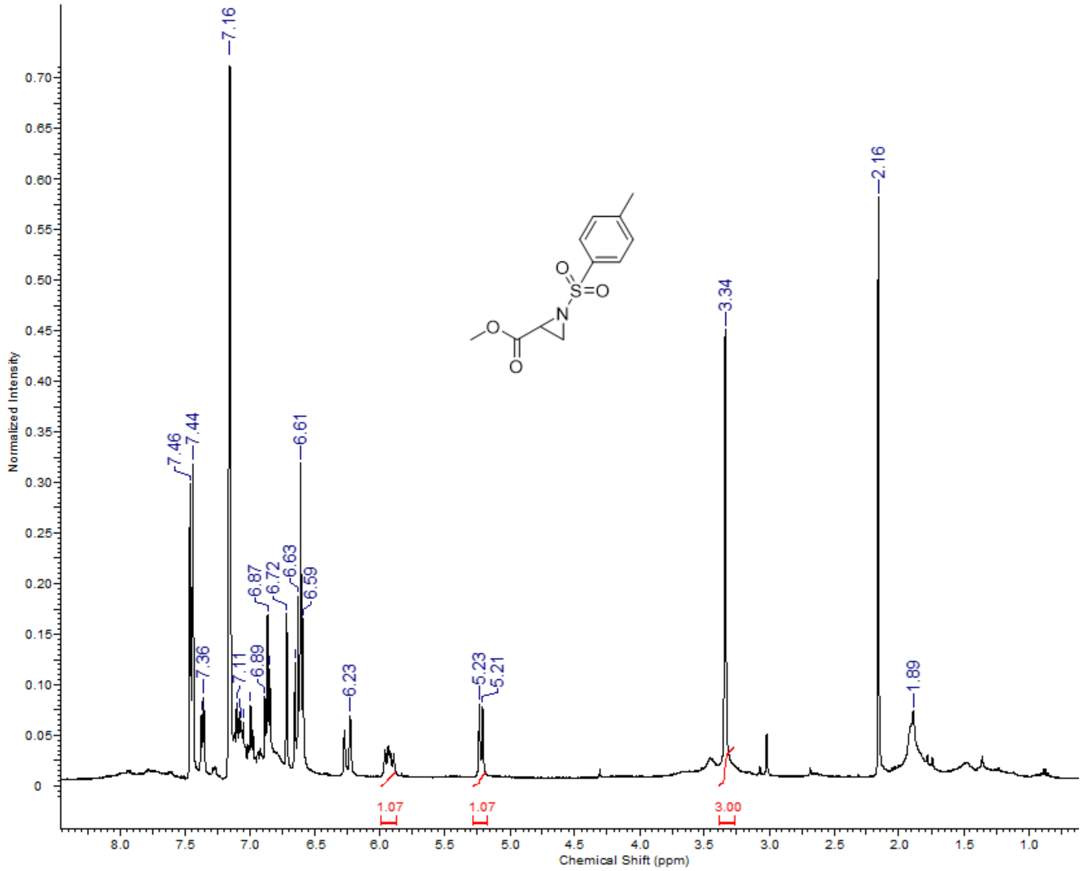

Figure S21. ^{1}H NMR demonstrating no formation of methyl 1-tosylaziridine-2-carboxylate. 10.1 mg (0.012 mmol) of Mn[O-terphenyl-O]^{Ph}(THF)$_2$ was reacted with 47.6 mg (0.13 mmol) of PhINTs and 11.1 mg (0.13 mmol) of methyl acrylate.

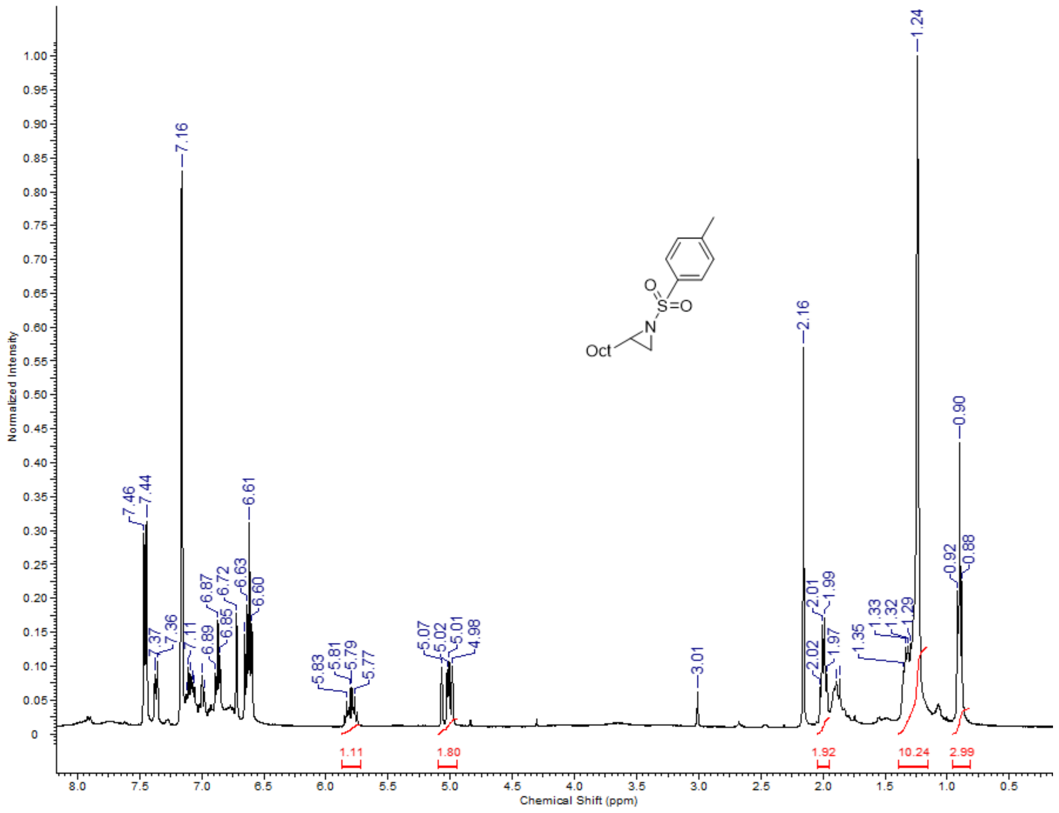

Figure S22. ^{1}H NMR demonstrating no formation of 1-(1-tosylaziridin-2-yl)octan-1-one. 10.4 mg (0.012 mmol) of Mn[O-terphenyl-O]^{Ph}(THF)$_2$ was reacted with 47.8 mg (0.13 mmol) of PhINTs and 18.4 mg (0.13 mmol) of 1-decene.

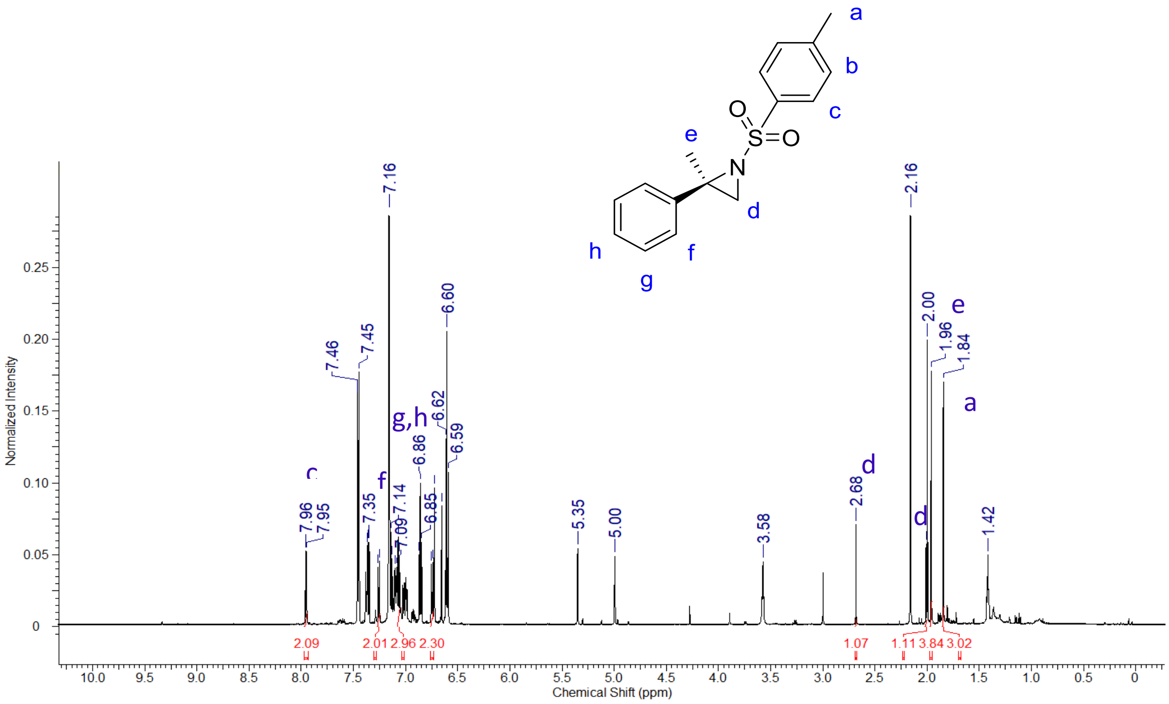

Figure S23. ^{1}H NMR demonstrating catalytic formation of 2-methyl-2-phenyl-1-tosylaziridine. 10.1 mg (0.012 mmol) of Mn[O-terphenyl-O]^{Ph}(THF)$_2$ was reacted with 47.6 mg (0.13 mmol) of PhINTs and 15.1 mg (0.13 mmol) of α-methylstyrene.

3. GC-MS spectra

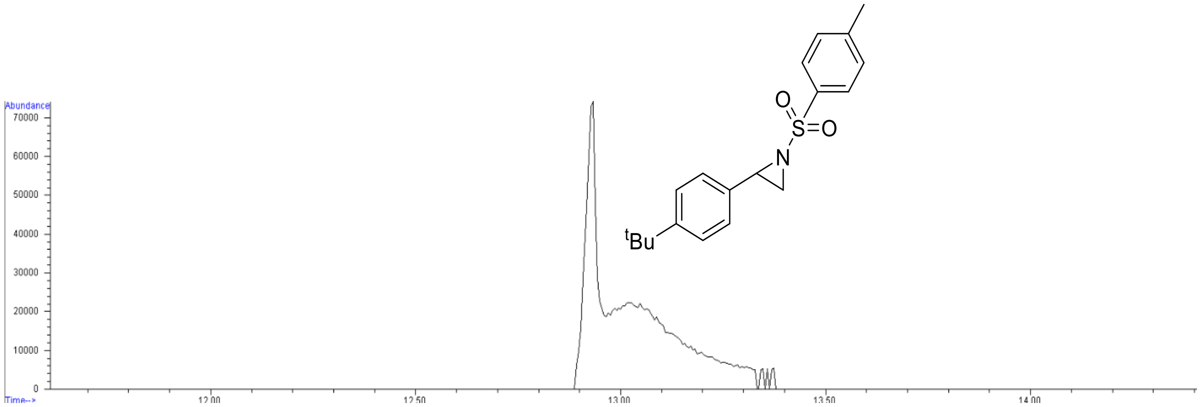

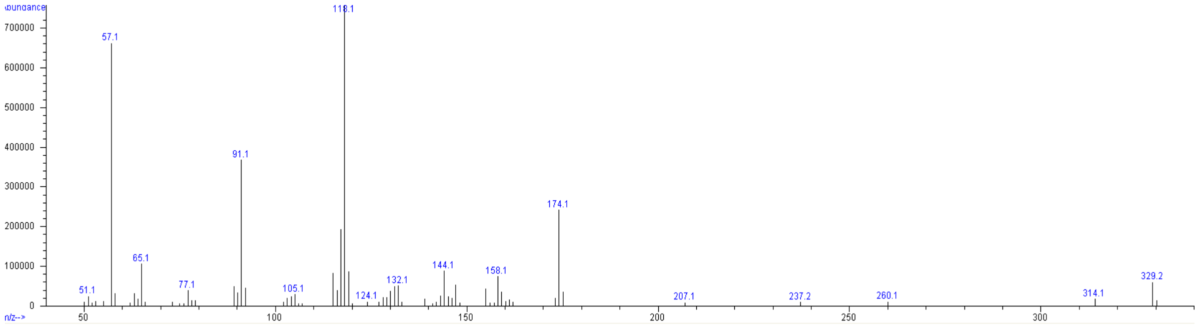

Figure S24. GC-MS of 2-(4-(tert-butyl)phenyl)-1-tosylaziridine.

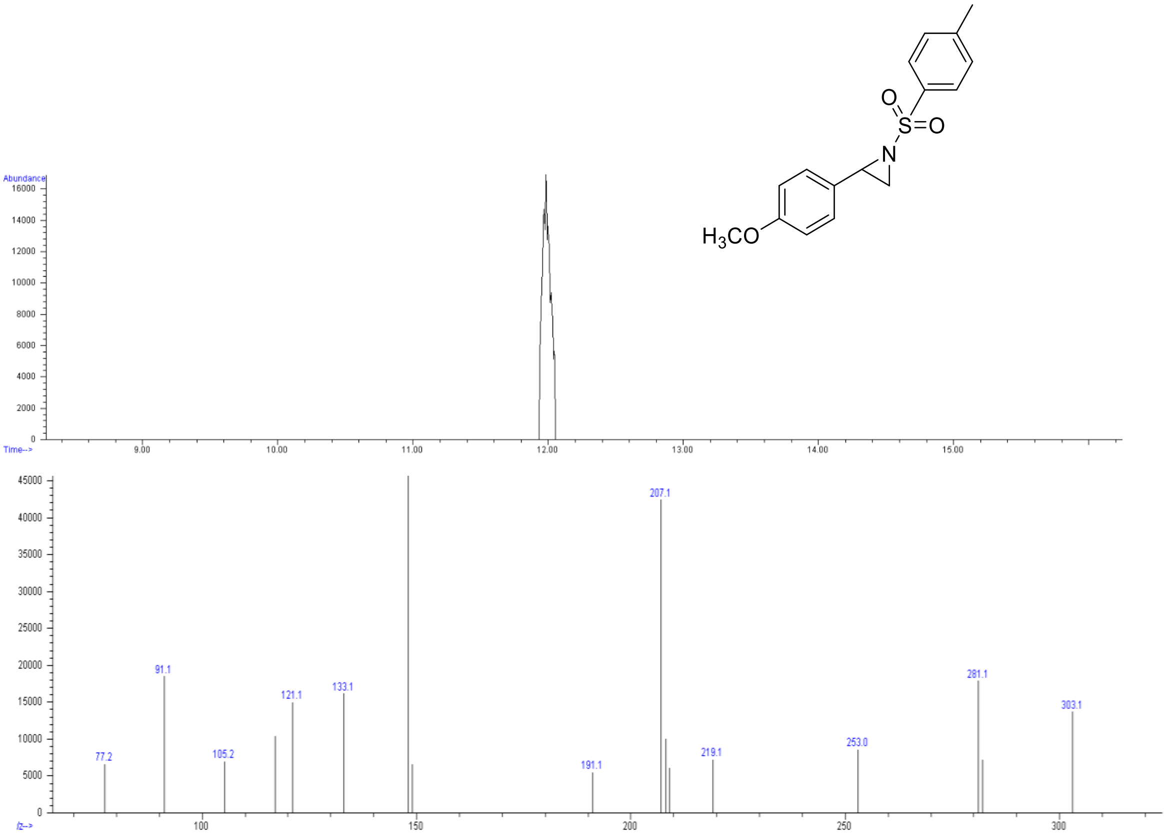

Figure S25. GC-MS of 2-(4-methoxyphenyl)-1-tosylaziridine.

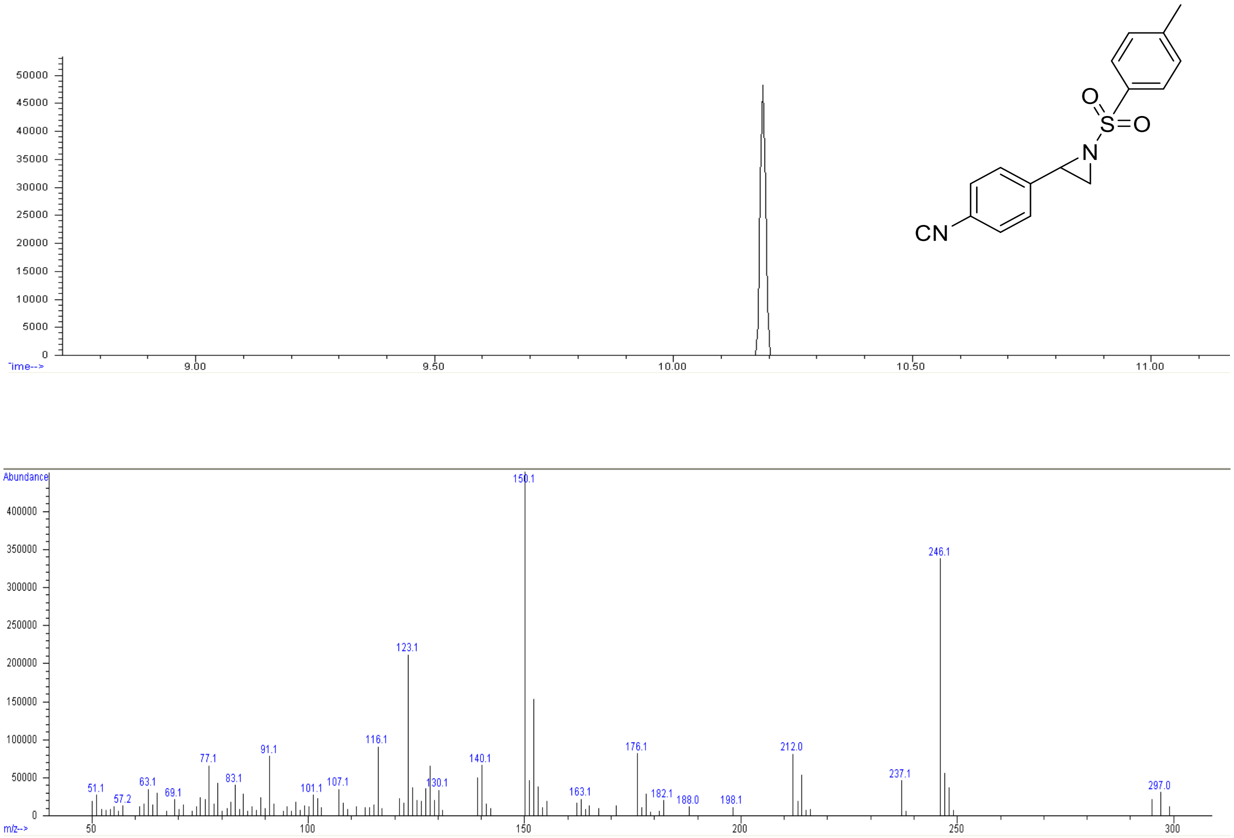

Figure S26. GC-MS of 2- (4-isocyanophenyl)-1-tosylaziridine.

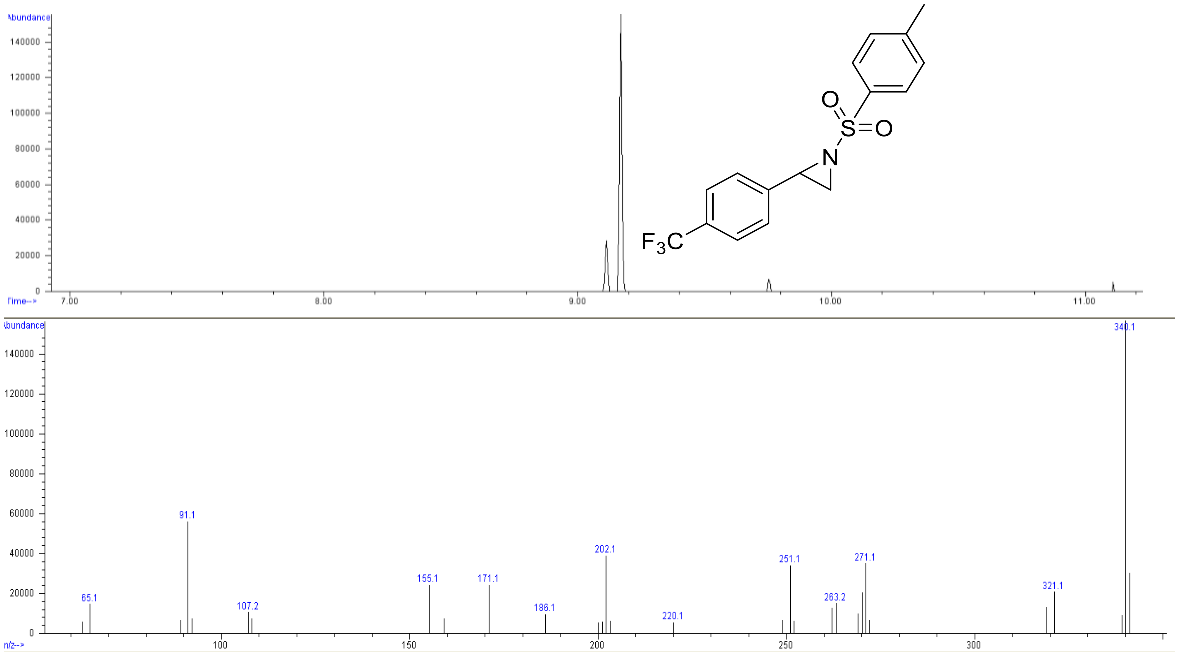

Figure S27. GC-MS of 1-tosyl-2-(4-(trifluoromethyl)phenyl)aziridine.

4. Computational Data

All intermediates along the reaction pathway were probed as doublets, quartets, and sextets to allow for various combinations of Mn^{II}/NR^{0} to Mn^{IV}/NR^{2-}. Doublets were always higher in energy and are therefore only reported for the iminoiodinane/imido complexes. Multiple conformers of the tosyl substituent were probed (**Figure S28**) but the isomer with p-methyltoluene protruding into solution, and oxygen close to coordinating to Mn, was found to be lowest in energy for most structures (iso1). Different regio and stereoisomers were considered for the styrene added intermediate **C**. Unsurprisingly, the radical intermediate is significantly stabilized when localized on the secondary vs. primary carbon so we only present the former. We observe a stereochemical preference but that is due to our modeling only considering one possible conformation of the ligand backbone; in solution one expects a 50:50 mixture of these coordinations and therefore no stereoselectivity for one aziridine over another. Energies are summarized in Table S2, frequencies for optimized structures in Table S3, and all optimized structures can be found in the xyz file available in the Supporting Information.

Figure S28. Illustration of optimized structures of iso1 vs. iso2 for the iminoiodinane intermediate **A** that demonstrates the significant N-I activation.

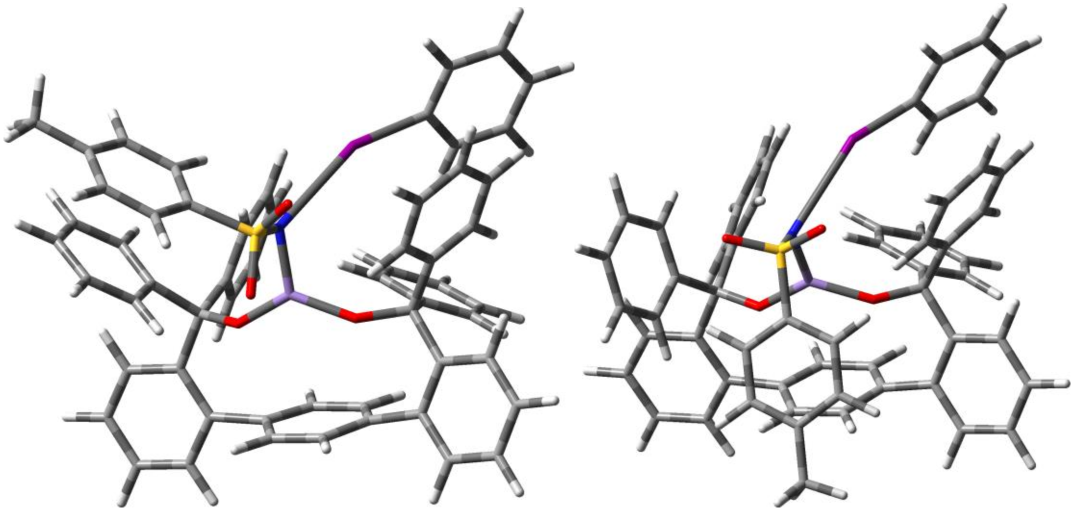

Table S1. Bond order analysis of iminoiodinane adducts (intermediate **A**).

Structure	Spin State	Mayer	Wiberg	Bond Length (Å)
TsNIPh	Singlet	1.05	1.27	2.059
iso1	Sextet	0.08	0.11	2.955
iso2	Sextet	<0.05	0.05	3.173
iso1	Quartet	0.08	0.11	2.937
iso2	Quartet	0.05	0.07	3.096
iso1	Doublet	0.24	0.32	2.638
iso2	Doublet	0.10	0.12	3.001

Figure S29. Spin density isosurface plot (iso = 0.002 au) for the quartet iminoiodinane intermediate **A** (left) compared to the imido structure **B** (right).

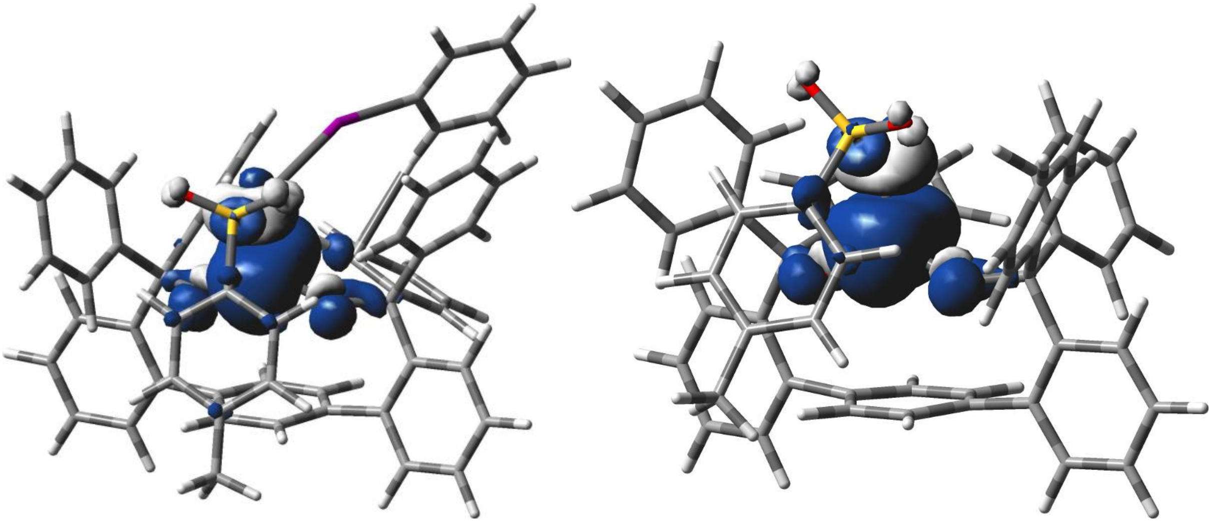

Figure S30. Spin density isosurface plot (iso = 0.002 au) for the sextet radical intermediate **C**.

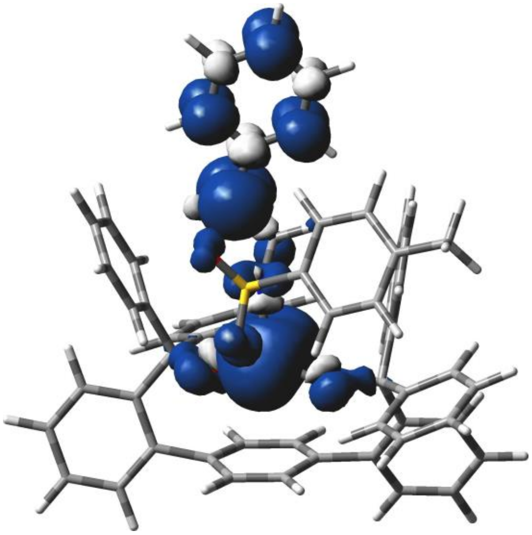

Table S2. Energies (E_h) of optimized structures.

Structure	Spin State	E_{DZ}	H(gas)	G(gas)	E_{TZ}	CalcID
Mn[O-terphenyl-O]^{Ph}(THF)$_2$	sextet	-3461.041137	-3460.148207	-3460.290597	-3463.563386	TMn001
Mn[O-terphenyl-O]^{Ph}	sextet	-2996.384408	-2995.735497	-2995.847667	-2997.846798	TMn011
THF	singlet	-232.286460	-232.167475	-232.202388	-232.556602	THF
styrene	singlet	-309.443171	-309.305840	-309.345084	-309.789589	STYRENE
iminoiodinane	singlet	-1403.138896	-1402.904865	-1402.975783	-1404.046199	NI001
phenyl iodide	singlet	-529.331578	-529.237124	-529.275943	-529.556431	PhI
Int **A**, iso1	doublet	-4399.649153	-4398.762715	-4398.917982	-4402.474009	MN053
Int **A**, iso2	doublet	-4399.631578	-4398.745641	-4398.899749	-4402.492884	MN053r
Int **A**, iso1	quartet	-4399.662672	-4398.776403	-4398.931406	-4402.506212	MN052
Int **A**, iso2	quartet	-4399.659462	-4398.773032	-4398.928220	-4402.506047	MN052r
Int **A**, iso1	sextet	-4399.631843	-4398.746679	-4398.902621	-4402.487853	MN051
Int **A**, iso2	sextet	-4399.640583	-4398.753818	-4398.905761	-4402.472309	MN051r
Int **B**, iso1	doublet	-3870.277419	-3869.486997	-3869.621762	-3872.897459	MN001c
Int **B**, iso2	doublet	-3870.273091	-3869.483782	-3869.618042	-3872.893771	MN007
Int **B**, iso1	quartet	-3870.299026	-3869.509280	-3869.646668	-3872.930194	MN003
Int **B**, iso2	quartet	-3870.299026	-3869.509280	-3869.646664	-3872.930199	MN009
Int **B**, iso1	sextet	-3870.263480	-3869.474234	-3869.613205	-3872.913423	MN005
Int **B**, iso2	sextet	-3870.265795	-3869.476685	-3869.612464	-3872.912177	MN010
Int **C**, iso1a	quartet	-4179.779563	-4178.849613	-4179.002265	-4182.764078	MN070
Int **C**, iso1b	quartet	-4179.783999	-4178.853949	-4179.003699	-4182.767982	MN071
Int **C**, iso2a	quartet	-4179.773237	-4178.843269	-4178.992751	-4182.753899	MN074
Int **C**, iso2b	quartet	-4179.776399	-4178.845745	-4179.000252	-4182.759573	MN075
Int **C**, iso1a	sextet	-4179.780050	-4178.850119	-4179.002640	-4182.763915	MN081
Int **C**, iso1b	sextet	-4179.784474	-4178.854482	-4179.004823	-4182.768680	MN082
Int **C**, iso2a	sextet	-4179.774368	-4178.843415	-4178.996950	-4182.756144	MN083
Int **C**, iso2b	sextet	-4179.776853	-4178.846215	-4179.001427	-4182.760079	MN084
Int **D**, iso1a	quartet	-4179.806978	-4178.877649	-4179.027699	-4182.743518	MN096
Int **D**, iso1b	quartet	-4179.772736	-4178.842644	-4178.995269	-4182.731312	MN097a
Int **D**, iso2a	quartet	-4179.778261	-4178.847567	-4179.000951	-4182.734473	MN098a
Int **D**, iso2b	quartet	-4179.783594	-4178.852476	-4179.006539	-4182.736723	MN101a
Int **D**, iso1a	sextet	-4179.783719	-4178.853933	-4179.002466	-4182.781443	MN102a
Int **D**, iso1b	sextet	-4179.785494	-4178.854303	-4179.009623	-4182.782628	MN103
Int **D**, iso2a	sextet	-4179.787670	-4178.855546	-4179.011336	-4182.782685	MN104a
Int **D**, iso2b	sextet	-4179.802928	-4178.871272	-4179.019191	-4182.793530	MN105
TS **B**-**C**-1a	quartet	-4179.757455	-4178.828611	-4178.982555	-4182.733288	MN086c
TS **B**-**C**-1b	quartet	-4179.758117	-4178.830076	-4178.981401	-4182.733707	MN087c
TS **B**-**C**-2a	quartet	-4179.757455	-4178.828611	-4178.982544	-4182.708880	MN090
TS **B**-**C**-2b	quartet	Not found				
TS **C**-**D**-1a	sextet	-4179.762197	-4178.832815	-4178.988421	-4182.755034	MN107
TS **C**-**D**-1b	sextet	-4179.769756	-4178.839943	-4178.995690	-4182.761341	MN108
TS **C**-**D**-2a	sextet	-4179.762197	-4178.832815	-4178.988416	-4182.755034	MN109
TS **C**-**D**-2b	sextet	Not found				

Table S3. Harmonic frequencies (cm^{-1}) for all optimized species.

Mn[O-terphenyl-O]^{Ph}$(THF)_2$ S=5/2

9.2000	20.9668	22.0465
28.8956	34.7278	40.2600
46.0689	46.3864	49.4737
54.8332	55.1763	58.1343
58.9869	62.5056	66.0156
71.1429	73.0233	77.8753
79.4771	84.2058	88.9656
95.5837	102.2237	104.5877
109.1053	111.3691	114.9244
127.0872	128.5950	153.3502
160.2724	170.7235	176.4309
185.3630	189.9206	204.3726
206.5484	211.2393	215.3347
224.4755	228.0759	248.6428
253.7147	263.3846	264.7686
268.6375	277.1827	286.5812
292.2693	313.3784	320.7744
323.5420	328.2481	349.6937
372.5212	377.4955	391.2472
399.1986	400.6490	401.1857
406.2680	409.3219	425.0467
464.5212	468.6195	485.8977
503.0494	507.9839	518.3112
521.4083	537.8215	551.8968
559.6157	576.1525	611.6846
613.1803	613.2791	613.8400
614.4043	622.8895	627.3468
630.0558	634.2371	635.0661
635.2081	636.9559	641.9938
653.1882	674.6935	688.1141
696.3562	698.9776	700.8635
705.0728	708.1509	722.7722
728.9069	746.1303	748.3091
750.8386	757.3808	761.1339
770.3162	775.1270	776.7093
782.1179	787.8028	819.1159
824.9183	829.6956	831.4784
832.5522	834.5272	836.7346
843.6649	847.5683	864.0223
866.0901	873.0301	882.7979
886.9638	891.5778	894.7630
896.2489	901.9898	905.1941
913.4030	918.3063	926.1638
928.8037	930.7070	931.2831
935.8382	936.4896	941.2528
945.4949	946.4260	957.8841
958.2452	958.7610	961.7380
968.3556	975.4496	975.5518
977.6263	978.5585	981.1603
984.2706	986.5347	987.6526

988.1988	988.4934	988.9492
1003.4239	1017.6000	1023.4052
1024.3634	1025.1462	1026.2600
1027.0989	1030.1707	1036.3319
1039.0028	1047.4073	1050.3893
1064.7354	1065.8590	1069.7405
1072.1077	1089.4604	1094.3597
1097.3175	1097.5903	1100.9604
1119.1332	1122.4945	1131.6737
1131.7547	1132.8231	1134.8272
1138.1416	1138.9363	1145.1995
1146.6656	1147.4900	1151.6046
1153.9054	1157.3247	1158.8088
1159.6812	1160.9894	1170.3701
1170.8772	1171.9200	1180.2159
1184.9723	1186.0064	1203.8237
1209.7006	1211.2140	1221.1308
1239.7136	1240.4708	1257.8323
1261.6530	1262.1111	1263.6446
1275.6565	1277.6978	1280.0683
1280.7188	1283.8674	1287.9446
1290.8066	1311.6389	1312.6030
1323.6840	1332.5594	1338.1677
1346.4527	1350.2850	1366.3671
1367.8341	1368.4209	1369.6903
1398.5325	1403.1158	1408.9522
1413.5901	1418.9096	1421.7829
1424.2499	1424.8756	1427.8647
1429.3397	1430.8801	1430.9689
1444.3922	1448.9011	1457.5258
1459.5634	1468.6922	1469.6911
1469.9678	1470.3900	1474.3751
1507.4997	1550.1446	1575.2682
1577.8910	1588.8529	1589.4902
1591.4390	1591.7524	1602.9643
1604.6110	1605.2615	1605.3766
1609.0173	1609.1301	1614.8856
2933.3591	2938.0428	2942.8517
2977.6595	2986.5322	2995.4564
2998.2755	3006.4041	3037.3766
3046.8146	3049.5727	3052.5867
3056.9400	3061.8463	3066.6451
3083.8369	3089.7413	3090.1000
3090.2305	3090.7835	3092.7064
3093.5736	3094.8393	3099.0998
3099.7787	3100.0516	3101.3055
3101.9744	3102.5473	3105.9149
3110.3040	3112.0153	3113.3008
3114.0153	3114.2078	3114.3678
3119.9357	3120.0686	3121.1953
3121.6421	3121.8770	3122.4242
3123.5601	3124.5196	3125.8869
3128.6512	3134.9460	3149.6856

Mn[O-terphenyl-O]^{Ph} S=5/2

-7.8539	20.2536	24.4368
29.9701	33.2058	38.0719
44.0095	48.0270	52.1166
59.8226	60.1274	66.7479
72.9954	78.2408	82.9870
89.8892	91.8971	107.4082
108.4923	134.9757	170.2403
174.8145	199.7497	203.4942
214.1667	227.2484	235.6649
249.2635	253.4672	262.4283
266.5985	270.5191	293.0300
295.1732	307.9139	317.3698
323.0906	347.0099	364.1691
387.7918	398.9407	401.1819
402.0115	405.4042	406.6632
408.5512	427.9750	461.7181
481.5882	497.8731	502.4823
506.5800	520.5164	533.7534
542.0828	552.8436	575.4267
610.7104	612.6329	612.8871
613.3176	615.0090	623.7452
628.7578	632.0748	634.1816
635.8795	650.2137	660.5221
693.5114	699.1476	701.3076
706.0975	708.6870	709.3701
718.0681	728.0705	747.8249
748.9101	751.2795	757.6440
760.5192	765.0138	769.0754
780.9009	784.1290	826.0619
833.0210	833.8381	835.9014
836.7893	839.5813	864.4821
866.9123	885.0579	889.2579
900.9948	902.0390	924.8862
925.9020	929.5427	931.8286
941.0832	946.7395	947.0772
956.3275	958.2940	961.3376
962.3243	963.1432	978.6429
979.2523	980.6473	981.1812
981.8580	982.1496	986.7867
987.2825	988.3413	988.8942
990.4233	1001.1016	1020.5330
1022.5270	1023.7218	1023.8885
1024.7832	1045.1772	1051.3694
1056.2735	1061.8543	1065.6853
1066.9220	1081.3838	1087.4886
1095.9055	1097.5667	1104.0150
1130.1510	1132.3858	1132.6584
1134.9295	1139.3583	1140.9878
1144.0292	1145.8975	1148.1404
1149.7408	1154.3970	1155.1045
1155.9075	1171.1530	1174.6077
1181.9561	1186.5620	1241.8481
1243.8184	1260.7639	1269.9508
1275.5906	1278.8172	1280.0008
1281.1141	1281.1637	1315.8762
1342.6981	1344.2740	1365.8597
1367.7268	1369.1763	1369.9754
1387.1118	1420.9562	1423.1930
1427.1272	1429.4341	1430.9470
1431.3710	1455.4734	1467.0573
1467.2863	1468.7877	1471.1040
1471.5830	1502.0290	1539.6461
1575.5899	1576.2110	1585.7083
1590.4739	1592.4670	1593.4080
1600.8249	1602.2559	1603.7629
1606.0006	1607.0985	1610.0748
1610.2519	3088.0211	3091.7458
3092.1123	3094.8235	3095.3987
3096.5884	3098.5900	3099.3284
3101.1362	3104.7652	3104.7860
3104.8416	3107.8274	3109.2528
3109.2752	3110.6114	3112.9812
3115.4241	3117.9940	3118.1452
3118.6182	3118.9806	3119.2929
3119.9572	3123.4148	3127.0093
3128.5777	3129.6510	3130.0878
3131.3531	3133.1764	3149.9038

THF

27.3299	285.3752	625.3634
631.7082	777.3086	843.0881
889.0967	911.2069	920.8268
947.7493	1019.6673	1082.4992
1106.5438	1165.4697	1180.7922
1208.5441	1219.9073	1254.8415
1257.7666	1304.5884	1342.7297
1410.5493	1430.7812	1434.6309
1452.2788	2867.2564	2873.9463
2988.0998	2996.7635	3026.4858
3030.3846	3039.7599	3054.1798

Styrene

57.1616	202.1538	225.1465
399.5985	435.3862	436.4051
542.8759	613.0843	637.2438
691.1673	771.1974	782.7596
824.9379	881.8509	906.9135
957.8674	978.5592	983.4224
995.0820	1001.0831	1024.8042
1075.2363	1136.1067	1154.9412
1200.7128	1271.0551	1295.6777
1364.4565	1389.1374	1434.9566
1479.8884	1583.2226	1612.0618
1649.1112	3060.7886	3071.4360
3090.8993	3098.0701	3107.1183
3114.4175	3123.2718	3164.7351
57.1616	202.1538	225.1464

399.5984 435.3862 436.4052
542.8759 613.0843 637.2437
691.1673 771.1974 782.7596
824.9380 881.8503 906.9137
957.8675 978.5592 983.4224
995.0822 1001.0831 1024.8042
1075.2363 1136.1067 1154.9413
1200.7128 1271.0552 1295.6779
1364.4563 1389.1370 1434.9566
1479.8885 1583.2227 1612.0618
1649.1112 3060.7885 3071.4360
3090.8992 3098.0700 3107.1183
3114.4175 3123.2718 3164.7352

Iminoiodinane

8.9633 17.4059 27.0923
30.4017 35.2744 71.8769
104.5429 128.2751 149.4215
166.0458 212.9888 221.3328
252.4172 261.5867 285.9374
324.6471 340.2710 394.1676
402.6955 405.4231 442.0799
450.7936 512.2449 523.3951
539.5338 596.6281 618.3134
625.9308 638.1012 683.4047
701.4447 721.2339 729.5870
796.6356 800.4880 823.3990
824.2208 906.7562 941.4804
957.9627 958.4693 962.8073
968.4793 996.4406 997.5810
1010.7027 1011.3164 1036.0864
1038.4387 1069.4297 1086.1270
1090.9224 1138.3859 1150.2987
1161.1524 1204.7098 1236.8459
1262.1901 1289.1193 1343.2096
1356.0697 1359.7499 1391.6045
1408.1958 1421.0775 1425.5698
1449.3092 1473.0299 1570.7312
1583.3645 1592.1738 1607.1956
2957.4071 3029.6614 3062.0675
3068.3485 3094.7382 3095.9450
3102.2805 3110.5702 3117.9509
3127.7168 3128.5785 3129.8706

PhI

145.5015 215.6894 261.3014
398.4002 448.5712 602.5265
647.8579 686.3160 721.4923
814.6159 889.6011 952.8784
972.5039 978.8148 1013.9153
1046.8459 1061.3340 1135.9803
1152.2738 1271.9733 1354.3551
1421.8353 1448.7950 1581.1403
1587.8650 3101.6178 3109.4325
3122.0420 3128.3959 3131.1772

Int A iso1 S=1/2

10.7722 14.0930 20.0574
22.3736 30.0107 33.0367
34.5917 37.8124 40.4587
46.4894 47.2199 48.8434
51.7811 54.4542 58.7810
61.6898 63.2300 71.1019
72.0736 75.8003 80.7070
84.6801 90.5449 98.5994
102.5535 107.3838 109.2762
109.6463 117.2059 121.0773
127.8597 137.6476 147.5417
163.2029 169.1885 174.8366
183.5426 194.5975 205.6551
210.8860 218.1687 220.2235
239.0470 250.2782 254.2636
258.6271 261.3655 265.4638
267.7908 279.7747 286.1487
289.1469 296.4241 312.4443
320.1095 321.7941 329.1268
339.2566 343.3485 352.6786
369.7863 381.4211 392.3776
395.8008 397.5462 401.3780
401.5240 407.0085 409.1620
416.8387 421.4960 423.4673
441.8197 452.6157 464.4338
473.6840 494.7642 496.8493
501.2616 503.2362 509.1265
520.9439 527.8568 539.3359
541.6522 553.8271 580.3037
580.4908 604.7853 611.0411
611.9435 612.3527 613.7804
616.5793 622.5295 624.2962
631.2758 632.3592 634.3603
635.8078 646.3472 649.4184
664.0797 669.9256 689.6302
691.8790 697.7496 698.2943
701.5668 703.4199 711.4347
723.3561 723.9078 734.1706
738.4209 742.7412 746.8295
748.9010 753.8398 758.0924
763.9170 779.7999 783.5764
786.7567 793.6147 806.2202
810.1060 815.6852 821.0428
822.5006 823.6846 829.1020
831.3191 832.8203 834.9312
837.9833 858.6664 869.6421
876.2435 888.0388 893.1991
899.5193 900.5995 917.3343
919.2438 922.4674 932.6387
932.8578 936.7597 942.8004
945.9827 950.0013 950.2866
950.7109 956.9412 957.6682

962.8839	964.7186	965.3947
975.2539	975.6274	976.4338
977.8416	978.5728	978.7008
979.3969	980.0355	983.1430
985.2330	988.3335	988.7785
990.1035	992.4160	998.3198
1005.5437	1008.9125	1014.0146
1023.5906	1025.3731	1025.8699
1026.6054	1032.1562	1038.8478
1046.9349	1048.8976	1053.6065
1061.0914	1073.1019	1074.0765
1075.6684	1078.0821	1090.3714
1092.1721	1095.2029	1097.4948
1099.1524	1133.1450	1133.5291
1134.5661	1135.3683	1136.4817
1138.5154	1140.0361	1148.9083
1149.4545	1151.5865	1153.9558
1154.4729	1154.6087	1157.6531
1158.3967	1166.8832	1172.5445
1186.5335	1195.8443	1198.5291
1201.6273	1208.3793	1238.6278
1242.9355	1264.4848	1265.0149
1271.6558	1272.8471	1279.0167
1283.4966	1283.7918	1285.9665
1288.4992	1331.0243	1342.3350
1343.2278	1349.1233	1354.6217
1359.6740	1367.2846	1371.2816
1371.7293	1372.2508	1394.2102
1397.6443	1408.3161	1419.0869
1420.6333	1420.8597	1426.4879
1427.9856	1429.5885	1432.6596
1433.8342	1449.6474	1457.2141
1470.5662	1471.5145	1472.8745
1473.1506	1473.7752	1474.0268
1509.8564	1561.4873	1575.1195
1577.2713	1581.8416	1583.8744
1584.0310	1588.2788	1589.4991
1593.0760	1593.5044	1604.1295
1606.5360	1606.7394	1607.5898
1608.4276	1610.9617	1611.5032
1622.4877	2876.9613	2956.0120
3030.5230	3066.5310	3094.5263
3095.1939	3096.6640	3097.8269
3098.7177	3099.5052	3099.9961
3101.5619	3103.0322	3103.2844
3106.6844	3107.2615	3107.3176
3107.9003	3108.7434	3110.1988
3111.9280	3112.3866	3114.3726
3115.8298	3116.9535	3118.0473
3118.2029	3120.2319	3120.6720
3121.3534	3121.4217	3121.6791
3124.1702	3124.5409	3125.5116
3127.2045	3128.1964	3129.2222
3130.1987	3138.1439	3141.3217
3144.9339	3153.5148	3160.8284

Int A iso2 S=1/2

-7.8332	16.3392	21.2507
24.6092	26.3437	33.8022
38.3950	39.4638	40.3572
43.8287	46.0044	47.9938
53.2598	55.4792	59.0619
62.9402	63.9263	65.1430
73.1531	76.5220	78.1785
86.0408	87.7600	90.4037
94.0279	95.3631	103.8589
108.3079	115.2451	115.9166
119.6341	124.4020	130.8026
138.7393	157.8990	164.8898
172.6234	184.9657	203.7405
208.3842	212.5857	217.9077
220.8954	242.6563	243.7539
252.8901	255.3385	258.6711
259.9566	267.1785	271.2291
278.3553	298.0410	300.8811
309.0434	310.5926	315.6111
326.9680	333.9420	349.7539
357.9911	384.1131	394.6869
396.3678	397.1958	399.2138
402.2052	404.8282	409.4465
410.7659	411.6270	414.9297
428.0740	430.3535	448.4079
458.0962	462.7449	482.1247
495.8233	498.1684	504.8625
507.4909	525.7615	532.6539
539.2242	551.6885	557.2263
572.0182	603.6376	611.9246
612.1473	612.7761	613.0611
614.1896	625.5819	626.2248
628.1688	632.1563	634.7183
637.1031	638.1826	647.8514
654.2328	663.4012	683.8346
689.0927	696.5025	699.0203
706.2091	707.8926	714.0879
716.4988	720.0426	720.9938
734.0313	748.3850	749.6473
751.4755	756.7087	760.2121
761.5288	765.7830	773.9809
782.1082	790.1990	794.5582
802.7923	816.6344	817.0212
824.9871	826.7354	832.2653
840.6575	845.8021	849.5461
866.3397	868.3544	886.7375
889.3191	890.5676	903.4032
907.0506	925.5825	928.6271
928.7479	933.2630	935.9564
942.9119	947.6925	948.2908
951.5475	953.9642	955.8841
956.6288	964.2216	966.4153
968.3644	971.3225	973.4388
975.8867	978.6487	979.1612

980.6569	983.1450	984.8549
986.0615	988.3100	989.1755
989.7489	993.7019	995.6109
998.7336	1002.3186	1009.5719
1010.8388	1015.3010	1021.8710
1024.6869	1025.7147	1026.5071
1027.9675	1034.0619	1046.1597
1046.7775	1055.6019	1063.4270
1065.8807	1070.2508	1073.5306
1074.5498	1084.4817	1092.3461
1094.6585	1097.9223	1100.9209
1105.6674	1132.9246	1133.3261
1134.1459	1134.2778	1135.9739
1138.5790	1141.8986	1146.1035
1147.8529	1149.5939	1152.8004
1153.6092	1155.6147	1159.3537
1159.4299	1161.7505	1172.0558
1180.5061	1184.1908	1190.4709
1202.3333	1206.9690	1240.2012
1244.7795	1257.2746	1263.4422
1272.2401	1272.9368	1278.6172
1281.1691	1283.1532	1287.1948
1289.2612	1324.4746	1341.6677
1345.4530	1349.8163	1355.5995
1357.2578	1367.3299	1368.1780
1370.9423	1372.9195	1393.1285
1404.3483	1406.5707	1420.8451
1421.1919	1422.1596	1427.5350
1428.3736	1428.5818	1432.0984
1433.4735	1449.8007	1455.8177
1466.8662	1470.0058	1471.3704
1472.3040	1472.9655	1473.1740
1500.4672	1540.3817	1574.8341
1576.3513	1577.2218	1581.6889
1585.1650	1587.5643	1590.9248
1592.2208	1595.1997	1602.3392
1604.6206	1604.9028	1605.3384
1607.0604	1609.3936	1611.0387
1612.4989	2956.2569	3028.7182
3061.5449	3087.8024	3094.6401
3095.7571	3096.2704	3096.3435
3097.0411	3097.8885	3098.3820
3098.6840	3098.7971	3105.1699
3105.4031	3105.6270	3107.2381
3107.5992	3108.4866	3109.1751
3110.0663	3115.2929	3115.5907
3116.0022	3116.0649	3117.0920
3118.4502	3119.7234	3120.4033
3123.4436	3124.3011	3124.6503
3125.2520	3127.7381	3128.2993
3131.0078	3131.2810	3132.8849
3133.0173	3143.1126	3149.7738
3150.1101	3151.1543	3162.5498

Int A iso1 S=3/2

-11.5895	12.5873	15.1835
16.6464	23.2670	29.4052
32.7013	37.0845	39.4356
40.6513	44.4944	50.1329
50.7866	54.3845	55.5995
56.5947	62.8665	64.4782
72.9651	75.8553	79.5215
80.1047	85.7870	88.6595
93.9465	100.6820	103.8235
106.7404	109.4436	115.0534
118.7304	125.7404	130.1948
151.1472	165.9707	172.1961
181.9170	197.4378	200.6797
207.2111	215.6560	220.0119
224.4754	240.8399	248.7805
249.2202	256.9806	260.8747
261.6024	268.3902	275.4550
285.6854	288.6432	296.0489
305.8973	321.6876	328.2253
338.8809	347.1643	356.4847
361.2953	372.4296	389.7754
392.5813	398.2531	401.4498
402.5897	405.3710	406.6632
408.4744	410.9460	418.4231
420.3041	452.5858	464.6389
476.2569	478.2928	492.7685
502.2997	508.7419	510.6617
516.6111	521.4091	525.7904
541.0749	552.8903	580.8974
604.6071	611.2184	611.6169
612.3584	613.5540	616.3268
624.2400	627.7235	627.9068
630.2795	633.1860	634.5602
637.0171	649.2049	661.3066
666.0749	689.8492	691.6306
694.1123	697.0056	700.8196
703.9366	706.7617	720.2577
723.9940	729.7555	736.5952
740.0464	742.6870	748.0135
749.2550	752.4667	755.3393
761.1277	766.4712	780.8560
787.5361	794.3026	801.9767
813.4393	814.2472	818.6693
821.1079	824.9036	829.8669
831.0884	836.0020	841.0919
863.5411	869.4643	880.5589
890.8703	895.7840	900.3527
905.3878	920.6713	926.1867
926.4953	929.6874	930.6477
942.1160	946.9190	947.2411
952.0728	957.7853	958.2812
958.7995	960.1026	963.5039
964.3224	969.4672	975.3034
975.5586	978.5043	978.9637
979.7466	980.2614	982.1066
984.6609	985.8414	986.7783

987.4877	988.3093	989.6512
989.8798	1001.7490	1004.2123
1009.4103	1011.6806	1014.0327
1023.4407	1024.9671	1027.0923
1027.4288	1029.6298	1030.4706
1047.2415	1048.6959	1051.7469
1062.1930	1074.1479	1075.8007
1076.3652	1082.4892	1085.2818
1091.6084	1096.2379	1096.6311
1099.5125	1133.4105	1134.7429
1135.0404	1135.9764	1136.9311
1138.5976	1140.5182	1148.0300
1148.3503	1152.7659	1154.5989
1155.5393	1155.8153	1156.0160
1160.1873	1161.3129	1170.5624
1179.2513	1189.8429	1194.6425
1207.4335	1214.8283	1240.8369
1242.3759	1264.7854	1266.3164
1273.8894	1273.9316	1280.2664
1283.4601	1283.9757	1284.7086
1289.9575	1330.3585	1342.5809
1345.1152	1347.7439	1354.9711
1357.3669	1366.6409	1370.5033
1371.8602	1372.4658	1394.0685
1398.6264	1407.4969	1420.6981
1421.2986	1421.4056	1426.9431
1429.0284	1430.0031	1433.3439
1433.7878	1449.9692	1456.7164
1469.9301	1471.9125	1472.4467
1473.5397	1474.9999	1475.9438
1508.9821	1559.6527	1575.2299
1577.5381	1581.2285	1583.2649
1584.0595	1588.8132	1591.3719
1592.7054	1593.9812	1605.8538
1606.5489	1607.0867	1608.0656
1608.3591	1610.6947	1611.9856
1621.1920	2957.7217	3029.7940
3063.3160	3078.6963	3095.7584
3096.6564	3097.0736	3098.0019
3098.1146	3099.4789	3099.6822
3100.0341	3101.9126	3103.7135
3104.0802	3105.2141	3107.4324
3107.9799	3108.7568	3110.0430
3112.0882	3112.8859	3114.6598
3116.3890	3117.7659	3118.6010
3118.7176	3119.3397	3121.2110
3121.4139	3121.8059	3123.6779
3124.4188	3124.6011	3127.7898
3127.9425	3128.6815	3129.2379
3132.2485	3135.1886	3135.3388
3136.5014	3143.7195	3144.1589

Int A iso2 S=3/2

-1.3734	11.5774	14.8249
20.6795	21.7123	30.1294
33.6427	36.4232	40.5190
42.0685	47.6142	50.5131
51.4247	56.0623	57.0797
58.6250	62.9936	70.2350
70.6939	74.6160	79.4045
80.9767	83.3715	86.5129
91.7643	94.9426	99.7061
105.2350	110.1525	114.0381
117.2190	123.5273	127.3264
138.1857	154.1727	165.5065
178.3682	184.6299	193.0929
205.9219	217.6161	220.1769
222.7685	236.8367	246.2345
252.7514	260.0942	260.8790
262.5617	272.3811	278.0523
282.6556	286.4235	300.7598
305.7280	313.3226	325.4935
332.4756	345.2954	352.3430
360.5616	377.5650	396.1100
397.5640	398.5404	399.9561
402.1184	404.4121	407.0965
408.6461	418.1900	419.6666
420.8439	442.3613	451.0645
466.4276	479.1551	490.8652
492.7878	501.2191	508.4771
517.2390	520.1735	527.8371
540.9098	554.3019	579.6610
598.1955	604.3043	612.0670
612.9011	613.5419	614.1316
615.6736	625.4994	628.1799
631.5036	633.2164	633.6508
635.9789	649.2387	654.6447
665.1268	666.9810	686.9721
689.7543	691.9993	694.1640
696.3759	703.2396	712.3675
721.5385	722.9507	734.7589
737.9492	748.3035	748.8656
752.5343	755.6917	760.3768
766.1338	779.5750	779.9988
791.6485	800.5278	809.7782
810.9311	814.0282	819.7631
820.7475	821.8864	828.6713
832.9362	834.4763	847.2348
864.2953	869.1934	879.2234
892.0904	894.3999	897.7636
910.7033	919.8491	920.5490
926.3766	930.7153	932.6650
940.9201	946.3966	946.9951
955.5925	956.4982	958.9307
960.2501	960.3848	962.7257
965.3395	972.8317	974.9433
978.0007	978.4743	978.5437
980.1674	981.8186	984.3510
985.4277	986.6833	987.9234
988.8100	993.6038	994.2554
998.1019	1002.6588	1006.5078

1010.3898 1014.3212 1023.6909
1026.2778 1026.8417 1028.5068
1029.8533 1032.8778 1047.8827
1048.6435 1049.1906 1052.3778
1062.6641 1072.9282 1075.7029
1079.1915 1083.0152 1090.0232
1094.6726 1095.6596 1098.6713
1101.5520 1131.6001 1133.4031
1135.6665 1136.3966 1137.7565
1138.6578 1138.9751 1145.7305
1147.6545 1152.7185 1153.1614
1154.4951 1156.0432 1159.2185
1160.9717 1164.4425 1178.6812
1179.3834 1189.4494 1194.2545
1207.9041 1238.4066 1242.3006
1258.5129 1264.4224 1269.8117
1272.2370 1274.5432 1277.2665
1282.4145 1283.5216 1284.2640
1293.6602 1330.8769 1342.4926
1343.8571 1349.0318 1356.0347
1357.4542 1366.0107 1370.9784
1371.5466 1371.9357 1396.2409
1397.9491 1407.4250 1420.6141
1420.7858 1421.2693 1426.1685
1426.5982 1429.0104 1433.1109
1435.0029 1450.6068 1456.9816
1470.7076 1471.0480 1471.8297
1472.7816 1474.4800 1474.7489
1508.7883 1559.8811 1574.9416
1576.1948 1577.5694 1581.4562
1584.8397 1586.8954 1591.3690
1593.7337 1594.4910 1604.5285
1605.5377 1606.0382 1608.3073
1608.3310 1610.6996 1612.8274
1621.5934 2958.2672 3029.8571
3066.0468 3068.5259 3086.2015
3095.4895 3096.0459 3096.0526
3096.4167 3097.6010 3099.6510
3099.7321 3100.6851 3105.0809
3105.6499 3106.1835 3106.5892
3106.9115 3108.3986 3108.5074
3110.7075 3112.1138 3113.5399
3114.0376 3114.5574 3117.6270
3118.0033 3120.5490 3120.7748
3123.3924 3124.4410 3125.0423
3126.5526 3126.9725 3127.8509
3130.1655 3131.2684 3133.8141
3135.1582 3137.3718 3137.8471
3139.5424 3141.2562 3186.9064

Int A iso1 S=5/2

-8.7406 9.6069 14.4370
20.2149 23.9539 30.1688
32.6155 38.9219 40.4722
43.6067 44.0422 46.5910
49.7549 53.9732 57.9193
61.6213 62.2032 65.6734
68.9355 71.9749 74.5181
80.7932 88.5101 91.3031
97.1413 99.3250 106.2747
107.7944 111.3813 114.3217
121.2651 125.4566 129.6196
142.8983 165.4208 170.8437
185.3966 192.2766 198.4455
206.6718 210.6324 213.8087
215.6172 236.3442 238.9401
242.5267 249.0091 253.7284
259.3425 265.5725 273.8852
280.8255 285.6825 293.6426
300.9497 317.7974 330.7571
336.6177 339.6399 343.2995
356.8780 358.0058 383.9820
391.2712 393.1845 398.8465
402.7406 402.8157 408.9719
412.0260 414.6486 418.5443
421.8655 448.6677 462.4862
472.1121 475.9978 490.0816
499.7279 502.5552 506.5177
508.8972 518.6623 526.7681
541.0974 552.5292 574.4387
577.8555 601.0727 610.6611
611.3924 612.0220 612.8478
614.2008 624.8463 625.6258
629.3247 630.7089 631.6537
633.2058 639.0833 646.1053
655.0554 663.1917 681.0281
694.1091 694.1633 700.0602
701.6353 701.7531 709.6318
717.9754 722.5740 729.4997
732.5247 743.7960 745.9568
750.9835 752.7354 757.7708
763.0338 766.7954 777.2106
778.8922 786.6503 795.4113
804.0883 814.9382 819.2210
824.1777 828.5800 831.1630
833.0122 838.9239 840.0845
859.9791 867.3876 882.1443
889.5200 894.5614 898.9937
902.4732 920.4974 924.0721
926.4345 929.3225 930.8600
942.0978 943.1811 944.4665
952.3644 954.0325 955.5995
958.1379 959.5952 962.7326
965.6720 967.2578 968.5055
972.3765 976.0729 977.5661
977.6433 978.6711 982.2268
984.6402 985.1048 987.0239
988.0062 988.7759 990.2643
990.9302 1000.5103 1000.9412
1009.4475 1012.0835 1020.4118
1025.0913 1025.9869 1026.4218

1027.2850 1040.9793 1044.8951
1046.8397 1058.1585 1063.1500
1068.6333 1073.2769 1073.6545
1082.7184 1087.2540 1090.6190
1093.9144 1094.8656 1096.0903
1107.5091 1133.7800 1133.9560
1134.6347 1135.0985 1136.0571
1138.7856 1139.0510 1147.1254
1149.6283 1151.1751 1153.1733
1154.0866 1154.3844 1155.3077
1159.2529 1159.7138 1168.5362
1175.3428 1178.7756 1183.2319
1196.3592 1207.0773 1239.2453
1243.1289 1261.9654 1264.3139
1268.5865 1273.7629 1275.9847
1281.3646 1283.0662 1283.5010
1290.9124 1321.8593 1342.3022
1345.0153 1346.2882 1356.8566
1358.2633 1367.1010 1368.7974
1370.7924 1372.5945 1393.4980
1398.7231 1407.4863 1419.1868
1421.0537 1422.4160 1425.5665
1426.4315 1429.2660 1430.6433
1433.5210 1446.1224 1455.1840
1467.9430 1469.8970 1472.6198
1473.0427 1474.2791 1475.6073
1500.1494 1544.0818 1574.7057
1574.9263 1575.1252 1579.0566
1582.2480 1586.4550 1588.3237
1588.8034 1594.1084 1600.9837
1603.9185 1605.3705 1606.9567
1608.2324 1608.5679 1609.3420
1611.3235 2955.3646 3027.7393
3062.7966 3070.0791 3093.0675
3093.4863 3096.2224 3096.6490
3096.8842 3097.5667 3097.6681
3098.8437 3099.0062 3102.4694
3103.9498 3105.8728 3105.9062
3106.8363 3107.0137 3108.6320
3109.6881 3110.4712 3111.4216
3111.7715 3113.3763 3116.2680
3117.0759 3117.1654 3118.3698
3118.5744 3119.4847 3122.5188
3123.0488 3123.6038 3124.1689
3124.5031 3124.7622 3125.3089
3126.9785 3135.8133 3139.0836
3139.5980 3143.8465 3144.1232

Int A iso2 S=5/2

-7.0820 15.7338 20.6383
24.1398 28.1098 32.8468
40.6068 41.6734 42.7760
44.1132 48.6301 51.3185
56.5267 60.5192 60.8889
66.8174 68.8311 69.8735
73.7220 77.5345 79.3564
85.7688 87.3145 91.5958
97.3865 100.2603 100.8994
106.3603 110.8636 114.6383
121.3341 133.6292 137.0660
152.6644 162.2398 181.7647
184.0252 192.5395 202.6258
210.4693 211.4906 221.0074
224.5733 234.1204 239.7556
248.8159 258.9147 261.1803
267.1744 274.2437 277.2516
281.6529 284.6885 297.2275
307.7485 320.2786 323.4346
331.8082 341.4250 346.3716
351.7045 357.8294 375.0009
381.1697 394.5739 395.2710
397.3877 403.4640 405.0052
409.2502 413.4383 417.7684
418.0651 421.8051 447.3401
466.4436 475.5474 488.7892
500.5548 503.4609 508.0413
511.4813 515.7912 526.6861
540.8725 555.2866 573.6746
582.8007 602.8292 611.3816
612.7192 613.3051 614.3179
616.3488 625.6258 627.7696
630.3119 632.0712 633.5247
634.5302 641.2850 647.2849
662.6286 667.0614 683.0290
694.0547 695.3274 702.2744
703.4316 707.1784 710.3334
721.3413 727.0262 733.8011
738.7445 747.4701 749.1077
752.5881 756.4232 760.4604
766.9243 779.4223 789.7859
798.6245 802.7014 814.0376
820.3568 822.8337 828.9058
831.5161 832.7661 837.2230
840.6740 844.2448 855.1686
862.6404 869.1842 883.9470
889.0074 896.9753 900.3362
909.7383 922.1222 924.4623
929.4477 931.0846 932.3780
943.4085 945.0583 946.4650
952.2451 956.1605 960.6061
961.2219 965.5912 965.8806
968.2476 970.8986 972.2948
976.3624 977.2279 979.3501
981.0526 984.8498 985.5329
987.0776 988.3311 990.4324
990.6649 992.3277 994.5009
998.8567 1003.5616 1010.0620
1015.2927 1015.4791 1025.7987
1026.4056 1027.3273 1028.0735
1029.0375 1042.5281 1045.5406
1047.0686 1049.7281 1059.1581

1066.0953 1074.1551 1075.7084
1079.5256 1085.8450 1087.4488
1089.8804 1093.6268 1095.4353
1098.7140 1134.1784 1135.2476
1135.9758 1136.0692 1137.2941
1137.6179 1139.2060 1146.2056
1147.7737 1151.6962 1154.4652
1156.7444 1157.9528 1160.1883
1161.6670 1164.7092 1176.7848
1183.6486 1187.1710 1197.7783
1207.5557 1236.4956 1238.9934
1240.8582 1262.0051 1267.6672
1270.7773 1274.6032 1276.1386
1282.1533 1286.3615 1287.6099
1293.6876 1330.2948 1341.3521
1342.7414 1347.0334 1356.7062
1359.2252 1367.3507 1369.7976
1370.6536 1372.1732 1392.1057
1393.3187 1404.2573 1419.5861
1421.2797 1422.1439 1427.0444
1427.5308 1430.1703 1433.2884
1434.2196 1450.5084 1455.9050
1469.6502 1472.9395 1473.5016
1474.3927 1474.6639 1476.1653
1504.6747 1556.0867 1573.4925
1576.3399 1580.9989 1581.3101
1588.3782 1588.3885 1592.1601
1593.2334 1593.6935 1605.3093
1605.9638 1606.8599 1607.9202
1608.6339 1610.5002 1612.7339
1613.6486 2960.8179 3032.9172
3074.1716 3096.2749 3097.2254
3097.3250 3097.5161 3097.7148
3097.9690 3100.2491 3100.5499
3104.5219 3104.9503 3105.7347
3105.9694 3108.9760 3109.4266
3109.5312 3111.1279 3111.3758
3112.8472 3112.8937 3113.3265
3115.6848 3118.4160 3120.2475
3120.7212 3120.9163 3121.1822
3122.7897 3123.1002 3125.2032
3126.6778 3128.2009 3128.4680
3129.8832 3129.9162 3130.8940
3130.9421 3139.2065 3143.4978
3148.2408 3151.7855 3162.5781

Int B iso1 S=1/2

-9.9971 18.7044 23.3863
28.5431 31.2369 33.8160
37.9830 42.3557 46.0423
48.2099 51.0141 52.3027
61.5980 65.0275 68.7748
73.4037 75.5913 79.8688
82.7571 87.2911 87.9687
93.1518 103.9685 107.2011
113.9570 122.4352 126.6204
160.0214 169.0018 173.8475
192.0144 205.0998 208.5479
217.0923 226.2935 239.6406
244.0930 252.1150 256.2525
269.6944 273.6645 283.2636
289.0784 297.2603 301.5999
309.5782 317.1887 324.7392
335.1587 342.6591 344.9526
358.8434 363.4337 382.9573
396.8171 400.8888 401.4216
404.3864 405.9746 409.8297
414.6787 422.6455 424.7429
449.5461 465.5630 476.3988
487.7554 498.2378 504.1863
507.2648 511.8037 521.7903
527.5756 540.3148 552.7706
559.1444 579.5143 611.7264
611.9195 613.4690 614.1572
616.6405 624.4673 628.6434
629.7826 632.0806 633.6492
636.4814 637.9488 666.6396
670.0207 693.4017 694.6756
698.1403 701.4701 703.2499
704.9814 728.6379 736.1260
740.2108 747.6281 749.1933
752.1879 755.1840 760.3334
767.2321 780.9264 790.3266
799.2884 800.9873 819.6193
820.0830 824.4946 825.3535
827.7789 828.5208 834.4689
836.7518 863.8089 868.1202
889.2860 893.9297 900.4113
905.0961 922.3702 923.7857
930.0299 930.8313 937.4811
940.3006 946.6591 947.1980
955.5893 955.9997 957.3455
957.8568 963.1636 963.3310
966.0825 967.5589 977.4026
978.5266 978.6499 980.1844
981.7434 983.1231 984.2170
987.6651 988.1333 989.5792
991.0298 992.4200 997.6726
1004.7169 1008.9262 1022.0873
1024.9905 1026.7347 1028.0123
1028.6879 1030.1154 1047.3234
1052.6018 1053.6652 1072.1745
1074.9440 1075.1565 1079.4541
1090.8004 1093.4142 1097.5793
1099.7765 1104.6704 1134.5081
1134.5513 1135.6364 1137.0590
1138.5386 1141.0184 1147.6393
1148.2389 1153.4235 1153.8368
1157.2347 1157.9168 1159.3055
1161.6381 1174.0652 1185.1985
1191.0120 1192.3278 1207.6894

1241.4801	1243.8819	1259.9310
1263.5989	1274.5787	1275.2126
1281.9374	1282.5632	1285.1957
1287.7192	1288.9573	1329.5088
1342.5276	1343.8816	1346.1720
1357.9608	1367.4092	1368.7651
1372.7677	1373.0631	1394.1292
1399.1100	1411.7514	1418.3526
1421.9648	1427.6142	1429.7036
1431.9609	1433.1814	1434.0229
1457.3276	1468.9510	1472.6709
1472.8735	1473.5751	1474.3246
1475.1696	1508.5443	1559.6155
1575.3706	1576.8077	1583.8210
1590.4960	1591.6660	1594.0098
1595.9369	1605.7975	1607.0166
1607.2525	1607.8865	1609.4483
1611.2726	1612.9639	1621.4943
2955.8449	3032.8215	3066.4623
3093.8516	3096.7176	3097.4369
3097.4721	3098.2069	3098.3715
3099.4162	3099.6296	3104.4418
3105.4579	3107.1813	3108.2216
3111.9556	3112.4057	3112.7591
3113.5675	3113.7973	3118.0065
3119.0298	3120.2224	3120.3862
3120.8422	3121.0593	3124.7770
3126.1337	3126.7141	3126.7939
3127.4557	3130.5630	3132.5387
3133.0227	3133.3305	3134.9570
3137.5789	3137.9249	3139.8358

Int B iso2 S=1/2

-11.4471	-5.7145	14.3193
20.0089	21.5970	26.7357
32.0267	34.5811	38.0766
42.0716	47.4909	53.4131
55.2397	59.0148	61.9068
67.1487	72.5375	77.2970
82.3575	85.8030	90.1640
93.7933	99.0992	107.5916
114.9407	121.8735	138.1820
149.1613	168.2223	178.2720
188.6896	197.0363	201.4015
212.2822	226.1820	232.0343
242.0859	250.2943	253.5273
259.6409	272.1433	280.6505
288.7294	293.5168	301.6624
304.6321	319.3166	326.1777
336.4420	342.5861	350.2541
358.8057	376.5335	382.7986
394.9239	397.7035	400.7087
401.7542	403.6601	407.0862
413.4562	421.4500	425.6433
440.6239	465.7274	480.0937
496.4203	502.1373	507.5961
508.8277	512.7988	518.8295
530.0397	539.8237	552.1593
575.9757	600.3124	611.3418
611.9135	612.6681	612.8069
616.3110	623.8812	628.3184
630.4481	632.0735	635.9745
636.3618	648.4018	665.9366
669.1064	696.3391	697.3180
699.2781	700.6021	704.7565
710.0728	727.6586	735.3591
740.1712	748.3143	749.9420
752.0931	756.3688	758.7423
767.0984	780.0219	795.3550
799.8528	803.6340	818.5430
820.9420	823.4218	827.3311
830.2953	834.7630	838.3411
844.5295	864.9707	866.2469
881.9709	892.2389	894.2709
906.7306	907.4621	921.8207
925.9163	929.2872	931.1964
938.3472	938.6681	947.1336
947.4593	952.9755	954.2919
957.0816	959.0167	962.6870
967.1992	969.7569	977.2649
978.3374	978.8434	980.1694
980.9696	983.7232	985.8981
987.9036	989.3183	992.8875
996.7961	997.1857	999.0679
1003.9694	1010.8359	1013.7562
1023.5687	1024.8061	1026.4276
1027.0654	1029.9227	1047.1764
1049.4042	1050.5753	1073.0922
1075.1391	1076.7430	1081.1021
1090.8519	1093.3202	1097.3314
1098.8278	1099.6429	1134.1662
1135.5073	1136.0673	1137.0772
1139.7145	1140.0806	1147.8955
1148.8634	1153.4099	1154.6112
1156.9473	1158.3348	1160.3535
1166.8012	1179.3775	1187.6663
1194.6639	1199.8172	1205.8500
1222.3422	1240.2149	1243.4338
1263.7367	1264.6269	1274.9333
1282.7075	1283.2771	1286.1300
1288.4632	1296.4906	1328.1361
1343.2153	1343.4790	1348.4101
1359.3818	1369.0392	1369.2516
1372.4616	1374.6986	1393.1290
1399.7421	1407.8473	1421.8068
1422.4637	1425.6364	1430.3740
1431.0147	1434.4974	1435.1506
1458.4903	1470.4339	1473.2938
1474.8396	1475.1113	1476.0385
1476.9784	1508.4110	1556.8924
1575.3129	1576.8947	1584.6138

1591.1888	1591.5270	1595.2066
1596.5896	1606.5922	1607.4303
1608.2219	1608.8369	1608.8882
1611.5189	1613.3706	1619.1118
2957.6125	3029.8517	3063.2699
3096.1380	3096.1681	3096.1908
3096.6540	3097.3514	3097.3665
3098.1499	3099.1301	3100.4820
3105.0233	3107.5775	3107.7560
3108.6320	3109.9134	3111.3371
3113.8510	3114.3932	3114.8423
3117.1144	3117.6855	3119.0744
3119.0932	3119.3660	3122.2479
3123.8376	3125.3803	3126.5124
3127.6472	3128.7314	3129.4809
3129.5684	3130.0416	3131.3613
3133.6412	3134.9796	3137.3017

Int B iso1 S=3/2

-12.5717	6.4085	15.9765
20.5450	23.1910	30.7842
36.1940	41.3006	44.2730
46.9064	49.6945	56.2139
57.7100	65.2262	66.7581
74.1406	81.4172	84.4533
88.4618	93.5965	97.4730
101.5431	109.4503	112.7064
114.3767	123.2446	132.9723
135.8396	168.6622	174.6693
182.1093	201.1624	203.9450
211.6786	226.8786	234.9778
244.0094	251.0427	259.9548
265.5425	271.5578	276.3759
282.9497	287.4917	304.2052
307.5614	318.1614	320.3522
327.8727	341.9853	348.4756
358.7457	362.6258	384.6976
395.2209	399.5296	401.5620
402.4147	404.4961	413.7276
414.6431	424.5102	426.3166
446.5341	467.9943	481.8691
491.1419	500.6171	504.4362
510.6363	522.3028	527.8649
536.1171	545.9469	552.0108
578.6879	611.3043	611.4355
612.8367	613.8120	616.3894
623.0725	624.2141	628.2738
630.3332	632.4058	634.3461
636.4909	662.2540	667.4481
689.9212	691.4768	694.5575
699.7343	700.3508	701.1928
706.2151	720.3114	733.2093
737.7733	745.0204	747.9603
750.6553	753.6564	754.1229
762.2197	766.1864	781.7074
787.9091	796.1545	801.0275
808.1460	820.2733	821.1685
821.7565	825.4725	827.5804
836.8268	840.1881	857.9765
866.8107	885.4484	891.8416
900.7888	907.9558	919.9656
922.7549	925.8560	928.7436
937.0699	939.8415	944.3620
945.0823	953.6399	956.3750
956.7180	957.1642	962.7480
964.4320	967.3536	976.4301
977.3621	979.0504	980.3015
981.6918	984.2397	985.0198
986.7517	988.0351	988.6217
989.8570	990.3293	998.6789
1004.3880	1007.2089	1010.1462
1015.8857	1023.7060	1024.7561
1026.1975	1027.1338	1029.5782
1044.8690	1054.1887	1070.4216
1074.6661	1075.6862	1083.4045
1087.3274	1091.1547	1091.3189
1096.4404	1100.2718	1133.6371
1133.9541	1135.7686	1136.7178
1137.8676	1141.9927	1147.3891
1148.2722	1152.0011	1152.5437
1154.7057	1157.4223	1157.5330
1163.9409	1164.4905	1180.8999
1196.8759	1203.6238	1207.2694
1233.8198	1239.2471	1241.3974
1263.8956	1265.3310	1271.3244
1280.3657	1284.9442	1287.6219
1288.3063	1288.9582	1330.6012
1340.3231	1346.4772	1351.5793
1360.3059	1365.5742	1366.9236
1371.3701	1374.2966	1395.0899
1401.3344	1405.0696	1417.8756
1421.6032	1426.8138	1428.3092
1429.8295	1434.5651	1434.7224
1458.2775	1471.0782	1472.4454
1473.1081	1473.3724	1474.1382
1474.7953	1510.2703	1559.7135
1576.1527	1579.4048	1582.8243
1587.6952	1590.6539	1592.5920
1594.7327	1605.3945	1606.4708
1606.8453	1608.3251	1609.2598
1611.5008	1612.0597	1622.3274
2959.2986	3032.5101	3065.3398
3094.1190	3096.9531	3097.1676
3097.2652	3098.0351	3098.3663
3100.5323	3101.8854	3101.9927
3103.9331	3106.9669	3107.1982
3108.0338	3108.9068	3109.2750
3112.4225	3114.4131	3117.6551
3118.4665	3119.2543	3119.7563
3120.4118	3120.5546	3124.8522
3125.8672	3126.0171	3126.2520

3128.6655 3129.8071 3129.9259
3130.2139 3131.2406 3133.7394
3135.3569 3142.3946 3145.4371

Int B iso2 S=3/2

-12.5779 6.4155 16.0207
20.5813 23.2162 30.7740
36.1936 41.3071 44.2707
46.9018 49.6903 56.2131
57.7080 65.2177 66.7494
74.1268 81.3862 84.4459
88.4549 93.5740 97.4608
101.5358 109.4410 112.7041
114.3598 123.2361 132.9662
135.8094 168.6548 174.6580
182.1099 201.1593 203.9480
211.6730 226.8795 234.9744
244.0108 251.0397 259.9574
265.5443 271.5524 276.3774
282.9503 287.4924 304.2059
307.5626 318.1624 320.3550
327.8681 341.9817 348.4746
358.7416 362.6238 384.6981
395.2240 399.5342 401.5553
402.4111 404.4900 413.7186
414.6379 424.5066 426.3143
446.5344 467.9922 481.8688
491.1437 500.6175 504.4348
510.6341 522.3047 527.8671
536.1152 545.9414 552.0114
578.6842 611.3057 611.4354
612.8363 613.8105 616.3896
623.0722 624.2144 628.2755
630.3333 632.4063 634.3461
636.4905 662.2503 667.4472
689.9231 691.4944 694.5545
699.7325 700.3523 701.1892
706.2125 720.3082 733.2082
737.7722 745.0202 747.9616
750.6550 753.6543 754.1232
762.2189 766.1869 781.7075
787.9061 796.1475 801.0272
808.1450 820.2748 821.1860
821.7445 825.4770 827.5741
836.8240 840.1842 857.9743
866.8133 885.4611 891.8395
900.7979 907.9552 919.9672
922.7568 925.8616 928.7418
937.0642 939.8397 944.3602
945.0851 953.6372 956.3802
956.7102 957.1698 962.7465
964.4301 967.3525 976.4298
977.3619 979.0497 980.3007
981.6900 984.2386 985.0174
986.7527 988.0358 988.6239
989.8595 990.3288 998.6660
1004.3862 1007.2103 1010.1442
1015.8751 1023.7056 1024.7550
1026.1996 1027.1358 1029.5783
1044.8723 1054.1886 1070.4204
1074.6685 1075.6900 1083.4055
1087.3303 1091.1548 1091.3163
1096.4434 1100.2706 1133.6358
1133.9553 1135.7696 1136.7179
1137.8696 1141.9918 1147.3925
1148.2710 1152.0009 1152.5399
1154.7063 1157.4254 1157.5361
1163.9426 1164.4935 1180.9002
1196.8798 1203.6219 1207.2657
1233.8237 1239.2514 1241.3955
1263.8925 1265.3308 1271.3264
1280.3658 1284.9506 1287.6253
1288.3122 1288.9607 1330.6031
1340.3212 1346.4756 1351.5811
1360.3066 1365.5757 1366.9279
1371.3717 1374.2987 1395.0896
1401.3359 1405.0673 1417.8744
1421.6034 1426.8137 1428.3088
1429.8311 1434.5668 1434.7225
1458.2793 1471.0772 1472.4464
1473.1088 1473.3733 1474.1417
1474.8006 1510.2725 1559.7168
1576.1529 1579.4070 1582.8257
1587.6963 1590.6567 1592.5928
1594.7361 1605.3944 1606.4694
1606.8495 1608.3242 1609.2627
1611.5015 1612.0616 1622.3296
2959.2961 3032.5042 3065.3418
3094.1184 3096.9523 3097.1682
3097.2658 3098.0343 3098.3657
3100.5323 3101.8856 3102.0012
3103.9329 3106.9663 3107.2032
3108.0342 3108.9074 3109.2766
3112.4222 3114.4134 3117.6548
3118.4663 3119.2545 3119.7567
3120.4114 3120.5553 3124.8522
3125.8672 3126.0171 3126.2553
3128.6670 3129.8079 3129.9247
3130.2178 3131.2425 3133.7514
3135.3627 3142.3944 3145.4499

Int B iso1 S=5/2

-11.1933 6.5270 13.8881
16.2652 25.3187 30.3606
35.3092 40.2948 42.9234
47.2396 49.3565 56.9891
62.7287 66.3973 69.8141
73.9301 77.5411 79.2042
81.5652 91.5499 93.0829
98.1650 101.7556 106.9674

108.4271 121.5331 128.9182
129.1542 153.8442 167.8117
182.9863 192.7390 200.2938
208.5981 215.3426 234.1675
240.6666 250.4616 258.4876
262.0753 265.8367 270.7964
282.7666 294.7850 303.6212
305.7036 306.0031 318.2115
321.2939 339.9533 345.9544
354.8728 368.2141 395.9147
399.1892 401.6855 402.4526
402.8485 411.1595 412.2056
417.9953 420.7039 426.9974
437.7703 464.5092 470.4179
485.1069 490.4659 499.9564
509.8644 522.8626 526.3056
533.7146 541.8948 551.2877
555.3537 571.9139 611.2136
611.5057 612.1337 612.9064
613.8227 622.8109 624.3793
628.6207 630.1035 631.0444
633.7072 639.0406 656.1098
663.9226 694.6084 697.0703
699.1843 700.0848 701.2503
714.7096 718.0689 727.7382
731.3851 741.4174 746.3283
749.3853 754.1306 760.4579
763.8788 771.9004 778.2072
780.5755 792.2537 796.7894
802.6722 823.7064 825.1950
828.9753 833.3350 835.1573
835.5267 853.0663 856.3453
868.1461 888.5325 891.0596
903.5387 905.0280 919.0861
925.2407 930.4186 932.9984
940.8469 943.9126 944.8148
947.0018 957.8878 960.1410
961.7116 962.0915 963.6105
964.0528 969.7759 975.2244
978.0759 979.6192 981.8558
982.8035 985.2944 987.8883
988.3466 989.0956 990.5537
995.7094 996.9083 1000.5347
1009.9550 1019.9201 1022.4386
1023.5047 1023.8474 1026.6234
1027.4772 1034.6802 1043.7404
1051.2350 1063.2911 1073.1659
1073.9788 1081.0028 1087.7973
1089.4824 1091.4811 1092.0699
1096.3732 1100.0843 1133.4719
1133.4984 1134.0285 1137.9648
1138.0615 1140.6400 1145.9770
1148.8941 1150.5886 1151.8110
1155.2845 1155.6324 1156.2674
1162.4594 1165.3704 1184.6660
1188.8775 1195.0679 1205.5552
1205.7903 1236.9009 1244.5214
1259.8702 1261.7604 1268.2193
1279.4992 1282.5230 1285.7440
1287.4138 1288.3898 1318.7700
1342.1496 1345.6642 1348.4385
1358.6859 1366.1144 1367.1691
1369.4524 1371.5384 1394.8943
1401.9562 1407.1661 1420.4787
1422.4777 1424.7472 1428.1754
1428.8184 1431.7379 1433.9767
1455.0015 1467.6904 1470.3292
1471.7551 1472.3479 1473.9116
1474.8350 1500.1574 1540.0985
1574.8008 1575.7946 1579.7089
1586.3610 1587.5915 1592.8501
1593.2737 1601.7692 1603.2067
1604.2583 1605.7485 1607.2385
1609.2674 1609.9105 1611.5455
2957.3751 3029.1481 3063.7532
3063.9039 3092.8822 3094.3395
3095.0488 3095.3119 3097.3856
3097.5813 3098.1819 3103.6141
3105.7039 3106.4012 3107.1419
3107.4515 3107.8971 3108.6871
3111.0629 3112.2152 3113.3486
3114.6559 3115.5814 3117.0656
3119.6504 3119.9350 3120.6321
3120.9096 3121.2810 3122.3502
3126.2331 3127.8771 3131.1566
3131.3977 3135.9977 3143.1747
3147.3416 3156.9691 3157.5090

Int B iso2 S=5/2

-14.9259 12.2770 23.0646
26.5505 35.0139 39.4483
44.4157 47.1175 48.5732
50.5814 53.4822 56.1746
60.3851 63.0286 67.6146
71.7240 75.2999 80.4263
85.2790 89.2971 93.8146
98.9879 110.2631 117.6744
124.3391 126.1983 128.8611
141.6578 169.3078 174.6693
180.0461 199.9155 205.5576
214.9623 218.0999 235.1579
241.3885 250.4612 251.2069
262.5647 269.7213 273.6854
281.2539 292.8532 303.4517
307.4090 311.8591 318.6025
334.7565 343.1433 348.1373
362.8221 372.5323 392.7758
396.1668 402.1933 403.4256
405.2876 405.7471 409.6774
412.9566 418.5024 422.4653
438.0278 464.1923 476.6691

485.0535	498.4177	500.5269
505.7518	516.4287	518.0595
527.8427	540.0076	544.3253
552.4837	585.3224	610.4575
611.6424	611.9119	612.3798
614.7730	623.7318	626.0762
627.9591	630.1483	631.7948
634.7155	636.5929	648.7185
662.8649	693.4111	694.3375
698.1849	698.2790	706.4375
711.8030	716.9294	724.9543
732.2638	747.4146	748.8683
751.4760	755.3003	758.8071
761.4830	772.7991	779.7898
783.1219	786.6034	791.3660
803.0299	816.2869	829.2502
831.6662	833.4221	839.6365
845.7081	847.3132	866.5000
867.6904	888.8498	889.3063
903.8965	905.2448	927.6920
928.2095	929.2965	930.1199
937.9180	943.8357	945.1133
948.8608	955.3122	956.6321
957.8514	961.3960	962.8447
964.1803	967.7791	977.4453
979.0566	979.8105	980.2523
981.0512	984.5168	988.2485
988.6667	989.0370	991.6470
995.0052	997.5450	1000.7915
1007.4279	1018.4809	1022.2343
1023.0423	1025.0024	1025.1699
1027.0264	1036.8663	1044.8993
1050.6938	1056.9555	1071.8503
1073.9571	1079.8601	1084.0757
1089.1989	1090.8928	1092.3803
1095.3420	1100.1052	1132.2015
1133.9104	1133.9920	1135.6858
1138.0430	1141.7680	1144.1510
1148.0778	1148.6911	1152.0565
1153.7156	1154.3437	1161.0351
1163.4237	1173.7622	1181.4677
1184.6390	1186.0023	1190.4217
1206.9801	1241.1850	1243.9518
1262.5221	1264.5590	1272.3137
1274.3239	1281.3500	1282.1117
1282.6731	1292.3181	1323.5941
1341.9886	1342.2716	1348.8900
1358.7975	1366.6321	1369.8914
1371.2654	1372.0705	1392.7232
1395.8632	1408.5401	1420.2714
1422.7970	1426.4621	1427.2771
1429.2884	1432.1669	1432.7684
1454.4213	1465.8852	1470.1631
1471.8771	1473.1578	1473.6149
1475.0266	1499.8196	1545.6244
1574.4289	1576.1093	1580.7852
1588.3019	1590.0002	1593.0931
1595.5598	1602.2068	1603.5122
1604.2881	1604.8451	1606.5229
1607.6069	1609.5823	1612.3590
2953.5818	3026.8775	3046.9249
3063.6905	3092.1705	3093.8768
3094.9128	3096.0313	3097.0041
3097.2680	3097.8719	3101.9081
3105.4154	3107.0574	3107.2147
3107.4004	3107.6100	3110.1392
3110.2193	3111.0769	3114.0306
3115.9627	3116.4832	3117.1684
3119.0488	3120.0319	3120.1372
3120.2909	3121.8083	3124.1547
3127.3039	3128.6429	3130.6251
3130.6863	3131.7527	3139.3644
3139.5391	3141.6803	3155.0622

Int C iso1a S=3/2

-4.4611	5.0546	14.7133
15.9950	26.1974	30.1711
36.6264	39.3213	42.3052
44.9154	47.8405	48.5408
53.6823	59.3874	65.1412
65.9892	70.7049	72.6267
74.2246	78.4182	81.6789
89.6620	91.4298	95.5380
101.4369	109.5684	114.4676
116.6281	124.3799	131.9469
133.7351	143.4105	163.6444
170.1616	175.6536	181.5282
198.4906	205.5326	208.8194
215.5022	232.7235	234.8002
239.3250	245.7938	249.5383
262.5417	265.3088	270.6574
278.4570	284.0140	285.3867
289.0541	300.9034	309.2902
314.9967	320.8877	332.4242
343.8948	347.0997	361.6988
367.8223	383.9586	400.5913
401.9481	404.9762	405.3518
406.1402	410.3368	415.6508
418.6537	422.0903	432.0650
442.0292	456.1123	467.2943
475.0742	477.4924	481.2878
496.0432	506.3904	508.8703
517.5406	518.2325	535.4748
539.5775	546.9474	551.9164
580.6774	607.0926	611.7384
611.9476	613.3442	614.1741
616.3601	620.4543	623.7700
625.6059	631.1408	632.7624
635.1942	637.7555	645.0608
661.7576	663.5443	672.7646
693.6757	694.9240	695.7520

697.4004 700.6685 702.2586
707.2225 720.2305 729.9689
735.3641 737.3757 741.7032
747.3032 750.5073 752.5953
758.3949 761.2531 763.6262
779.5491 785.9940 797.9635
799.0214 804.7806 809.7633
818.2706 821.6941 828.7136
829.6546 833.0461 835.0599
838.4863 842.4003 854.7330
867.2922 877.0034 879.5993
886.1384 899.9846 912.8410
919.6068 921.1709 925.4846
926.7596 930.3483 939.6637
941.1438 941.7355 944.5380
952.7658 954.3356 954.5995
959.6959 962.0366 963.6609
965.3198 967.8671 968.2093
971.3153 974.5412 977.4346
978.0132 981.0271 981.9908
984.7254 985.2188 985.9218
986.7574 987.7013 989.1842
990.0412 1003.6547 1004.8509
1011.2870 1013.6283 1022.4084
1024.8878 1024.9451 1026.2829
1026.6665 1043.7731 1050.2824
1056.2539 1059.9364 1063.8894
1072.5640 1073.4230 1082.2096
1086.5506 1089.0166 1090.4185
1092.5311 1095.7579 1097.2820
1102.4403 1110.0311 1130.6644
1131.0273 1133.1587 1133.7565
1135.9881 1137.5978 1141.5429
1145.5409 1146.0881 1146.3496
1150.6274 1152.1125 1153.9977
1154.8218 1155.9618 1157.5527
1161.6440 1174.0894 1187.6630
1196.5133 1199.9257 1207.6322
1210.5443 1229.1470 1239.0358
1241.0490 1261.5563 1266.0203
1266.3756 1275.5833 1278.6886
1282.2426 1283.4994 1285.2964
1288.6127 1293.2696 1328.2832
1342.3792 1342.9259 1350.3840
1357.6111 1357.9204 1364.1038
1368.8102 1369.5418 1370.0808
1380.2891 1393.3657 1397.2487
1405.9205 1407.1364 1416.6012
1421.3501 1426.5680 1427.0215
1429.3049 1432.2491 1433.5705
1441.1785 1456.2917 1468.6431
1471.3650 1472.3169 1472.6022
1472.7959 1473.9884 1479.7441
1505.9603 1543.9518 1555.8838
1574.9020 1576.9352 1578.5906
1581.2887 1586.4793 1590.6545
1591.3859 1591.6690 1605.3626
1605.8582 1606.2899 1607.2608
1607.5240 1610.6110 1611.9011
1618.0875 2942.7012 2958.0757
2994.1902 3031.9160 3061.2176
3063.2056 3091.3513 3094.4781
3094.7602 3096.4447 3096.4939
3097.1590 3097.8828 3098.5625
3099.0936 3102.2099 3102.9480
3103.5771 3104.0083 3106.3016
3107.9004 3108.8450 3109.6580
3111.5265 3111.6831 3113.5537
3114.2561 3114.5699 3116.9509
3117.2125 3117.4266 3120.3254
3120.3617 3120.7503 3121.0011
3122.7513 3123.8497 3124.7227
3125.0135 3128.4742 3130.2568
3130.7945 3133.2853 3137.9736
3139.8237 3142.6085 3154.4188

Int C iso1b S=3/2

-7.6838 13.6507 23.8254
25.2974 30.4391 32.7393
36.6828 39.2559 43.4991
46.1797 52.2141 52.7570
58.0075 61.9496 63.7841
66.5903 72.6008 76.7985
77.5251 81.2287 85.3069
94.9436 100.0791 102.5874
110.0862 111.4788 112.5992
118.5347 128.0105 132.1282
140.0622 144.8797 160.3925
169.9012 172.3720 178.6633
194.1031 197.3130 210.6273
222.6882 227.9382 235.7959
242.9158 248.1656 250.8691
261.6103 265.2293 271.0539
281.5054 284.2947 287.4344
294.5784 301.1688 311.4364
315.1142 320.6035 333.4529
342.3693 346.0625 363.2036
373.0157 386.7237 397.6477
402.2893 403.3982 409.2471
409.7859 411.4053 414.7787
417.4565 419.7300 429.0973
432.8620 448.6672 466.5719
474.1220 475.2869 481.1483
494.9406 503.1237 504.5233
518.9644 520.2752 535.4176
544.9819 551.7859 558.7793
581.2857 602.5939 607.6986
611.6381 612.3571 613.3513
613.5717 616.4264 623.7860
625.9914 629.4493 631.5931
632.9872 635.2926 637.8722

660.3437	663.2658	670.1415
682.4488	690.6803	694.0042
696.4741	700.4295	704.4846
706.4484	722.0154	731.3388
736.2381	741.0822	745.8536
747.3731	749.3813	752.8070
757.6184	759.4806	764.2969
780.0440	785.1633	794.9251
798.4722	802.7011	809.6123
813.4597	823.0077	824.3142
828.6903	832.7259	837.0247
840.5475	841.4019	855.0124
867.3077	874.9098	879.1442
885.9467	899.7858	911.8022
918.2728	919.1488	924.4951
926.6803	932.3631	934.2062
940.1638	942.1767	944.7680
947.3599	954.1488	955.3823
956.0918	963.3078	964.4738
966.5336	967.8285	970.3989
972.4849	974.6153	974.8852
977.5801	979.1481	981.7269
983.2030	985.2975	986.9194
987.9015	988.5889	990.1001
990.4573	1004.5077	1005.0118
1010.9879	1016.2863	1022.9226
1024.7799	1025.0915	1026.7817
1028.7318	1045.8614	1048.9870
1055.6553	1059.8697	1064.0008
1072.2680	1075.2384	1082.4194
1085.3618	1088.8032	1090.2365
1091.8703	1092.6417	1097.2625
1102.0076	1128.9075	1130.9423
1133.1644	1133.5046	1133.6367
1136.5224	1138.0538	1140.3614
1145.3312	1147.3782	1149.5599
1150.4174	1152.2489	1153.4121
1154.4305	1154.7874	1159.9272
1161.7364	1173.9059	1183.5054
1195.6023	1199.6865	1207.3737
1215.7611	1231.5842	1238.4287
1241.0790	1261.9193	1263.9403
1267.6815	1275.6523	1278.5243
1285.4970	1287.7076	1287.8717
1288.6031	1296.7482	1329.4374
1341.8848	1344.0628	1351.0464
1357.9150	1360.3846	1364.1821
1366.5405	1369.3891	1370.2353
1371.3866	1393.1431	1398.0716
1402.2470	1406.4087	1417.1756
1421.3146	1426.2055	1427.1938
1428.2614	1432.7075	1433.4505
1442.0509	1456.9040	1468.9223
1471.8805	1472.2197	1472.3889
1472.9268	1474.7916	1477.7539
1508.5141	1547.0386	1558.3955
1575.7118	1577.4423	1580.3666
1580.9037	1585.9708	1591.0611
1591.1918	1593.0493	1604.9185
1606.0942	1606.2838	1607.1846
1607.6562	1610.5063	1612.7674
1620.4682	2893.8348	2958.7503
3026.3753	3032.3904	3059.9609
3064.0443	3091.5645	3094.2309
3094.8759	3096.0773	3096.8206
3097.9168	3098.0146	3098.1526
3100.6051	3101.5666	3102.9186
3104.2354	3106.1426	3106.5909
3107.8569	3110.2875	3111.3183
3114.6552	3115.0210	3117.2805
3118.1216	3118.1732	3118.3299
3118.5638	3120.2850	3120.6530
3121.3484	3121.5231	3123.1182
3123.6292	3127.5085	3128.2547
3129.4151	3130.9492	3130.9631
3131.5386	3133.4164	3136.0256
3143.8525	3146.7165	3155.1453

Int C iso2a S=3/2

-7.3625	20.4306	21.4539
22.4958	30.5327	34.2613
37.9941	40.7672	43.0434
47.7323	50.6540	54.2912
58.6178	60.6943	66.9626
70.6625	74.5099	76.9182
82.0120	84.0583	88.5189
90.7662	97.1670	98.5188
102.2508	108.5759	117.5801
121.7176	125.1011	126.2419
135.6187	143.2033	151.0148
163.9294	170.8527	184.4249
189.4073	194.4994	207.5267
218.3765	225.7421	231.7888
236.0845	254.5976	258.6878
267.3093	271.5194	273.9712
279.6057	286.5920	290.0544
303.6286	306.4902	314.9004
323.4074	327.3479	332.3445
340.4341	345.7004	350.7582
361.7743	381.1469	396.7595
400.5992	401.3955	406.6855
407.7646	413.0792	413.3024
417.3096	420.6741	436.0773
437.0018	463.3022	464.9424
474.6846	478.4341	483.0121
487.7876	506.7162	512.3961
515.7531	525.3885	532.5738
540.6214	541.7758	552.4365
568.2277	576.9748	605.9258
611.1549	613.2620	613.8854
614.7337	615.4463	625.2503

625.7315	628.3939	632.7252
633.6337	637.3658	643.2112
657.8643	664.2567	670.9108
681.7924	687.7828	695.3638
697.1481	698.0773	703.3139
706.5987	708.0165	716.3334
728.4005	735.1160	738.6518
750.0104	751.2222	753.8340
762.7722	763.6986	768.5239
777.6119	780.3745	788.2432
795.0429	801.4405	805.2742
808.5188	817.1460	826.0605
827.9542	832.2081	833.4255
836.1990	842.9629	861.1997
866.5450	881.5980	889.3984
891.1771	905.3234	909.3895
915.5281	918.0304	922.1241
927.6569	929.4550	937.1514
943.4040	947.9591	951.4317
952.2001	952.7492	956.2576
958.5888	959.4674	962.0420
964.3174	965.6231	967.8571
971.0059	975.8824	977.1404
977.9055	979.9914	982.7976
984.4960	987.2627	987.6895
988.5564	989.1628	991.8547
992.4340	998.3383	1004.9779
1007.9899	1014.9158	1023.1682
1025.5160	1026.2577	1028.2728
1030.9034	1034.2677	1042.7084
1047.8285	1052.2153	1059.1155
1062.2960	1074.1956	1075.0702
1077.9788	1081.4392	1086.4599
1089.8088	1092.2021	1098.2530
1099.1642	1101.8703	1131.6709
1133.4094	1135.4635	1135.8089
1137.1964	1138.6350	1139.2983
1145.0882	1146.7853	1150.1654
1152.7648	1154.5016	1154.7976
1157.0767	1160.6520	1161.8722
1166.5297	1180.0590	1185.8339
1199.5780	1205.0519	1206.9937
1220.2275	1235.9628	1238.8033
1243.2113	1263.0432	1263.4074
1269.8428	1278.4970	1281.2143
1284.9438	1286.3277	1288.9267
1289.6883	1304.0201	1327.6302
1342.1773	1345.9893	1349.3721
1359.0154	1360.5801	1366.8530
1369.6176	1371.4278	1371.8325
1391.8620	1396.7175	1400.6100
1405.0977	1409.0608	1418.6406
1418.9555	1422.0124	1428.4079
1428.7308	1434.0835	1434.8984
1440.6231	1460.0302	1470.3794
1470.6626	1471.9125	1473.6716
1474.1055	1474.7082	1476.4877
1510.6955	1547.9623	1560.0790
1575.5816	1575.7058	1577.9860
1579.1574	1587.4009	1591.0060
1592.1894	1594.7077	1601.3681
1605.7469	1605.9987	1607.8646
1608.2693	1610.3114	1611.0831
1621.1445	2950.3861	2957.9007
3031.5008	3031.7705	3064.2573
3075.7294	3091.1731	3092.3922
3095.3254	3095.9124	3096.4098
3097.0844	3097.1529	3100.9260
3101.7910	3102.0667	3102.8582
3103.2333	3103.3013	3105.6225
3105.7983	3106.9974	3107.8917
3110.4328	3110.6899	3111.6479
3112.1555	3114.4184	3115.4943
3115.8160	3118.1459	3118.2802
3118.6531	3120.3957	3121.6251
3121.9512	3123.4088	3123.6539
3125.0666	3127.9212	3127.9508
3129.3804	3130.9986	3138.1560
3144.5072	3146.7210	3158.7581

Int C iso2b S=3/2

8.6085	14.8237	20.6804
23.8940	31.0649	32.5149
36.1309	40.3469	42.8916
44.2007	46.6075	56.1127
57.5264	59.9307	60.7381
70.0151	72.9029	79.3519
82.9188	83.8506	87.3698
90.2267	94.7377	96.3417
103.0703	113.1489	115.1325
120.8997	123.6552	127.0140
132.3057	138.3413	146.0631
153.6772	167.3753	181.6321
186.4312	194.8638	210.0294
215.3472	224.8631	231.2096
234.1757	238.0952	256.1380
260.5477	263.0326	267.1329
273.4943	273.7168	285.6100
291.5906	303.6418	312.1116
318.7464	323.4894	332.4441
341.1830	345.5484	358.4210
362.7329	379.2549	398.5184
401.8573	402.0743	408.5818
409.7477	413.6398	414.9796
415.4984	420.0524	426.5714
436.4389	451.5228	462.8240
473.2701	475.4534	478.7046
486.4941	510.4880	512.2422
515.9039	520.7566	531.7922
540.6575	552.1206	559.9791
576.7239	593.4199	606.8567

611.0618	612.9320	613.5747
614.3226	615.3412	624.5413
625.5821	629.4408	632.6221
634.5896	637.3988	649.3598
658.8531	664.6167	671.8261
680.6595	688.1462	695.9289
697.2909	699.2414	703.1025
707.3400	713.1055	716.8431
729.2376	734.5614	738.3566
750.3703	751.6181	753.0638
755.6195	763.2804	768.0212
777.8566	785.6816	794.7162
802.8989	805.4377	808.4293
815.4209	820.7930	826.6132
828.9319	831.6594	834.0315
838.0988	845.5238	860.7632
866.4870	874.8784	889.4924
891.0814	905.2935	907.0004
916.2807	918.8921	922.3651
928.2719	929.5758	933.7255
936.5480	942.9066	947.4255
951.4442	952.7090	955.3819
958.5023	962.4038	963.2228
967.3745	968.3327	968.3823
969.1932	975.2161	975.6772
977.8710	978.7627	981.5356
984.9843	986.9584	987.8479
988.6247	990.1593	996.0484
999.7021	1005.0800	1008.3081
1013.1607	1023.4681	1026.0637
1026.9953	1027.6116	1029.7388
1036.0470	1042.7855	1043.2664
1049.4109	1052.6966	1061.9907
1071.3761	1074.2647	1075.1764
1075.6820	1081.7940	1086.8473
1088.0006	1098.4750	1099.1362
1100.0746	1104.9630	1130.2766
1134.0792	1135.7544	1136.1616
1136.6555	1138.8821	1139.0442
1146.2090	1147.4410	1147.7036
1149.6991	1153.0235	1154.8313
1155.9541	1159.7701	1160.1387
1165.7385	1179.7411	1191.7868
1196.8531	1204.3446	1206.4596
1210.5305	1235.7104	1239.2684
1242.8431	1262.8147	1262.9912
1270.8521	1279.1732	1279.4788
1281.8018	1284.7949	1288.7398
1288.9160	1291.5003	1327.6650
1342.9714	1345.4295	1349.3113
1357.8016	1359.0080	1366.8076
1369.0352	1370.8793	1371.3400
1372.1944	1391.6348	1397.3440
1401.3676	1410.5544	1418.0854
1418.9711	1422.8577	1428.0751
1428.9298	1433.8166	1434.1309
1439.8157	1459.4611	1470.3712
1471.4344	1472.2751	1473.3790
1474.1445	1474.8177	1477.0761
1510.3856	1544.5348	1559.4417
1575.4744	1577.7263	1578.1690
1578.3254	1587.5081	1590.9471
1592.2962	1594.8897	1602.3557
1605.5820	1606.0285	1607.8561
1608.2571	1610.1638	1611.0371
1620.9065	2873.9750	2957.8008
2986.3834	3033.0619	3064.8540
3079.8180	3094.6018	3095.5050
3095.7216	3096.5245	3097.1776
3097.6780	3100.5152	3100.9914
3101.1459	3101.9992	3102.4893
3102.7394	3105.2489	3105.9630
3106.0620	3106.4997	3110.5204
3112.1977	3112.5729	3113.7004
3114.1907	3115.8045	3116.3073
3117.4067	3119.2767	3120.6542
3120.8082	3122.7876	3122.8385
3123.5184	3123.9998	3124.1415
3125.3308	3125.4070	3126.5604
3129.2739	3129.8472	3132.3589
3134.4409	3141.8067	3155.9410

Int C iso1a S=5/2

-8.2468	10.5213	14.6453
20.2289	23.4762	33.3565
37.0148	39.3759	40.5325
41.5243	46.0893	49.8913
52.3426	58.2171	63.4308
67.0338	67.4632	71.1272
73.0347	79.3099	83.0264
89.8696	90.6606	97.0359
99.1764	106.2276	112.6533
118.3944	124.0145	130.5601
135.8214	141.6744	164.2637
170.6357	173.9251	181.6077
199.0313	204.6849	207.6427
217.8818	223.2991	232.9108
237.7931	243.8007	246.8439
262.4970	265.5814	271.2698
275.7762	284.1400	286.3619
288.0960	300.8202	306.0311
312.7901	320.9632	330.5853
344.8507	347.5187	360.2559
365.1099	378.6364	399.1819
400.7917	403.9268	404.1000
405.2328	409.2478	413.6320
418.3829	422.7515	430.5701
437.7748	456.9771	467.2211
474.7399	478.0933	480.5401
495.4334	506.8768	509.1313
517.1288	519.1870	535.5059

538.8716	546.7564	552.2153
579.5981	606.4985	611.7157
611.8947	613.1198	614.1556
616.2234	620.0001	623.4639
626.0293	630.6795	632.7961
635.2427	637.0966	645.8713
660.9930	663.2157	671.5209
693.0325	695.1511	695.3573
699.3649	699.7461	706.7184
707.2497	718.6722	728.9385
736.2063	738.8747	741.5769
746.9454	750.7960	752.0512
760.3228	762.1160	763.8798
779.2627	785.7259	797.4888
798.3454	804.8542	811.8812
819.1154	820.7917	828.5991
832.1978	832.3192	834.7873
837.4855	842.0398	854.7674
867.1535	878.8919	882.8042
885.7955	898.6840	911.2613
919.1665	920.4861	926.1761
929.3588	931.4610	938.8407
941.0493	941.7918	944.4635
953.6100	953.7984	955.8140
959.4382	960.0767	962.7642
964.7639	967.6483	968.8657
973.2959	974.3551	975.4311
977.1946	980.1522	981.1755
982.4382	984.8532	985.2695
986.6520	987.5811	989.0619
989.9445	1003.2534	1004.5328
1011.3888	1013.3722	1022.2177
1024.6361	1024.7406	1026.1525
1026.6459	1043.0082	1050.9121
1057.7995	1061.8014	1063.6333
1072.2827	1073.2771	1083.8514
1085.9120	1089.3435	1092.0582
1093.3919	1094.4083	1096.4383
1101.8123	1119.4374	1130.5246
1131.4322	1132.9066	1133.3589
1135.7658	1137.4049	1141.8341
1145.0469	1145.4698	1147.3188
1150.4518	1151.7543	1153.1305
1154.4830	1155.1550	1158.1310
1161.6925	1172.6240	1186.8971
1196.6371	1200.0498	1207.6176
1213.3035	1226.0672	1238.9554
1241.0613	1260.0037	1263.8345
1266.1397	1275.3517	1278.2881
1283.2336	1285.8437	1286.1662
1288.0566	1296.8211	1328.1472
1342.0592	1343.1581	1350.2319
1357.6484	1359.6964	1364.0088
1368.4980	1369.5802	1370.3150
1389.2822	1393.6785	1397.2627
1406.6574	1410.2500	1415.6929
1421.3291	1426.4392	1426.7744
1429.3190	1432.0485	1433.2799
1442.1403	1456.2983	1468.5896
1471.0851	1472.0809	1472.2618
1472.9108	1473.2033	1483.3199
1504.9745	1544.9014	1554.7637
1574.8672	1576.6465	1579.1466
1581.0005	1585.9543	1590.6384
1591.2379	1591.6908	1605.2620
1606.1002	1606.1380	1607.0501
1607.6101	1610.3116	1611.5833
1617.1260	2927.5943	2957.8964
2999.6498	3031.7106	3060.9338
3062.8543	3094.4218	3094.5409
3094.8110	3096.1641	3096.4756
3097.2142	3097.7189	3098.6863
3101.0094	3101.9526	3102.9193
3103.5724	3103.7531	3105.7526
3107.7760	3109.5453	3110.7548
3111.3125	3112.8826	3113.6563
3116.1033	3116.3861	3117.0097
3118.1210	3119.9690	3120.1738
3120.6161	3121.2406	3123.4724
3123.5704	3124.4833	3124.6934
3125.0476	3126.8343	3127.7374
3128.4246	3130.0961	3137.7093
3138.4871	3144.1083	3154.5835

Int C iso1b S=5/2

-9.9704	13.4448	22.5506
26.1628	31.1236	35.4794
37.0273	38.7819	43.8475
44.2329	51.7240	53.0014
56.3784	61.9967	63.6918
66.5880	71.8648	76.3558
78.4988	80.7360	84.6431
94.1303	101.0304	102.9538
109.4123	110.4480	113.2255
118.6859	126.8030	131.6056
138.3956	143.9758	158.9150
169.5538	171.5840	178.3826
191.2492	196.4906	207.6527
215.4027	226.2361	233.5121
241.8792	247.1944	249.9185
260.6211	265.3962	269.9969
280.3567	283.3317	286.4227
294.5855	300.7471	311.0087
314.1857	320.5597	332.4420
343.0152	345.8908	363.7628
371.8647	385.0146	395.2845
401.5799	403.6671	408.9617
409.7956	410.6414	413.3697
417.4732	419.4148	427.5552
429.3953	448.3133	466.4541
472.5040	474.2713	479.5633

494.5289	502.6091	504.3330
518.1455	520.3321	535.3937
545.0579	551.6960	558.0067
581.2626	596.8694	607.3378
611.6569	612.2766	613.2773
613.5647	616.4261	623.5871
625.9903	629.5086	631.6072
632.7366	635.3664	637.6994
659.5397	662.7787	668.7368
689.2352	692.4644	693.9171
696.2979	700.7729	704.2443
706.3060	720.8896	731.0661
735.9621	740.7050	744.7080
747.2375	749.2686	753.0401
759.0005	760.4184	764.3481
779.9764	784.3476	794.6017
797.5308	802.7260	809.5318
817.2959	822.6897	824.8149
828.8330	833.0086	837.3424
840.4519	841.3321	854.9137
866.9933	876.8029	881.5904
885.5355	899.5372	911.8492
916.3085	918.7871	919.6859
925.7513	932.3558	933.6466
939.9228	942.2034	944.4496
950.1356	955.1231	955.4783
955.9435	963.4501	964.7068
966.5403	967.7835	970.8708
973.5487	974.3643	975.6039
977.3083	979.3461	981.6764
983.0821	985.3022	986.8888
987.9525	988.3505	990.1438
990.8793	1004.5171	1005.7006
1011.0047	1016.4012	1022.9097
1024.7177	1025.0331	1026.5171
1028.2529	1046.0003	1048.8985
1054.9435	1061.7081	1063.2746
1072.1884	1074.3904	1083.0194
1085.5942	1088.9721	1090.1466
1091.8571	1092.7045	1096.2973
1100.4192	1131.0369	1132.9335
1133.4988	1133.5987	1133.9070
1136.4010	1138.0705	1139.9042
1144.8569	1146.5101	1149.2735
1150.9703	1151.9255	1152.8349
1154.4216	1154.8322	1159.2795
1161.8062	1172.9111	1181.3877
1197.1321	1199.8790	1207.3691
1208.8540	1229.5981	1237.7980
1241.2215	1261.9066	1263.7063
1267.5995	1275.0102	1278.3298
1285.5949	1286.2631	1287.3673
1287.8609	1296.7973	1329.6192
1341.8409	1344.0280	1351.0499
1357.4255	1358.0409	1361.5045
1364.2431	1369.3755	1369.8138
1370.9705	1393.2587	1398.2965
1404.5657	1406.3768	1417.0967
1421.4608	1426.1238	1427.1970
1428.1472	1432.5148	1433.3887
1442.6045	1457.0018	1468.7054
1471.6214	1472.0521	1472.2461
1472.8458	1474.3500	1478.9840
1508.5736	1547.8515	1558.3265
1575.7301	1577.3539	1580.8429
1581.0790	1585.8741	1590.8960
1591.0391	1592.6923	1604.8886
1606.1873	1606.2638	1607.0346
1607.2207	1610.4185	1612.4409
1620.5758	2901.7750	2958.7006
3032.4779	3033.2056	3063.8679
3067.1072	3093.0441	3094.0382
3094.6722	3095.8776	3096.4541
3097.4447	3097.6827	3097.7189
3101.5430	3102.4266	3102.8317
3104.0149	3105.9212	3106.3305
3107.6231	3109.6227	3111.2981
3114.2782	3114.8303	3116.0966
3117.5063	3117.9815	3118.7267
3118.8879	3120.0234	3120.4184
3121.0967	3121.3496	3122.9698
3125.7965	3126.4261	3127.9791
3130.1125	3130.3978	3131.1129
3131.6527	3133.7607	3135.1303
3143.9063	3146.4562	3154.8160

Int C iso2a S=5/2

9.1170	16.2714	23.8487
27.4567	28.5208	34.3794
37.1893	43.1784	46.0528
49.7966	58.0817	58.6244
61.8772	67.2577	70.1405
73.4483	75.7536	77.9017
81.2942	84.7667	86.7862
91.1640	93.9955	99.4684
104.8881	111.5124	118.7229
120.2254	126.6591	127.9883
132.0111	144.4067	147.1840
157.9054	170.1274	179.6029
190.2409	198.8001	209.5166
214.5607	229.0302	229.3458
235.0404	241.1087	255.6552
262.6782	263.9659	268.8722
271.3950	274.6433	282.9157
287.1790	300.2102	305.0029
320.4999	323.5916	325.5898
337.4582	347.6808	353.9903
362.2887	374.1729	392.4228
398.7786	401.6807	405.8231
406.9290	408.4296	412.1009
412.4848	419.4539	434.2377

438.7362	463.3438	473.0111
473.8699	474.4903	482.3639
485.3045	508.3546	512.2636
515.4299	517.3582	531.8667
540.8385	551.6359	553.6425
566.2070	576.7453	605.5948
611.0067	612.8407	613.4857
614.6445	615.5850	625.2589
625.6929	630.3136	631.1091
634.7514	638.7610	643.3788
654.5130	662.2983	670.9235
681.6297	686.6574	692.8377
698.0627	699.1312	700.7274
707.6614	714.6651	729.2088
734.2094	735.4213	739.0548
750.3573	751.2099	755.1680
760.7570	763.9701	767.8988
779.8420	784.1851	787.7006
789.0109	797.8235	801.9811
814.6880	816.0326	823.8265
827.4944	830.3821	833.6655
837.2603	843.7273	858.8650
866.4084	885.8431	889.3250
895.1012	902.3390	906.0497
918.1506	918.8241	922.1569
927.5812	929.4937	936.8226
940.6845	941.9183	947.1072
952.6937	955.1801	956.3158
956.6345	962.3210	962.8474
966.8872	967.8881	969.0835
974.9995	975.9479	977.2931
977.8140	979.7885	982.7802
983.2252	987.3166	988.1140
989.5610	990.2503	992.1495
1001.6839	1003.1541	1006.4223
1008.3915	1017.4037	1023.9672
1025.6358	1025.9921	1026.2542
1028.1647	1037.9776	1041.9789
1050.3176	1052.7442	1056.3277
1071.1174	1071.4655	1073.5856
1075.1622	1078.6800	1088.7377
1089.6422	1092.6303	1098.5781
1099.2831	1104.9669	1131.3860
1132.7127	1133.5197	1134.9088
1135.6720	1138.8317	1139.7128
1146.8593	1147.3585	1151.2367
1151.4253	1154.0564	1154.4761
1156.8168	1157.5064	1160.7231
1163.6357	1171.1451	1190.4427
1205.4474	1206.3013	1207.1893
1213.2146	1235.8508	1236.4690
1240.7890	1263.8966	1264.8377
1273.6020	1277.4964	1281.9511
1284.6477	1286.4984	1289.4340
1295.8651	1317.3648	1330.6601
1342.2406	1349.2056	1350.0811
1359.5741	1364.1424	1367.0889
1369.0129	1371.5455	1373.1957
1391.8125	1392.5900	1402.0492
1403.3564	1409.0625	1418.8533
1419.2545	1423.3255	1427.6597
1430.3299	1433.2943	1434.1136
1441.3293	1459.5744	1469.8084
1471.8526	1472.6454	1473.4757
1473.6035	1474.3247	1478.9324
1513.0266	1550.3634	1561.2889
1577.1753	1578.0906	1578.1534
1580.6891	1587.0611	1591.9788
1592.3723	1594.6535	1602.9346
1605.3039	1606.1573	1608.1634
1608.4189	1610.6807	1611.6577
1624.7773	2922.9371	2958.8406
2990.4225	3032.8293	3065.6608
3084.1176	3091.5587	3092.8386
3095.8311	3096.1387	3097.0396
3100.2005	3100.4138	3100.6583
3101.2870	3102.4779	3103.6473
3104.0800	3105.7974	3105.8027
3105.9019	3106.2333	3107.5944
3111.9481	3112.1439	3114.0883
3114.4752	3115.9021	3117.3175
3118.2027	3120.7046	3120.8740
3122.3642	3122.4338	3123.7634
3124.1228	3125.8223	3128.6078
3129.3744	3130.7256	3133.4373
3135.5236	3136.9241	3137.6185
3139.9816	3144.6874	3151.9967

Int C iso2b S=5/2

7.3912	12.6448	20.9493
24.4065	31.5932	32.8288
34.9635	39.5662	42.8470
43.4514	46.4797	56.0217
57.0887	60.1930	61.2808
70.6039	73.4357	78.9148
83.0969	84.1097	87.9216
90.2204	94.8096	96.5693
102.8519	113.6597	114.6490
121.4648	122.8069	127.1422
132.0731	137.9840	146.0088
153.8994	167.8539	180.9427
186.7547	195.5254	211.4619
215.5798	225.5728	228.3443
232.5126	237.0874	255.5669
259.9290	262.6446	266.2321
272.9150	273.0944	285.3500
291.1539	303.5303	312.0743
317.8649	323.3921	331.1152
340.5614	346.0892	357.4929
362.6189	377.5514	398.2466
401.9610	402.2334	408.7105

409.7785	414.1626	414.6775
415.1161	420.0452	424.9472
436.8073	448.3451	462.7735
473.9175	475.3227	478.0901
486.3008	510.5616	512.2902
516.1922	520.0401	531.6136
540.5978	552.0479	559.9453
576.6097	593.7069	606.9117
611.0670	612.9891	613.5915
614.3629	615.3540	624.5999
625.4876	629.4293	632.4987
634.7202	637.2766	650.8964
658.7164	664.3314	671.6446
682.4130	690.1347	695.5880
697.3385	699.9831	702.9146
707.7727	714.1096	716.6420
729.0355	734.3033	738.3395
750.3866	751.6201	755.0518
755.9130	763.3182	767.9445
777.9919	784.6883	794.3400
802.4361	805.3524	808.9902
815.2948	821.2755	826.8385
828.9652	831.8517	834.4897
838.2847	845.0260	860.6964
866.4151	877.4121	889.2059
890.6937	904.9605	906.7755
916.3629	919.1035	921.8352
926.3264	928.9546	929.4422
936.2323	942.8554	947.2443
952.2135	952.7226	954.8571
958.4885	962.4725	963.3935
967.3490	969.0533	969.0964
970.1536	974.9646	975.5351
977.7697	978.9511	981.5508
984.6533	986.9847	987.8321
988.8046	990.2085	995.3097
999.8655	1005.1497	1008.2952
1013.4718	1023.4601	1026.0653
1026.9806	1027.4858	1029.5417
1036.1587	1040.2079	1043.5539
1049.4603	1052.8371	1060.6128
1073.3697	1074.4175	1074.9772
1075.3556	1080.5548	1086.8341
1087.9870	1098.5031	1098.7030
1103.8781	1107.0445	1130.6441
1134.0401	1135.6372	1136.0566
1136.5192	1138.8838	1139.0257
1146.2325	1147.5841	1148.1100
1149.6646	1152.9381	1154.6094
1155.9176	1159.4861	1159.8480
1165.5892	1179.1450	1191.2564
1195.8626	1204.3092	1205.9642
1206.4950	1235.6431	1239.2548
1241.8341	1262.6565	1262.7958
1270.9978	1277.2048	1279.2260
1282.0952	1285.0465	1288.4835
1288.7241	1291.5771	1327.9175
1343.0684	1345.6037	1349.3555
1358.4159	1359.0216	1364.9650
1366.9115	1368.9801	1371.3669
1372.2519	1391.5370	1397.6991
1402.2835	1410.6114	1418.0128
1418.9465	1422.8939	1427.9753
1429.0355	1433.6843	1433.9776
1440.1837	1459.4085	1470.3057
1471.6074	1472.2761	1473.2661
1474.0849	1474.8074	1477.5394
1510.4683	1545.4222	1559.4504
1575.4910	1577.7796	1578.6253
1578.7517	1587.4727	1591.0254
1592.2419	1594.8108	1602.2945
1605.4645	1605.9504	1607.8594
1608.2114	1610.1961	1610.9505
1621.0889	2874.0759	2957.8230
2985.2948	3033.1668	3064.9331
3078.3752	3095.1018	3095.5622
3095.5681	3096.4250	3097.0522
3097.7890	3100.0713	3100.3649
3101.1038	3103.0440	3103.4155
3105.2221	3105.9145	3105.9535
3106.4355	3106.8808	3110.2619
3112.0558	3112.5875	3114.1235
3114.4727	3115.8644	3116.2388
3118.8605	3119.3680	3120.6205
3121.7575	3121.9156	3123.4009
3123.6863	3123.9964	3125.3917
3125.6081	3126.1265	3126.2046
3130.5264	3130.6652	3132.6176
3134.9977	3142.0709	3154.6496

Int D iso1a S=3/2

-5.4071	12.0778	21.9999
24.6382	29.3044	32.7460
37.5110	42.4327	47.4773
50.9842	52.8848	58.9597
60.4300	64.0351	66.0245
68.2176	73.6035	77.9584
80.9224	82.2277	85.2199
87.6074	98.9376	99.7069
103.2514	104.8072	113.8755
116.5803	125.0004	126.0972
138.1954	141.1871	145.9867
173.6766	183.8097	186.2692
191.8846	201.4178	206.9283
216.8254	222.8654	228.8396
234.5445	242.2457	248.6101
254.3359	264.1041	268.5898
278.5888	279.4784	287.6961
298.0900	304.7381	316.1309
321.0595	326.3642	327.4597
334.7425	339.6930	365.2543

366.0349	371.1329	380.6421
395.7351	399.8300	401.7253
404.1347	406.2528	406.9009
411.7956	412.8377	416.7352
434.3860	466.6689	467.6074
469.6858	476.1739	484.1763
491.4150	498.4851	509.0447
526.5262	533.7480	541.8548
548.2508	550.6801	554.5872
576.6511	596.4318	610.6787
611.4219	612.1156	613.0637
613.2563	613.7595	616.1733
624.0770	625.4008	628.4625
633.0403	634.9313	635.3294
650.6871	659.7782	667.3792
696.2681	697.6017	698.5447
699.3902	701.9902	703.7664
707.9555	724.9296	727.1325
734.2763	742.8314	749.4245
750.0709	752.4596	753.6362
761.6585	765.8036	767.2780
780.3641	788.3597	794.2656
798.1001	800.7926	806.8033
822.0183	826.8589	827.5929
832.5206	833.0470	834.3253
836.5338	850.8123	865.3580
867.1099	884.6909	886.7607
895.1610	905.7037	909.8202
910.2239	918.1222	921.5572
923.3120	930.2704	932.1545
941.1687	944.2682	945.7796
949.0751	956.0275	959.0712
961.9481	963.2988	963.9518
965.2128	966.8204	972.5470
976.2214	977.1302	978.8020
981.7328	983.2116	984.5178
986.7771	987.7484	987.8516
987.9118	988.0735	990.4261
990.5692	998.7582	999.1288
1004.3418	1008.6448	1021.0652
1023.3895	1023.8879	1024.6525
1026.6044	1028.0302	1031.7566
1035.9921	1042.5854	1050.4285
1056.8853	1058.7507	1063.6147
1072.1790	1073.6386	1074.1982
1076.4786	1084.5329	1088.1797
1092.4815	1097.1318	1101.1835
1104.1480	1133.5131	1134.2233
1134.5891	1135.3291	1136.1690
1138.3231	1141.4033	1142.5547
1146.6175	1150.3327	1154.6766
1155.3977	1156.2029	1156.6225
1157.3701	1160.7248	1168.5911
1171.1143	1180.3331	1187.3907
1190.6399	1200.8745	1206.6476
1236.9148	1240.0759	1265.9104
1266.1419	1270.9878	1279.6878
1284.5357	1285.6352	1288.9432
1289.5627	1290.2670	1329.5804
1339.0151	1344.9698	1345.4400
1352.3552	1360.6603	1366.6229
1368.2549	1368.9505	1371.6523
1378.2218	1393.2654	1403.2956
1404.1889	1408.8822	1420.2831
1420.6903	1423.0755	1429.2389
1429.5255	1430.8620	1433.1528
1442.5739	1458.5645	1469.0727
1470.5358	1472.5577	1473.7832
1473.8794	1477.1716	1484.5426
1508.2364	1553.7319	1575.3032
1578.5974	1583.8983	1588.2994
1591.0324	1591.9890	1593.4551
1594.2588	1604.2221	1605.0508
1605.1106	1605.7132	1607.8620
1609.8212	1611.2627	1614.9040
1618.8493	2953.4861	2999.9752
3029.6010	3061.5134	3092.1110
3092.8175	3093.8769	3094.8179
3095.0448	3096.5212	3096.7277
3098.5935	3099.0238	3099.3488
3101.1307	3101.5154	3102.8336
3103.3084	3104.9876	3105.7434
3107.7196	3109.1736	3109.5856
3110.9978	3111.3925	3112.4297
3112.6350	3113.4934	3113.7847
3117.1630	3117.6561	3118.9439
3119.7166	3120.3479	3121.4089
3122.2669	3122.6231	3124.6858
3125.6979	3125.9312	3128.1710
3129.7873	3130.9972	3135.5433
3136.9773	3140.7739	3163.4729

Int D iso1b S=3/2

-3.3972	8.0509	15.7939
24.9339	25.5492	32.5642
39.6653	44.9559	47.4207
51.9733	52.6402	55.2420
58.4561	61.6622	64.1967
65.5544	69.8145	73.3102
74.2815	79.7242	83.8137
87.2232	88.8934	93.0958
98.7522	105.7107	106.9020
107.0328	108.1482	121.7180
126.8243	131.4943	133.1804
142.1799	164.4057	168.9902
182.2581	186.7137	200.4155
207.7392	218.5422	224.5282
235.1975	240.4481	242.6718
254.0588	259.9278	267.2543
271.1319	279.3215	284.5741
285.3625	291.8972	298.3477

308.4473	328.0782	334.0759
345.1040	355.1456	359.5438
370.6635	385.1360	388.8741
400.0492	400.4698	401.9951
404.0016	404.1115	408.2049
409.0997	412.3584	415.6744
421.1906	424.5354	444.5214
465.7715	467.8567	487.4249
492.1208	504.5950	512.0966
514.7223	518.0908	528.5029
530.6988	543.0326	552.6473
573.6938	583.4223	592.4575
611.3978	611.5897	612.1281
613.0272	613.8196	616.0692
623.7623	628.9113	632.0941
633.2382	635.1679	638.6506
662.6994	666.4000	693.8448
698.8243	700.1276	701.1100
701.5120	703.5165	707.1384
720.8241	731.9102	736.4204
741.8097	747.9375	751.7349
753.4246	757.7917	761.3081
764.6828	767.5091	780.3310
790.7324	795.3880	797.9182
815.1418	824.5237	824.7297
827.0826	829.2006	831.8390
836.9958	838.1566	839.0595
848.1829	867.5671	869.1470
884.9671	890.8785	904.6702
905.2547	906.9787	919.3888
920.8036	927.2771	930.0180
934.6442	941.1628	945.0531
945.2610	945.8875	948.4430
959.2365	959.8876	961.5565
961.8820	962.8229	963.1001
966.6510	969.0399	974.6403
976.7235	977.0257	980.7823
981.8757	982.6730	984.7925
986.7737	987.1442	987.8268
988.3572	989.0096	990.4223
994.5888	996.1750	1004.0193
1011.0312	1020.7555	1022.7650
1024.4001	1025.7006	1025.9650
1027.2373	1027.7171	1038.4540
1046.9469	1049.1060	1050.1406
1060.3172	1069.2712	1072.5463
1074.5642	1079.9243	1085.7990
1087.9409	1090.7660	1091.9628
1094.6449	1098.8413	1102.5793
1130.9009	1132.4616	1134.2395
1136.9016	1137.8278	1138.1608
1139.5523	1142.8009	1147.2816
1149.0579	1149.7650	1155.0673
1156.2540	1157.5963	1159.8152
1163.8609	1164.6013	1178.8464
1180.7146	1187.1975	1197.3801
1197.7315	1205.4316	1239.8773
1241.1867	1242.9378	1260.2974
1264.6633	1268.5003	1276.8855
1281.4731	1281.6921	1285.2676
1288.4241	1290.1637	1325.4196
1342.2922	1342.9413	1345.8025
1348.3327	1355.7052	1368.7744
1368.9975	1369.4393	1370.6142
1380.4040	1389.2386	1396.5646
1407.5252	1418.4388	1420.7736
1424.1710	1425.2234	1426.8029
1430.8669	1431.4398	1431.8433
1442.4566	1456.5364	1465.7460
1466.6671	1471.8844	1472.0063
1472.7011	1474.3936	1484.9510
1505.5386	1553.2162	1573.9891
1576.3915	1577.2128	1590.1746
1590.2391	1590.2815	1590.6512
1593.2348	1598.8393	1605.2605
1605.8341	1606.1868	1606.6490
1609.8755	1611.1334	1612.7527
1616.9213	2957.5412	3029.3350
3030.1150	3039.9193	3043.4183
3063.2757	3094.2742	3094.5012
3095.9272	3095.9652	3096.3785
3096.4903	3096.7293	3097.2807
3099.5708	3103.4872	3104.9956
3105.4689	3105.8802	3106.8008
3107.9290	3109.0098	3110.1952
3111.1128	3113.1554	3115.3026
3116.2170	3116.6952	3117.0715
3117.5870	3117.7445	3121.4559
3121.5479	3121.9224	3122.4809
3122.9031	3122.9252	3123.2120
3124.6753	3126.9014	3128.5032
3129.6282	3131.5526	3132.2461
3136.0602	3142.7224	3142.8376

Int D iso2a S=3/2

18.2750	20.7387	24.9831
30.0362	36.7809	38.9703
44.3136	45.8409	50.3644
50.5295	55.4180	57.1561
58.5725	62.6907	67.3484
70.0105	72.5898	75.4282
77.9879	79.2592	87.2725
89.4652	91.3942	97.4245
101.0344	103.6202	106.2491
112.8586	116.2848	122.3460
128.8263	131.4260	138.6829
146.1871	158.6478	178.0568
181.3970	186.1771	192.1868
205.6696	219.4709	223.7556
230.8737	235.8833	239.7034
246.1451	249.7825	256.6653

268.3527	274.2263	281.7458
285.3848	288.1190	303.0110
307.6805	312.1887	329.6450
343.1042	346.9030	349.0773
369.1580	372.9105	383.8289
386.3808	398.8556	400.2110
405.6347	407.6184	409.2494
409.5834	413.0399	414.3633
424.0062	429.7128	446.5152
465.3255	466.7138	483.9161
500.8666	501.5308	507.4240
511.2189	516.3099	528.0257
539.5991	544.5318	553.1379
585.1521	585.9303	594.8815
611.7145	612.5256	612.7064
612.9845	613.7207	615.7605
622.5214	628.4266	630.8755
632.5571	633.4366	637.6832
653.2045	663.2043	688.6938
694.3700	698.6160	699.6708
705.6866	709.9473	710.6086
713.5278	726.7897	732.8194
737.6404	746.6378	749.8699
752.7190	755.7512	756.1859
761.6763	764.4905	778.8746
783.6779	789.0175	791.7868
795.1999	818.1941	831.3090
832.3791	833.4671	836.6583
840.0837	843.6669	848.8477
854.9385	866.7739	869.4496
885.2069	887.7275	900.0792
903.9862	905.3202	920.7418
921.2493	925.3571	927.0737
933.5288	936.8349	943.8847
945.6409	948.5285	950.7095
952.6633	956.9739	957.3668
959.9966	963.8914	965.2301
965.5357	969.9064	974.4398
977.4504	978.3783	979.4471
980.5503	985.2552	986.0265
987.0475	987.8658	989.1078
989.5692	990.9008	993.6523
996.1357	1004.6024	1004.8150
1009.7417	1011.3450	1022.4666
1023.7441	1024.2033	1024.3372
1025.0629	1029.8332	1037.5225
1043.2634	1046.0928	1048.2658
1052.4415	1058.6284	1070.4588
1070.7665	1074.6084	1077.8169
1079.8215	1084.7649	1090.4125
1092.3065	1095.7048	1100.0429
1123.3083	1133.0032	1133.2578
1133.7482	1134.8905	1138.2522
1140.2271	1141.3911	1143.7839
1148.6830	1150.0382	1152.1488
1154.1798	1156.1537	1157.0620
1157.1953	1166.8296	1173.0137
1178.0243	1185.5658	1189.3003
1196.4307	1207.6500	1234.3434
1241.7896	1243.1225	1262.0089
1266.0942	1272.9440	1273.6534
1281.3185	1282.5186	1285.6568
1289.1255	1292.5081	1329.1583
1341.9634	1345.0567	1352.9613
1355.5257	1356.3591	1366.7429
1368.3654	1369.5946	1370.8520
1380.9798	1387.6731	1396.0623
1406.5745	1418.1844	1421.7807
1422.8032	1426.6321	1427.7150
1429.8278	1430.0217	1432.5254
1443.1988	1456.5536	1466.1238
1468.2581	1469.3912	1471.9536
1472.7365	1476.4047	1485.0871
1504.4617	1553.7802	1574.8279
1577.7573	1578.2361	1588.7520
1589.6192	1592.4602	1593.3818
1594.1695	1597.0790	1603.9636
1604.8464	1606.0053	1606.5906
1609.4201	1610.0962	1612.3403
1615.7450	2956.1740	2992.0118
3021.3834	3030.0934	3050.4670
3068.3807	3084.6519	3092.8331
3094.4257	3095.5012	3096.3728
3096.7550	3097.2281	3097.5894
3097.9324	3105.1944	3105.5808
3106.3002	3106.8549	3106.8817
3109.1401	3110.3865	3111.3600
3111.7922	3113.8686	3114.1026
3114.7907	3115.4903	3119.0952
3119.3904	3120.0117	3120.6979
3121.2244	3121.5417	3123.1420
3125.9331	3127.7541	3128.4795
3129.0897	3131.4091	3134.8758
3135.9259	3139.0406	3145.4843
3148.9142	3149.8379	3151.1784

Int D iso2b S=3/2

0.6775	25.2362	27.3276
30.5174	37.8733	41.6412
44.0133	48.3029	50.7347
53.6283	58.7132	59.9605
61.5747	67.6934	70.8579
71.9999	77.1151	80.3232
81.6686	83.9511	87.6239
89.7932	93.9601	97.9935
105.7291	107.3483	108.1342
113.8679	115.5867	118.3517
122.8843	127.1676	137.9080
145.6652	155.4754	178.6858
185.1646	189.6848	206.8853
211.2166	218.9166	227.7032

234.7419	241.5298	245.1562
255.3279	258.1866	260.9267
273.8377	277.8928	284.8293
287.7274	296.2132	303.7262
307.7608	315.4173	329.6848
340.7918	348.2861	369.1957
373.0716	379.4111	384.2539
398.9947	401.4699	401.7301
404.3289	406.9522	409.9771
410.8800	416.7742	420.6066
423.1018	426.8543	431.7794
450.8733	466.5897	482.6499
499.5274	505.2027	511.8737
513.7086	519.3057	523.7558
533.3649	538.4457	553.4457
573.7214	586.5813	595.0610
611.8437	612.7384	612.8818
613.5232	613.8438	615.5964
624.1618	628.1444	632.1521
632.8395	635.3818	636.9303
655.0605	663.7005	689.4066
693.0425	696.2045	699.1851
705.5400	706.6771	712.3730
716.6187	727.5376	733.2644
748.0929	749.5379	749.8378
752.7076	757.2872	761.1820
763.6826	766.6390	781.5757
783.6393	788.8231	795.7813
801.0358	817.0680	828.2499
830.2482	834.1024	840.6537
842.1587	843.5627	849.3411
850.4693	867.2672	868.8594
887.7817	888.4788	905.2487
906.6994	907.3138	921.8077
923.7264	925.6293	930.3036
932.5431	935.4926	943.9984
944.4321	945.5405	948.3942
954.1521	955.3741	960.7154
961.4724	962.1832	964.9709
971.9419	972.8554	974.5750
976.6412	977.9598	979.7238
981.2231	982.4031	986.4365
987.5377	988.6915	988.7544
989.2874	990.1036	994.8643
1000.6038	1004.3332	1005.4304
1009.5506	1021.3070	1023.1518
1023.6992	1024.9641	1025.1692
1026.6954	1029.0228	1042.7123
1047.0774	1048.7969	1054.4175
1060.1004	1064.8561	1072.1180
1072.4622	1079.9115	1081.0852
1086.9895	1089.8210	1091.3311
1093.8024	1095.2659	1100.2417
1125.2929	1131.3460	1131.7610
1134.0538	1135.3741	1138.0633
1140.5309	1141.6728	1145.4874
1147.7091	1150.0465	1152.1416
1155.3714	1156.7152	1158.3270
1161.5386	1163.6215	1176.2200
1178.4621	1183.0113	1189.5338
1194.8493	1208.3016	1240.4495
1243.0059	1243.1872	1261.1314
1266.2763	1271.4280	1272.5422
1278.1800	1281.9861	1283.8588
1287.1508	1290.1305	1328.8420
1342.0022	1344.8698	1345.7165
1352.1419	1355.5391	1365.9630
1368.9022	1369.2307	1372.0564
1380.1665	1388.4414	1395.0354
1407.7945	1417.1540	1417.9638
1422.8453	1426.0311	1427.3008
1427.6451	1429.5823	1431.4108
1441.7988	1456.6016	1467.0428
1468.0683	1469.2581	1471.6196
1471.8487	1472.3053	1484.6817
1505.0562	1552.6801	1574.7065
1577.6444	1578.0573	1589.1186
1589.5558	1591.1383	1591.8484
1591.9034	1599.2478	1603.9465
1604.7324	1605.7343	1606.4973
1608.9775	1609.8350	1612.0462
1613.3428	2955.2460	2991.2320
3025.1811	3029.5961	3031.0018
3066.7692	3094.4748	3095.5567
3096.0862	3096.2041	3096.4286
3096.7001	3097.1772	3097.6110
3097.7138	3104.5704	3106.4981
3106.7290	3106.7945	3109.0798
3109.6046	3109.8009	3109.9890
3111.0365	3115.2745	3116.1947
3116.6083	3117.0338	3118.6821
3119.0626	3120.9538	3121.4578
3121.5853	3122.6786	3124.6851
3127.2325	3127.9256	3129.0924
3129.5735	3130.1541	3133.3320
3139.5400	3144.1580	3144.8785
3146.3362	3158.4252	3187.8234

Int D iso1a S=5/2

-11.7991	-3.5122	20.4776
24.4555	28.0249	36.7252
39.1516	39.5950	43.1744
45.7392	49.3493	51.1003
55.4556	57.9530	59.7990
61.9613	63.6352	66.8784
69.5692	77.2146	81.0306
84.1394	93.6727	94.2740
96.6090	106.1802	109.8227
118.9771	123.6243	129.6396
136.3652	147.8204	156.5901
164.9343	171.7275	174.1709

181.0983	198.7031	205.3145
216.1489	218.6482	225.1074
227.9804	239.3983	239.9181
254.8443	260.1779	269.5923
270.2660	279.0840	282.1748
291.9771	293.6854	302.2672
311.6793	322.5690	328.5474
340.8465	342.2992	345.7028
357.7435	367.7418	387.5414
390.4156	395.0827	399.9506
401.3108	406.6328	408.7684
411.6630	416.2607	419.6634
447.9292	460.6538	468.7310
469.7848	474.1007	483.2198
504.2695	510.8810	513.5222
523.4682	532.0743	540.8534
545.1708	551.9050	567.1630
575.8284	611.4571	611.7749
611.9107	613.2429	614.3368
614.8680	621.9570	625.3807
630.1507	631.0458	632.4106
633.7063	635.6235	643.1923
652.8230	671.6759	686.8405
694.5207	697.2694	699.6727
702.1509	703.6837	706.6371
707.2657	724.6327	728.3348
737.1654	746.6348	750.2153
752.3452	753.7839	759.5452
763.0309	767.7399	776.2969
783.5217	786.2086	789.9431
803.1844	815.0894	825.6959
827.7591	833.2150	834.9922
836.6927	838.2776	843.2727
853.6354	860.3077	863.9489
880.5511	884.8164	894.6008
901.3906	904.0358	908.0964
912.4429	919.5392	923.9168
931.6775	936.9589	938.6709
939.3678	943.4371	951.1711
953.1726	953.7198	956.0501
960.0369	961.0615	961.8803
965.0332	966.5039	972.8209
975.1208	975.9569	977.9906
979.1892	981.5281	982.6031
987.1024	987.8244	988.4731
989.3442	990.2430	999.9383
1002.8996	1003.7174	1008.5140
1021.9146	1023.0286	1023.3657
1025.2390	1025.4527	1026.3963
1038.0103	1045.1026	1046.9699
1049.1685	1064.3120	1066.9060
1067.0406	1071.0379	1076.0342
1082.2462	1087.2943	1089.4879
1093.7411	1094.8648	1096.6505
1102.0808	1107.5452	1119.5008
1129.2772	1130.3487	1132.6071
1133.3778	1135.6807	1136.9671
1137.6424	1139.8321	1143.4070
1148.8370	1153.2748	1153.4712
1154.8158	1157.2801	1159.1950
1162.7342	1168.5627	1171.8028
1184.6867	1186.5201	1191.1142
1209.5528	1211.3189	1236.5079
1240.1988	1244.4990	1265.0922
1271.3089	1273.4429	1274.7468
1277.0313	1281.4783	1285.1111
1286.7224	1291.1844	1322.7836
1339.8059	1350.1189	1355.7662
1356.3277	1364.3998	1366.7882
1367.9585	1369.1583	1371.9719
1373.5792	1394.4769	1397.8271
1405.8920	1409.2273	1415.1665
1422.1940	1423.9534	1426.4474
1428.8224	1429.8967	1432.3656
1438.0626	1459.0157	1468.3972
1469.3118	1469.6114	1472.9281
1474.7633	1476.1900	1486.5312
1510.4248	1554.5507	1579.0674
1580.8784	1584.5977	1587.2472
1589.4016	1590.4216	1591.2615
1593.3127	1604.1477	1605.2042
1605.4081	1606.6772	1607.7527
1609.4004	1609.5328	1616.1742
1616.9563	2959.2781	3033.8419
3043.4643	3060.8648	3067.4697
3090.0553	3090.1382	3091.6596
3092.2912	3093.0774	3093.6476
3093.8237	3094.8350	3097.2076
3102.2085	3102.5466	3103.2158
3103.6673	3103.9295	3104.2350
3104.9791	3106.1465	3107.2150
3112.3496	3112.5274	3113.2726
3113.6293	3114.2642	3114.7254
3115.0759	3117.5135	3117.5450
3118.8217	3120.1204	3120.2910
3120.5244	3121.5069	3122.4896
3122.8635	3126.5230	3129.9438
3130.9813	3131.4773	3133.8644
3145.2623	3158.9406	3167.4575

Int D iso1b S=5/2

-13.4158	4.5577	9.3854
13.0171	21.9022	27.5449
30.0046	38.4651	40.0513
45.2951	48.8200	50.9299
53.4570	55.9099	60.8752
62.6525	64.7182	71.9353
76.2587	77.4995	81.5712
83.3700	85.9982	92.0332
95.3999	102.0971	109.9749
112.9381	117.6288	124.2948

132.3052	139.6348	150.7098
153.2110	173.8462	174.9176
186.4301	198.7436	205.1426
216.3817	225.5645	232.8396
233.7270	246.2560	248.4880
255.0923	261.9762	271.1182
271.8751	276.8530	284.2429
289.7919	302.9467	307.3214
313.7120	314.1808	336.3777
341.3448	346.7934	361.6118
368.6489	374.0124	398.3364
398.4422	403.6065	404.4056
404.8738	406.6254	410.8825
413.5419	419.7386	419.9798
434.4616	449.4090	463.4728
469.7939	479.4566	489.5881
495.3229	510.9819	512.9352
514.3047	530.4735	533.6219
542.5480	552.2357	566.9632
580.9508	605.3848	612.1405
612.3868	612.5968	613.6922
613.8670	615.4421	624.7701
625.4816	631.7727	632.6105
634.9658	636.4315	647.1140
649.6473	655.5070	687.6840
691.0503	698.7108	700.4658
700.6618	705.8201	706.5990
708.8130	722.6665	727.9989
736.5736	741.5628	749.0262
751.9717	756.3201	763.4499
767.9414	772.6654	777.3456
784.4401	788.0902	798.9462
804.1879	814.3726	825.3241
831.6600	832.3854	835.7358
836.0722	840.6712	842.1805
848.8322	864.1970	865.8624
883.2166	884.5750	903.5499
904.4832	907.2295	910.6551
913.5781	925.1253	931.2111
933.3215	936.9050	941.2820
942.8400	944.9625	952.7942
957.3595	960.2264	961.0998
961.5559	964.9162	967.6856
969.6982	971.9060	974.6654
974.6837	977.1745	979.0346
980.8874	982.7863	984.9250
987.6467	988.0358	989.8785
990.0676	990.8060	993.5662
999.6198	1003.0870	1011.2128
1021.6916	1022.6050	1023.6716
1023.9273	1025.4834	1025.5735
1043.5443	1047.6496	1048.4244
1055.8567	1063.5205	1064.4063
1070.3358	1071.3138	1071.8984
1087.9527	1089.0646	1093.7776
1094.9208	1096.3796	1097.4866
1104.4819	1107.7675	1121.5681
1125.3711	1128.7719	1131.9230
1134.0582	1136.2732	1138.1364
1138.8063	1140.9025	1142.9749
1151.7425	1153.8991	1154.7969
1157.3405	1157.8835	1158.2463
1160.6447	1167.2236	1170.9214
1182.7227	1186.3099	1187.9316
1208.0630	1228.3787	1236.5306
1241.9917	1265.4294	1266.6142
1270.8970	1272.1354	1273.5438
1283.3362	1284.5494	1286.1598
1290.2208	1292.0779	1324.2453
1343.7181	1345.7244	1348.4325
1355.4718	1357.0125	1367.4124
1368.9412	1370.2784	1370.7009
1378.4258	1392.2533	1393.7283
1410.1632	1414.4581	1417.9732
1419.6115	1423.1600	1426.1034
1428.4483	1429.8358	1430.2387
1441.9769	1456.7810	1466.8761
1469.3141	1470.4208	1470.7466
1472.3542	1472.9436	1487.0571
1507.1410	1549.4614	1576.3761
1577.3559	1579.7291	1588.3186
1590.5056	1591.2473	1591.5730
1593.5004	1603.4171	1603.6220
1603.9344	1604.7959	1605.2098
1608.0444	1610.0724	1611.2795
1614.0801	2960.5424	3035.9924
3049.7610	3056.0545	3069.1302
3088.2134	3090.8650	3092.3314
3092.4552	3093.5631	3094.2370
3097.9897	3098.6115	3099.5373
3101.9125	3101.9278	3102.6981
3103.5453	3104.5684	3104.8892
3105.0550	3106.0650	3109.7847
3111.0254	3111.6767	3111.7586
3112.1020	3115.7273	3116.0854
3117.0284	3118.2156	3119.2206
3120.9426	3121.8675	3122.1457
3123.1172	3125.8460	3126.6811
3126.7510	3134.4012	3136.3459
3137.0770	3144.0239	3145.0558
3148.5630	3151.8145	3154.1362

Int D iso2a S=5/2

5.7848	13.1685	17.8487
21.3189	28.3357	36.3527
40.6050	43.8958	47.2314
50.5902	52.5247	54.6511
57.7565	62.7420	63.2481
64.9510	69.9461	72.7078
76.3440	80.1021	82.0843
84.3698	86.6569	90.8725

92.9678	96.7835	100.1991
107.4784	115.4793	130.5483
133.1294	138.8801	146.7214
172.4117	174.0541	185.8164
188.1363	201.6245	202.3682
207.1516	215.9875	228.2213
237.7343	248.6013	251.3278
254.2020	261.9181	273.3159
274.0604	281.6307	288.3866
297.0563	298.1418	309.2246
323.4716	325.5710	337.8962
341.8463	343.0922	370.2232
373.1119	385.2039	399.9293
401.2431	401.7217	402.8561
404.1428	404.8922	406.1528
409.3340	414.0081	421.3262
440.4491	461.4670	462.2700
468.4468	487.3236	487.7552
504.5977	506.4846	508.0392
516.7170	524.5448	538.7731
543.4990	552.6269	569.4916
581.2660	606.8719	611.7793
612.0217	612.3742	613.6489
614.4202	615.5013	626.0835
627.7700	631.4698	632.2968
634.3405	636.5891	649.1299
652.8333	654.7487	689.3601
693.7588	696.7390	698.4219
700.4707	702.0072	708.1602
711.6302	719.8920	720.8601
728.1083	747.6527	750.4351
753.6120	758.4834	763.0726
764.8173	765.3412	775.4967
781.0098	789.5971	791.3707
794.7082	802.8148	821.5479
827.4087	830.7071	831.5795
834.0476	838.9608	843.0788
845.1864	865.8833	867.4853
881.6234	889.3949	901.5532
903.0826	904.0491	915.3791
920.4214	926.8123	930.6087
931.1202	937.8606	940.4153
943.1954	946.1008	954.4443
959.4958	961.3787	961.9905
962.3112	963.4004	966.0593
967.1524	970.0002	975.0767
976.8227	979.4843	980.6643
982.0669	984.8007	985.7920
987.0189	988.1191	988.6514
990.3523	991.2229	997.3579
998.9332	1003.3523	1013.8139
1020.9086	1022.1287	1022.5762
1023.8905	1024.2992	1027.3036
1043.0348	1043.7093	1047.4898
1053.0559	1057.4146	1065.0929
1067.1654	1068.5540	1070.1005
1080.0207	1087.6729	1089.4886
1092.8689	1093.6958	1098.6699
1106.6236	1122.5378	1126.9991
1129.0868	1131.8311	1132.3843
1133.2014	1135.6694	1136.9949
1140.0925	1144.1879	1145.6924
1147.8790	1151.7668	1154.1754
1155.6319	1156.6839	1157.6604
1158.0013	1173.7285	1176.9909
1177.7879	1182.1270	1189.2093
1210.5631	1238.6526	1243.6233
1250.1052	1261.8347	1264.5281
1267.8959	1273.9561	1276.5353
1279.3573	1280.4961	1283.6182
1285.3886	1290.6174	1323.8169
1343.7123	1345.0659	1346.9770
1361.3303	1361.7991	1367.7097
1368.5012	1370.4998	1371.8613
1378.4800	1392.6696	1394.9861
1408.2595	1417.6309	1420.5986
1422.5156	1423.9830	1426.1915
1430.0158	1430.3240	1430.9612
1443.0304	1455.2496	1466.5036
1467.2544	1470.1666	1470.4927
1471.7776	1474.6132	1487.0855
1504.5721	1547.3625	1573.5686
1576.9223	1583.8332	1588.5141
1591.4188	1591.6754	1592.0542
1594.6190	1601.5999	1603.4412
1605.1856	1606.1772	1606.3959
1608.1245	1610.4608	1611.8942
1617.6136	2960.0712	3039.0640
3040.8452	3051.6302	3063.7665
3067.8535	3092.9533	3093.1155
3093.8648	3094.8674	3095.1891
3096.9381	3098.1579	3100.0197
3102.0097	3103.2917	3103.6329
3104.4279	3106.4607	3106.8825
3108.6640	3109.7864	3111.8877
3112.4860	3113.4887	3114.2651
3114.7927	3115.1580	3116.2321
3117.0886	3117.7652	3120.7799
3120.9993	3121.9100	3122.5444
3124.6665	3125.1615	3126.5921
3127.1756	3127.5791	3127.9076
3135.6428	3136.7103	3137.6501
3139.0205	3152.7043	3159.3149

Int D iso2b S=5/2

-5.4738	20.0991	24.8211
32.9642	35.1323	40.9099
42.8284	51.1905	52.6830
53.6039	58.0855	59.8774
64.6682	69.8957	70.7216
71.3650	72.9950	75.0038

83.0524	86.2959	89.5661
90.9182	94.3739	98.3339
106.1691	110.4959	113.7082
118.6012	121.5376	124.7032
137.0264	150.6678	151.9855
170.4412	179.9353	183.8373
187.3152	202.8145	209.1823
214.8377	218.9050	228.7962
242.7847	248.3476	253.9472
257.2688	263.2553	273.3626
275.4769	276.7386	284.2811
289.2016	293.2821	309.1851
313.4338	313.9313	338.8972
343.3454	344.0774	363.1049
372.8689	373.6605	396.7698
397.4031	400.7655	402.2883
404.1431	410.9192	411.6993
414.5086	418.2819	422.9612
443.2131	445.0093	462.7719
470.8586	474.8516	484.6062
506.0204	509.9167	515.5101
519.4279	521.1173	540.5501
544.2743	553.5750	567.6562
583.6010	600.9697	612.1425
612.6529	613.0848	614.2505
614.7543	615.4370	625.5356
628.2553	632.3703	633.8581
634.6116	636.2874	646.4531
647.8364	653.1978	685.1515
688.3146	692.2041	699.1013
704.3239	706.8396	708.6395
711.3034	723.0601	726.6867
729.7424	748.2747	751.0742
752.8394	758.7482	759.7739
765.7160	771.6842	778.8791
783.4184	788.3751	790.0118
792.1529	805.0872	813.2716
823.3512	833.0396	835.6657
839.0727	843.8783	846.1826
848.4167	865.4813	868.2134
885.7923	886.5268	891.3366
902.5625	904.1043	905.1439
918.6714	926.4541	929.5819
934.6552	938.3424	943.6539
946.4130	949.8206	949.9207
956.4331	957.7494	959.8896
962.0549	963.2894	967.6152
970.3269	972.8943	974.4844
976.7011	978.7545	980.1126
981.1655	986.6663	987.3364
987.8375	989.0566	989.7757
989.9284	992.6590	993.5068
995.3964	1004.7503	1010.5432
1022.4701	1022.6686	1023.5078
1023.8215	1026.2522	1026.4822
1040.5390	1046.3931	1049.2248
1051.9859	1063.7305	1064.1111
1065.1654	1071.2100	1072.0081
1088.9614	1090.9591	1092.2878
1096.5196	1097.9400	1103.6568
1104.5992	1107.0678	1116.9506
1126.5393	1128.5171	1129.0056
1134.2189	1137.0858	1137.2237
1139.4244	1143.7113	1144.4245
1146.9430	1152.7065	1155.1221
1156.3129	1158.6786	1161.8933
1165.7315	1167.9660	1172.2002
1174.2184	1179.2507	1182.6608
1213.0619	1235.8958	1239.2122
1241.3429	1264.9114	1265.3120
1269.4209	1270.2318	1273.4917
1276.8339	1277.5376	1283.9643
1286.3112	1296.1433	1327.4369
1342.0334	1343.2507	1352.6868
1358.2889	1359.8425	1366.9906
1368.1594	1368.3796	1372.4993
1385.0516	1392.7642	1394.3498
1407.7535	1418.2106	1420.7283
1421.6223	1422.6367	1426.6410
1426.8593	1430.6180	1430.6898
1446.1577	1455.7809	1467.9237
1468.5340	1469.7543	1470.3376
1470.5898	1473.4064	1488.8808
1505.9745	1551.1580	1572.7465
1579.0147	1579.8582	1588.5600
1589.8869	1590.4142	1591.6131
1593.0890	1601.9925	1604.0658
1604.1104	1604.9036	1605.0103
1608.5623	1610.7709	1612.3700
1614.4298	2958.6800	3034.6295
3038.6163	3073.8461	3075.4119
3092.1650	3092.8212	3092.8565
3093.0605	3093.7302	3095.0530
3100.8864	3101.3981	3102.1272
3102.8619	3103.2672	3103.3298
3103.6715	3105.7262	3108.4333
3111.4649	3112.0552	3112.8508
3113.8287	3114.4972	3115.4490
3115.6796	3116.1529	3116.2090
3119.1073	3119.5533	3120.5206
3121.9872	3122.9722	3124.5644
3125.3505	3126.2018	3126.3131
3136.5113	3136.8112	3140.5164
3141.7442	3148.2846	3158.4831
3162.7276	3170.7046	3190.6989

TS B-C iso1a S=3/2

-463.9689	9.7267	16.8525
22.9292	25.6695	28.1496
32.8223	36.4119	41.7335
43.4304	46.1978	47.5872

50.9689 53.4124 60.9887
63.4693 65.1345 67.1394
73.0003 76.4083 81.8304
86.0572 90.6005 93.9078
95.1478 99.7373 106.3609
110.0824 117.6857 120.4833
131.4806 135.9730 152.9818
162.9940 170.3608 177.7892
181.2147 196.9902 203.7729
205.7704 218.5430 232.7993
238.4656 244.6626 248.6888
252.4659 262.3464 265.2016
271.4543 281.0368 285.4244
288.6413 300.5584 303.9655
311.8660 319.9160 321.9706
336.5853 347.8121 359.0502
366.5754 376.1646 385.8216
398.6478 400.3051 403.3982
404.3261 407.5649 410.1018
414.8229 417.5155 422.0040
425.4013 446.5871 450.1039
467.6427 475.4170 481.3805
493.7648 498.8537 506.0557
511.2472 516.9335 525.0111
535.8705 541.4178 546.3992
552.0528 574.3677 581.4172
609.4018 611.6526 611.9956
612.8856 614.0448 616.7617
621.8955 626.2778 629.7226
631.6664 633.2946 635.3204
637.5737 661.9827 663.8048
676.1941 692.2772 694.5141
698.0881 700.0425 701.8952
703.2299 707.7490 719.2675
727.5584 730.6708 736.1927
741.5632 747.5778 750.5558
752.5593 761.8330 763.7404
779.5981 780.6140 784.3246
790.8642 798.0834 800.2280
803.5243 819.6999 820.9385
822.6187 826.9776 832.2938
835.5906 835.9381 840.6047
855.0319 866.6287 881.8313
887.4724 899.3590 900.7600
910.5564 911.7934 918.6271
921.8898 926.2562 931.4113
934.4921 939.5304 941.5662
944.0390 954.0882 955.5205
956.3759 958.0670 960.6476
962.4248 964.5826 966.4189
971.4954 973.9232 975.3097
976.9104 979.5382 979.7497
980.8268 981.4391 982.8620
984.4175 986.7563 987.7447
988.9328 990.0608 1001.4205
1004.6987 1010.5345 1018.1588
1022.7595 1024.6730 1025.2894
1026.4579 1027.1484 1040.9125
1043.8740 1044.9937 1046.7416
1058.2011 1059.9573 1073.9289
1074.4750 1076.8889 1080.9533
1084.0174 1087.6691 1089.8696
1092.8274 1095.6257 1100.1198
1132.0884 1132.7680 1133.2090
1135.6981 1135.9856 1137.5950
1141.1739 1145.6002 1146.4500
1150.5911 1151.6412 1152.3723
1153.8421 1154.1858 1156.5050
1157.7977 1162.7252 1171.4898
1186.1189 1195.7907 1203.9506
1208.0051 1210.3464 1238.4101
1239.7873 1240.4231 1262.7029
1262.9661 1267.7767 1277.0188
1278.4171 1285.5180 1285.8346
1288.7639 1292.6425 1330.5051
1341.1687 1344.0687 1351.7452
1359.0320 1362.8071 1365.6758
1367.6922 1368.4471 1371.1564
1386.3264 1393.0053 1398.1149
1407.0452 1417.0593 1421.1881
1426.0376 1427.7860 1428.9917
1429.4694 1432.1561 1434.5668
1457.0187 1463.3056 1469.2409
1471.5391 1472.3490 1472.6387
1473.1609 1473.2951 1502.1772
1508.5395 1558.5262 1566.2400
1575.3654 1577.6302 1583.2932
1586.8597 1590.2932 1591.3636
1591.9776 1594.8490 1605.1902
1606.0417 1606.8138 1607.4786
1608.1957 1610.8528 1611.6682
1620.7093 2957.3838 3031.5421
3054.9286 3062.2561 3063.0856
3091.2426 3093.7673 3096.2574
3096.9341 3097.2870 3097.4319
3098.4544 3100.0088 3101.3854
3103.1089 3103.3771 3103.5225
3106.0288 3106.1476 3107.3536
3109.1692 3110.9230 3112.9013
3113.4887 3116.6548 3117.2097
3117.8746 3118.5678 3119.5833
3119.8265 3119.9468 3120.1865
3120.8571 3121.8210 3124.5897
3125.0989 3125.7927 3126.5214
3128.1039 3129.4826 3135.4099
3136.4698 3138.9435 3144.2216
3145.3532 3154.8231 3170.4524

TS B-C iso1b S=3/2

-404.0319 -20.4219 5.7048
10.8771 17.7295 27.1396

28.6438	34.4439	38.9582
41.5835	46.7227	53.4339
58.2989	62.0878	63.8404
67.0182	72.2633	73.4328
79.0467	80.7692	85.0159
85.2769	92.8310	102.6183
104.8449	106.0774	110.6688
112.6817	116.5696	131.3290
134.4789	140.0687	147.6159
152.4237	172.2237	173.8279
181.7624	193.6069	196.9559
206.5021	219.1358	227.7991
243.4869	246.3384	250.0608
252.5439	260.0332	261.9251
269.7144	281.0080	287.9256
289.1283	299.6078	305.3494
316.6867	323.2670	325.6138
343.3308	343.6788	361.6095
365.4373	380.3016	392.2593
399.0825	401.7050	402.9242
408.3220	409.5129	409.9847
411.0153	414.0354	419.1621
426.2405	446.6377	455.2445
464.6381	472.6397	482.9728
486.7771	497.3017	504.7790
507.7659	520.3483	528.5015
535.7726	537.2421	543.9609
551.5644	552.5256	581.0672
609.0779	611.7213	611.9326
612.2162	614.0097	616.2095
623.1385	625.5732	628.6670
631.9912	632.6866	635.0868
637.4380	662.3195	664.2533
674.6158	691.4804	694.5008
697.5709	699.5448	703.0511
703.9978	714.0882	722.3994
732.7749	735.7989	739.0829
744.0733	747.6893	750.1355
753.7394	759.0019	765.6812
779.2794	784.6855	786.7823
789.6747	801.1523	802.8391
811.2335	812.8196	820.9897
821.6193	829.5195	832.0057
834.6533	836.5955	842.1415
856.8082	867.4996	875.9865
888.2486	897.7812	903.6501
908.2621	915.7982	918.6096
920.8743	924.8313	932.9265
934.0284	939.3992	942.9823
944.1993	948.4056	955.6574
957.7369	959.2099	962.1217
963.6851	963.9764	967.0849
973.8002	974.8476	976.3856
977.1703	978.8781	979.8794
981.7397	982.2450	983.4058
984.0965	987.2092	988.4784
989.2223	990.3985	1002.7510
1004.8611	1010.4241	1019.7080
1022.7937	1023.8360	1024.8681
1025.2808	1027.1847	1030.9080
1046.5086	1046.8842	1055.6057
1060.3744	1064.3266	1072.3892
1073.2841	1084.3509	1086.4916
1088.1889	1089.6823	1090.9686
1095.5041	1095.7488	1101.3661
1131.4469	1132.6364	1133.1108
1133.6515	1134.9600	1138.6090
1139.0622	1145.4782	1146.6861
1151.4606	1152.1941	1153.2745
1154.5366	1155.5875	1157.8874
1158.3972	1163.3263	1176.7098
1185.2040	1199.6756	1202.2139
1206.4385	1208.1251	1233.9932
1236.5238	1242.8685	1264.7235
1264.8397	1267.8932	1275.1523
1279.7138	1282.4495	1283.7099
1287.5199	1293.1242	1329.1646
1342.7140	1343.3905	1348.4796
1356.5317	1366.8705	1368.1877
1369.7140	1371.2024	1371.4079
1393.0991	1394.1789	1396.4623
1410.1404	1418.7128	1419.5216
1426.0671	1427.7901	1428.9441
1431.9076	1432.0066	1434.0238
1456.5823	1465.1792	1468.4110
1470.1108	1472.3669	1473.1121
1473.6312	1475.1439	1506.1555
1508.6021	1558.5124	1566.0109
1574.7587	1576.4753	1582.7782
1588.0891	1589.5125	1591.7754
1592.7168	1596.0725	1605.7482
1606.4646	1606.5953	1607.0629
1607.6635	1610.7147	1611.9346
1620.4896	2957.8184	3032.0140
3034.6203	3044.6891	3065.5089
3093.8471	3094.2555	3095.6169
3096.0247	3096.1214	3096.4510
3097.0030	3097.1460	3102.6700
3103.9022	3105.1148	3105.3670
3106.1875	3106.6740	3106.7201
3108.8578	3110.7371	3114.1471
3114.3325	3117.5834	3117.9489
3118.1234	3118.1687	3119.5132
3120.3345	3120.7298	3121.3666
3123.4730	3124.5151	3125.8498
3127.6370	3127.9290	3128.0844
3129.1128	3130.1634	3133.4383
3135.7847	3144.2027	3146.7177
3151.2405	3153.1829	3155.4110

TS B-C iso2a S=3/2

-464.1640	9.7286	16.8544
22.9394	25.7708	28.2648
32.8234	36.4331	41.7367
43.4338	46.2016	47.5920
50.9737	53.4258	60.9897
63.4688	65.1360	67.1471
73.0040	76.4134	81.8389
86.0604	90.5913	93.9149
95.1540	99.7541	106.3608
110.0907	117.6857	120.4950
131.4859	135.9801	152.9850
162.9992	170.3650	177.7923
181.2147	196.9899	203.7746
205.7752	218.5407	232.7990
238.4624	244.6662	248.6911
252.4634	262.3461	265.2047
271.4571	281.0419	285.4246
288.6511	300.5592	303.9664
311.8686	319.9131	321.9718
336.5909	347.8109	359.0498
366.5755	376.1647	385.8234
398.6542	400.3103	403.3991
404.3289	407.5657	410.1027
414.8255	417.5230	422.0042
425.4047	446.5917	450.1029
467.6402	475.4206	481.3854
493.7697	498.8524	506.0630
511.2477	516.9353	525.0073
535.8704	541.4160	546.3975
552.0520	574.3794	581.4193
609.4014	611.6524	611.9957
612.8851	614.0444	616.7618
621.8982	626.2789	629.7217
631.6655	633.2955	635.3199
637.5735	661.9855	663.8052
676.1946	692.2783	694.5133
698.0878	700.0382	701.8945
703.2282	707.7501	719.2694
727.5605	730.6705	736.1938
741.5653	747.5799	750.5553
752.5602	761.8315	763.7399
779.6001	780.6147	784.3297
790.8594	798.0875	800.2280
803.5376	819.7138	820.9392
822.6250	826.9748	832.2964
835.5886	835.9407	840.6096
855.0324	866.6290	881.8303
887.4734	899.3608	900.7600
910.5565	911.7927	918.6252
921.8890	926.2580	931.4107
934.5021	939.5317	941.5671
944.0378	954.0912	955.5324
956.3754	958.0690	960.6460
962.4294	964.5985	966.4222
971.4943	973.9236	975.3084
976.9096	979.5364	979.7497

980.8278	981.4397	982.8632
984.4161	986.7560	987.7444
988.9330	990.0600	1001.4161
1004.6991	1010.5308	1018.1601
1022.7591	1024.6723	1025.2884
1026.4578	1027.1474	1040.9241
1043.8746	1045.0092	1046.7465
1058.1965	1059.9537	1073.9283
1074.4738	1076.8903	1080.9589
1084.0193	1087.6658	1089.8690
1092.8329	1095.6250	1100.1217
1132.0887	1132.7674	1133.2088
1135.6985	1135.9858	1137.5946
1141.1738	1145.6006	1146.4486
1150.5919	1151.6430	1152.3755
1153.8413	1154.1854	1156.5064
1157.7989	1162.7262	1171.4981
1186.1231	1195.7986	1203.9441
1208.0072	1210.3466	1238.4115
1239.7849	1240.4234	1262.7084
1262.9688	1267.7762	1277.0181
1278.4175	1285.5171	1285.8335
1288.7656	1292.6431	1330.5034
1341.1709	1344.0669	1351.7454
1359.0233	1362.8034	1365.6720
1367.6923	1368.4468	1371.1523
1386.3227	1393.0050	1398.1145
1407.0775	1417.0606	1421.1766
1426.0388	1427.7846	1428.9925
1429.4683	1432.1560	1434.5653
1457.0179	1463.3040	1469.2407
1471.5393	1472.3490	1472.6393
1473.1625	1473.2929	1502.1721
1508.5393	1558.5258	1566.2386
1575.3636	1577.6309	1583.2954
1586.8571	1590.2937	1591.3630
1591.9753	1594.8467	1605.1909
1606.0424	1606.8115	1607.4795
1608.1931	1610.8512	1611.6683
1620.7095	2957.3585	3031.5298
3054.9246	3062.2665	3063.1119
3091.2421	3093.7651	3096.2582
3096.9332	3097.2868	3097.4326
3098.4549	3100.0145	3101.3855
3103.1075	3103.3757	3103.5207
3106.0290	3106.1485	3107.3544
3109.1682	3110.9225	3112.8967
3113.4899	3116.6535	3117.2084
3117.8735	3118.5625	3119.5803
3119.8154	3119.9524	3120.1859
3120.8534	3121.8226	3124.5892
3125.0955	3125.7908	3126.5206
3128.1083	3129.4886	3135.4155
3136.4400	3138.9385	3144.2344
3145.3793	3154.8263	3170.4589

TS C-D iso1a S=5/2

-430.8311	7.6283	15.3291
17.8395	22.8943	25.8188
27.9209	30.6684	39.1309
43.9406	45.9032	47.1422
51.9133	56.0559	57.5847
61.2267	66.8750	67.7381
70.7271	76.5933	81.4826
85.9004	89.2296	92.7524
94.4605	98.0787	105.7508
108.7176	112.5033	119.9832
136.1450	142.4970	148.8249
154.7918	166.1094	173.3803
179.3888	189.5763	194.6472
214.5919	218.8671	223.9451
235.6151	241.7879	247.8889
251.5652	262.5250	268.3909
269.4450	274.4142	280.7957
288.3335	295.3171	299.4530
319.3859	320.4263	325.4115
340.2118	342.7981	351.9371
366.1423	382.4363	384.7949
399.2342	402.0823	404.9798
407.0169	407.8440	408.8798
413.3806	416.4540	420.3092
429.5447	438.7310	462.0439
463.6609	473.9340	474.2436
489.8791	492.2814	501.2236
513.0666	515.4902	528.7045
537.6340	542.8081	553.2361
553.4106	576.4167	606.3862
609.8203	610.8966	612.1707
613.0431	614.4595	614.9548
623.3714	625.3927	630.9692
632.2260	634.0906	635.8785
650.9141	651.7190	656.1600
683.8611	688.7654	695.3188
696.7049	698.6079	703.2895
705.1240	712.7027	724.4146
733.0628	736.9968	746.0349
750.5182	750.7277	753.5761
756.5896	765.7418	776.1526
779.1806	780.1768	782.7515
795.6119	800.7686	819.6371
820.9095	822.7118	829.7334
830.2394	833.3918	838.1553
838.9513	859.7955	860.0587
864.4064	877.3339	881.5049
895.9903	906.9592	908.7350
914.0437	917.9629	921.8447
931.0831	938.7569	940.7604
941.7176	942.0144	952.7298
953.8135	957.1888	960.6513
961.6896	964.9445	965.0289
967.0098	972.3627	973.8759
976.6878	977.1211	978.2109
980.3357	981.6819	984.1803
985.9369	986.2778	986.4975
987.0867	988.7695	989.2689
993.4466	1003.6116	1003.8378
1011.3551	1019.6882	1022.8875
1024.0766	1024.3013	1025.0022
1025.2494	1025.8955	1044.6483
1047.2119	1066.0789	1069.9074
1071.4427	1072.0486	1074.5449
1083.0711	1087.5766	1093.1878
1093.8656	1094.6211	1107.0213
1108.2919	1119.4616	1130.9505
1131.3332	1132.0320	1132.5234
1135.7113	1136.8517	1137.7601
1141.2462	1142.8364	1143.6466
1147.3506	1151.7855	1153.2099
1154.0100	1155.7827	1160.2939
1161.9073	1162.3752	1163.9452
1171.6047	1191.5471	1207.4997
1221.6977	1233.3850	1234.2576
1240.9957	1246.1094	1264.4327
1264.5778	1268.1161	1271.9590
1273.3814	1280.6467	1282.2531
1286.4443	1291.5346	1326.3611
1343.3087	1343.6844	1346.0634
1358.1914	1366.6563	1366.8389
1368.5010	1370.2793	1373.1210
1391.6032	1396.0247	1396.9365
1409.2306	1418.8943	1420.2042
1420.6596	1424.6610	1425.5493
1426.2782	1430.0767	1430.5050
1447.6450	1455.9717	1466.4044
1467.1246	1470.5688	1471.0367
1471.0591	1471.3265	1489.6454
1508.5400	1557.8052	1574.3053
1575.1354	1576.0432	1580.1516
1585.7273	1587.1733	1589.6545
1590.0378	1602.2200	1602.8847
1603.2429	1603.7242	1604.5052
1605.1034	1608.5190	1609.7576
1619.2563	2959.5287	3013.9257
3034.9399	3059.3601	3067.6915
3085.9546	3089.1678	3091.4221
3091.5511	3092.2139	3092.8406
3095.5980	3096.1264	3096.4852
3098.8572	3100.3018	3100.6088
3101.2179	3102.0458	3103.7152
3104.2729	3105.1892	3106.3685
3108.8095	3108.8691	3109.6938
3110.7829	3111.8905	3112.9903
3113.8182	3114.9319	3115.4702
3118.2180	3119.5947	3120.1354
3120.2121	3120.2398	3120.9300
3123.0579	3123.9590	3124.2679
3125.0399	3128.8077	3130.3334

3136.4611	3147.7452	3156.6315

TS C-D iso1b S=5/2

-393.0795	5.8123	7.8846
10.9416	21.3006	27.7471
32.5210	34.0124	38.4219
46.9856	48.9481	51.8384
54.3977	58.4603	63.0672
66.5834	70.5285	71.6459
74.7113	80.9720	83.1148
88.3385	90.0573	93.4380
95.2560	103.4511	106.8532
110.2248	115.0663	122.1441
131.0130	138.3483	152.7497
158.5296	169.4813	173.9717
177.2379	184.0757	196.4221
208.3344	220.0253	227.6512
237.3872	245.2029	251.0613
252.6368	262.2046	264.4785
269.4678	274.9425	280.5525
291.7505	295.6385	298.1778
313.5843	322.0677	335.6404
338.2926	339.8664	357.4640
360.0077	378.3350	387.5822
399.9453	400.8477	404.8544
405.5837	407.8568	413.4296
416.0249	424.5425	427.0526
431.5273	439.5072	463.9370
471.7272	475.9773	478.6359
489.5516	492.4833	504.0552
512.8469	517.9136	528.8742
535.4611	544.3179	553.4292
555.7381	579.1531	608.7911
609.8079	611.9985	612.8745
613.9751	616.2087	616.7148
621.9419	625.0580	630.1697
632.9600	633.6507	635.1220
650.4429	653.8191	657.8439
679.9403	685.8597	688.7681
696.4910	699.6192	704.8361
708.4389	711.7605	721.8763
731.3901	738.2832	747.1902
750.9958	751.9634	754.0774
757.0086	765.5658	772.9081
779.5477	780.2506	780.9431
799.5038	801.6223	818.8953
823.2993	827.4682	829.6488
831.3923	834.4125	838.8006
845.5175	861.5722	862.8316
864.8785	874.9477	881.6896
897.1095	904.1562	915.8396
916.9434	919.1817	926.0901
933.1249	941.0086	941.3509
942.3726	945.9052	951.1173
954.2757	960.8949	961.3670
963.8745	966.2479	968.5429
969.7708	972.4736	973.0220
973.1967	974.6092	976.6338
977.0620	982.9238	983.6248
985.1685	986.4161	986.7380
988.7148	989.1358	991.0766
993.6870	1004.7591	1005.1511
1011.6825	1022.8946	1023.0759
1023.8741	1024.3220	1025.8193
1026.0999	1033.7968	1044.3662
1048.1566	1067.9979	1069.7682
1072.9782	1073.5466	1080.0054
1085.5214	1087.8018	1091.9948
1092.9804	1094.1491	1101.3091
1114.6518	1116.9878	1131.5506
1131.6242	1131.9003	1133.3188
1136.6958	1137.6010	1138.3831
1141.9190	1144.5327	1147.9715
1149.7203	1151.7917	1152.9010
1153.8177	1159.0297	1159.2546
1164.0557	1165.1954	1167.3701
1170.3226	1184.1665	1207.3551
1217.2869	1222.2040	1235.5034
1241.6056	1244.9601	1264.3492
1264.7323	1268.1081	1273.4049
1274.2766	1282.4491	1283.6415
1284.4662	1296.1014	1328.3943
1341.3427	1342.7842	1344.2215
1359.9654	1366.2534	1367.8678
1369.1151	1371.3247	1372.1810
1392.7616	1394.7335	1395.0767
1407.4816	1419.7770	1421.2400
1422.2622	1424.1600	1425.5902
1425.9745	1428.9483	1430.7514
1446.8040	1455.2073	1465.4281
1467.8180	1469.0714	1469.9921
1470.6404	1472.2791	1491.1456
1508.9401	1558.4248	1573.9221
1574.2283	1575.4216	1579.3873
1585.2122	1586.2874	1588.6157
1591.3724	1601.3832	1602.5305
1602.6267	1603.2000	1603.8956
1604.6065	1607.3619	1610.5477
1620.1135	2961.1695	3016.9383
3034.8638	3067.4208	3070.3890
3090.9802	3091.1377	3091.2489
3091.7059	3091.8949	3095.2391
3095.4994	3098.9437	3099.3564
3099.9190	3101.7663	3103.0660
3103.2645	3103.4371	3103.9342
3106.3509	3106.6440	3107.0513
3108.2272	3110.0582	3111.6256
3114.4623	3114.6590	3115.9708
3117.5556	3118.7788	3118.9949
3119.6085	3120.0340	3120.4209
3121.5439	3121.6125	3123.3509

3124.2586	3124.9960	3125.1518
3129.5702	3132.8884	3134.7575
3138.3554	3145.4347	3151.6213

TS C-D iso2a S=5/2

-430.8244	7.6296	15.3327
17.8438	22.9015	25.8378
28.0266	30.6736	39.1312
43.9406	45.9017	47.1428
51.9135	56.0560	57.5832
61.2268	66.8767	67.7370
70.7257	76.5935	81.4819
85.8995	89.2271	92.7495
94.4595	98.0804	105.7491
108.7166	112.5005	119.9816
136.1448	142.4977	148.8230
154.7902	166.1106	173.3806
179.3892	189.5755	194.6473
214.5916	218.8657	223.9445
235.6126	241.7872	247.8873
251.5644	262.5246	268.3883
269.4439	274.4133	280.7951
288.3338	295.3194	299.4526
319.3864	320.4287	325.4107
340.2116	342.7988	351.9382
366.1435	382.4383	384.7984
399.2356	402.0829	404.9781
407.0149	407.8429	408.8792
413.3797	416.4521	420.3079
429.5450	438.7300	462.0437
463.6600	473.9339	474.2433
489.8798	492.2811	501.2242
513.0673	515.4893	528.7048
537.6327	542.8080	553.2359
553.4104	576.4166	606.3891
609.8205	610.8968	612.1707
613.0431	614.4594	614.9548
623.3715	625.3939	630.9691
632.2261	634.0906	635.8788
650.9145	651.7207	656.1607
683.8606	688.7660	695.3149
696.7058	698.6075	703.2880
705.1234	712.7026	724.4148
733.0632	736.9970	746.0352
750.5181	750.7276	753.5758
756.5895	765.7418	776.1529
779.1805	780.1762	782.7521
795.6039	800.7752	819.6353
820.9056	822.7096	829.7324
830.2362	833.3917	838.1539
838.9503	859.7951	860.0554
864.4065	877.3325	881.5038
895.9899	906.9598	908.7351
914.0437	917.9623	921.8443
931.0827	938.7564	940.7601
941.7181	942.0182	952.7287
953.8132	957.1888	960.6515
961.6899	964.9439	965.0439
967.0042	972.3621	973.8763
976.6879	977.1211	978.2112
980.3343	981.6813	984.1793
985.9361	986.2776	986.4973
987.0869	988.7688	989.2683
993.4422	1003.6115	1003.8355
1011.3498	1019.6878	1022.8874
1024.0764	1024.3016	1025.0024
1025.2489	1025.8948	1044.6486
1047.2113	1066.0794	1069.9076
1071.4441	1072.0487	1074.5452
1083.0718	1087.5762	1093.1880
1093.8686	1094.6212	1107.0210
1108.2917	1119.4591	1130.9508
1131.3335	1132.0321	1132.5234
1135.7119	1136.8512	1137.7600
1141.2465	1142.8365	1143.6465
1147.3508	1151.7866	1153.2098
1154.0097	1155.7833	1160.2928
1161.9072	1162.3746	1163.9451
1171.6038	1191.5480	1207.5017
1221.6978	1233.3831	1234.2575
1240.9956	1246.1088	1264.4330
1264.5776	1268.1163	1271.9594
1273.3811	1280.6474	1282.2536
1286.4448	1291.5348	1326.3617
1343.3087	1343.6954	1346.0644
1358.1731	1366.6568	1366.8384
1368.5005	1370.2798	1373.1208
1391.5990	1396.0246	1396.9370
1409.2566	1418.8935	1420.2044
1420.6471	1424.6611	1425.5494
1426.2780	1430.0767	1430.5050
1447.6451	1455.9717	1466.4047
1467.1252	1470.5690	1471.0371
1471.0591	1471.3268	1489.6454
1508.5400	1557.8053	1574.3048
1575.1356	1576.0432	1580.1502
1585.7277	1587.1736	1589.6549
1590.0378	1602.2197	1602.8849
1603.2428	1603.7242	1604.5054
1605.1034	1608.5190	1609.7577
1619.2563	2959.5107	3013.9264
3034.9401	3059.3627	3067.6959
3085.9525	3089.1653	3091.4223
3091.5516	3092.2150	3092.8412
3095.5986	3096.1253	3096.4864
3098.8568	3100.3026	3100.6094
3101.2131	3102.0474	3103.7147
3104.2740	3105.1856	3106.3686
3108.8090	3108.8692	3109.6936
3110.7828	3111.8998	3112.9906
3113.8193	3114.9321	3115.4704

3118.2183	3119.5949	3120.1356
3120.2124	3120.2399	3120.9298
3123.0572	3123.9593	3124.2669
3125.0401	3128.8069	3130.3334
3136.4627	3147.7459	3156.6260

5. Table S4. Crystal and refinement data for **2**.

	Mn[O-terphenyl-O]^{Ph}(THF)$_2$
formula	$C_{52}H_{48}MnO_4 \times CH_2Cl_2$
fw	875.76
crystal system	Orthorhombic
space group	$P2_12_12_1$
a (Å)	16.9723 (17)
b (Å)	17.3689 (15)
c (Å)	14.5364 (13)
α (deg)	90.00
β (deg)	90.00
γ (deg)	90.00
V (Å^3)	4285.2 (7)
D_c (g cm^{-3})	1.357
Z	4
μ (mm^{-1})	0.483
T (K)	100(2)
R_1	0.0893
GOF	1.121
